# Online Health Literacy Resources for People With Intellectual Disability: A Grey Literature Scoping Review

**DOI:** 10.1111/jir.70028

**Published:** 2025-08-02

**Authors:** Jessica Keeley, Zhenmei Yeap, Rachel Skoss, Thomas Nevill, Susan Hunt, Jacinta Saldaris, Jenny Downs

**Affiliations:** ^1^ The Kids Research Institute Australia The University of Western Australia Perth Australia; ^2^ Psychological Science The University of Western Australia Perth Australia; ^3^ Microboards Australia Perth Australia; ^4^ Institute for Health Research The University of Notre Dame Australia Fremantle Australia; ^5^ School of Population and Global Health The University of Western Australia Perth Australia; ^6^ School of Physiotherapy and Exercise Science Curtin University Perth Australia

**Keywords:** health information, health literacy, Intellectual disability, scoping review

## Abstract

**Background:**

People with intellectual disability experience higher rates of physical and mental health problems than those without intellectual disability. Health literacy includes accessing, understanding, appraising and applying health information. Improving health literacy is associated with better health outcomes. The internet is a primary source of health information for many people. This study aimed to evaluate available online health resources for people with intellectual disability and their families to understand information gaps.

**Method:**

A scoping review of grey literature was conducted using two searches: a targeted search of disability organisation websites and an advanced Google search using key terms. The methods were guided by a modified version of Arksey and O'Malley's methodological approach.

**Results:**

A total of 1165 health resources for people with intellectual disability and their families were identified. Tests, checks and procedures were the most common category of health addressed (*n* = 227, 19.5%) and most content was directed at the individual with intellectual disability (*n* = 837, 71.9%). Resources addressed the health literacy domains of accessing (*n* = 1165, 100%) and understanding (*n* = 1132, 97.2%) information more often than appraising (*n* = 575, 49.5%) and applying (*n* = 415, 35.6%).

**Conclusions:**

Improving the health literacy of people with intellectual disability is an important part of addressing health disparities and requires understanding of the available information. Some information gaps were identified, including limited sexual health resources and mental health resources for adolescents.

## 1 Introduction

Many people with intellectual disability experience complex health needs. Comorbidities and chronic health conditions are common and occur more frequently in people with intellectual disability than those without (e.g., diabetes, epilepsy and asthma; Liao et al. 2021). A population study from Scotland found that physical health and mental health were poorer in people with intellectual disability compared with those without (Hughes‐McCormack et al. 2017). A systematic review reported that nearly 40% of children and adolescents with intellectual disability experienced mental health difficulties (Buckley et al. 2020).

Poorer health status may result in higher rates of hospitalisation for children and adults with intellectual disability compared with those without intellectual disability (Dunn et al. 2018). Many of these hospitalisations are potentially preventable (Balogh et al. 2010; Weise et al. 2021), including those related to conditions such as epilepsy and vaccine‐preventable illnesses (Weise et al. 2021). A large study from England found that between 2000 and 2019 the death rate for people with intellectual disability was three times higher than that of people without, with some causes of death being significantly more frequent for people with intellectual disability (Tyrer et al. 2022). For example, epilepsy‐related deaths were found to be 23 to 53 times higher for people with intellectual disability than those without.

This serious and significant health disparity signifies the need for healthcare practice strategies and policy reforms to ensure that people with intellectual disability can live with optimal health and well‐being (Emerson 2021). Current inequalities suggest violations of the United Nations Convention on the Rights of Persons with Disabilities (UNCPRD; 2006, Article 25) which states that people with disability have the right to access the same quality of healthcare as people without disability. The Australian Government has recently implemented a *National Roadmap for Improving the Health of People with Intellectual Disability*, acknowledging the health inequalities experienced by people with intellectual disability and seeks to address these by focusing on improving information, understanding and support (Australian Government 2021).

One factor that has been identified as protective against poorer health outcomes and increased hospitalisations is health literacy (Shahid et al. 2022). Health literacy refers to competencies in the domains of accessing, understanding, appraising and applying health information (Sørensen et al. 2012). The domains apply from individual to population levels and across healthcare settings (Sørensen et al. 2012). For people with intellectual disability, health literacy tends to require an interactive approach where family members, other supporters (e.g., care workers) and healthcare professionals often play a significant collaborative role (Chinn 2014; Geukes et al. 2018). Health literacy can be influenced by factors including employment status, education and household income and has been shown to correlate with poor health literacy (Shahid et al. 2022). In practice, poor health literacy can present as difficulties understanding healthcare professionals and patient education materials, as well as challenges accessing healthcare services (Magnani et al. 2018). This can result in poorer health outcomes, higher mortality, increased hospitalisations and decreased proficiency administering medication (Berkman et al. 2011). Research from the United States found that hospitalised patients with low health literacy were three times more likely to revisit the emergency department than those with higher health literacy (Shahid et al. 2022). Higher levels of health literacy increase opportunities for healthy behaviours that likely improve health outcomes. For example, Wolf et al. (2016) demonstrated that targeted training on how to read medication labels could improve compliance/adherence to treatment for adults with type 2 diabetes or hypertension. While this population did not have a cognitive impairment, it suggests the focus on increasing understanding has the potential to change behaviours, relevant also for people with intellectual disability.

Accessing health information has been identified as important to people with intellectual disability, but some barriers can be experienced (Doherty et al. 2020). Examples of barriers include limited information availability and support to find and understand information (Doherty et al. 2020). If information is available, a lack of opportunities to understand, appraise and then apply the health information to healthcare can pose difficulties (Alshammari et al. 2018; Doherty et al. 2020; Matin et al. 2021).

The internet is a commonly used source of health‐related information (Jia et al. 2021). There is limited recent research exploring how people with intellectual disability use the internet in health contexts (Kuruppu Arachchi et al. 2023). One small Australian study including 39 adults (19–59 years) with intellectual disability explored how the internet is used to access health‐related information (Kuruppu Arachchi et al. 2023). This study used structured interview survey methods and found that participants tended to search for information about healthy lifestyle (e.g., healthy recipes and gym information) and learning about conditions and illnesses experienced by themselves and their loved ones. Most participants (*n* = 33) used the internet independently without a supporter and reported that this gave them a sense of achievement and connectedness with others with similar concerns. While the generalisability of these findings is limited due to participants having less severe impairment (33 could read and 15 had completes vocational education out of 39 participants) and the small sample size, this study provides insight into how some people with intellectual disability use the internet to inform their health.

People with intellectual disability experience barriers to accessing the internet and online resources (Alfredsson Ågren et al. 2020; Chadwick et al. 2013). These include barriers that are financial (due to economic disadvantage), social (lack of consideration for content accessibility), political (limited governmental inclusion strategy) and educational (lack of training for individuals and supporters; Chadwick et al. 2013). Research from Sweden compared the internet use of adolescents with (*n* = 114) and without (*n* = 1161) intellectual disability, finding that those with intellectual disability were significantly less likely to have access to internet‐enabled devices and perform activities on the internet (Alfredsson Ågren et al. 2020). Further, only 20% of adolescents with intellectual disability used the internet to search for information, compared with 86% of those without. Restraints that prevent full access to the internet and resources limit how information can be identified and applied in health and other contexts.

### 1.1 The Present Study

Improving the health literacy of people with intellectual disability can work towards addressing the significant health disparities experienced by people with intellectual disability. It is therefore important to understand what health information is available to people with intellectual disability online. However, there is limited research on how people with intellectual disability use the internet for healthcare purposes. While many of the resources directed at people with intellectual disability are developed by non‐government and community sector organisations, as yet, there are no grey literature reviews or mapping exercises conducted to determine what resources are collectively available for this population. Further, grey literature reviews are increasingly being recognised as valuable in academic literature (Paez 2017) and used in the field of health (Braithwaite et al. 2019; Brennan et al. 2023). The aims of this study are to understand what health literacy resources are available online for people with intellectual disability and their supporters (e.g., family and paid carers), how the information is presented and to identify and highlight gaps in the available information. We approach this aim by evaluating what Australian families would find if they searched for health information (including international resources) online as an example of available resources.

## 2 Methods

The protocol for this scoping review is published (Yeap et al. 2024). Two parents of children with intellectual disability with different impairment levels and internet use informed the design, implementation, analysis and write‐up of this study. In practice, this involved having significant input into planning the scope and methods of the study (including selecting the spreadsheet categories), contributing to the list of organisations (described below), consulting on the organisation of resources and providing feedback and edits on the manuscript. Including the perspectives of families who tend to play an important role in the lives of people with intellectual disability is an important step to ensure that the work is appropriate and meaningful.

A scoping review of grey literature was considered the most appropriate design for this study to map available relevant information and because it is adaptable for use with online resources. The Arksey and O'Malley (2005) methodological approach for conducting scoping reviews informed this study. This framework comprises five stages including (1) identifying the research question, (2) identifying relevant studies, (3) study selection, (4) charting the data and (5) collating, summarising and reporting the results. Adaptations to this process allowed for the inclusion of online resources as opposed to peer‐reviewed or other grey literature (e.g., government reports and dissertations) data. These deviations are described below and include changing references from ‘studies’ to ‘resources’. The PRISMA Scoping review extension guided the process of conducting and reporting on this review (Tricco et al. 2018). For example, data were collected so that it could be reported in line with the checklist and information was included to indicate that a protocol has been published, specific characteristics of the exclusion criteria were outlined, and a summary of the results was provided in relation to the research objectives.

### 2.1 Identifying the Research Questions

The research questions were developed to identify and examine what resources are available for people with intellectual disability and their parents/caregivers and recognise information gaps:

1. What online health literacy resources are available for people with intellectual disability and their families?
2. How do the domains of health literacy apply to the resources?
3. What formats are used to present information in the resources (e.g., Easy Read and pictorial)?

### 2.2 Identifying Relevant Resources

**FIGURE 1:**
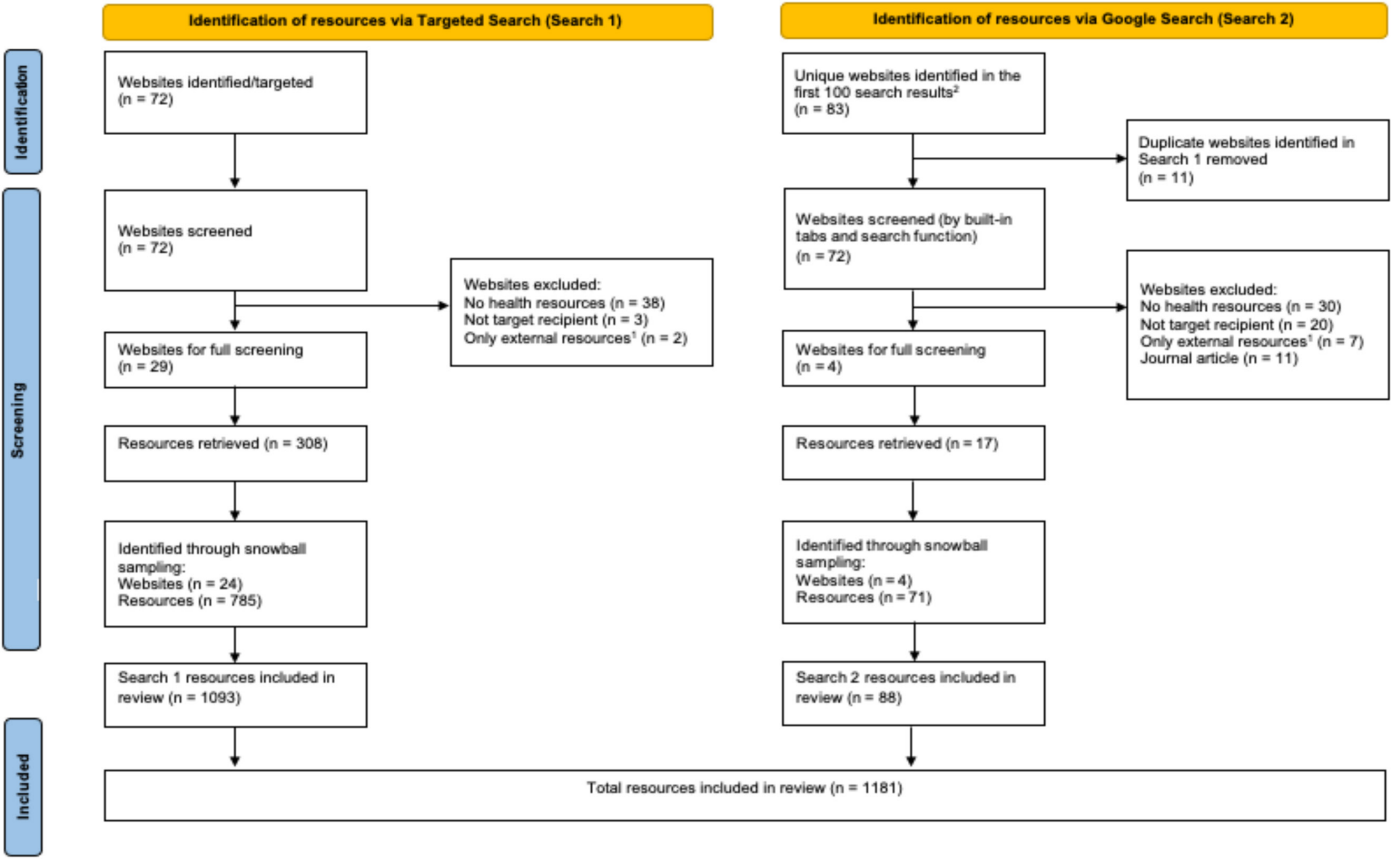
PRISMA flow chart.

There were two search strategies. First, a targeted search of organisation (government and non‐government) websites. Second, a search of key terms using Google. The process was guided by two grey literature scoping review protocols (Braithwaite et al. 2019; Brennan et al. 2023). For example, the decision to use a Google (in incognito mode) and a targeted search and to review a predetermined number of results was informed by the work of Braithwaite et al. (2019) and Brennan et al. (2023). Searching concluded on 31 October 2024. See Figure 1 for a PRISMA flowchart of the data collection process including the identified resources, the excluded resources (with example reasons), and the final sample for the two search strategies.

#### 2.2.1 Search 1

The research team developed a list of 72 Australian disability organisations. The organisations varied in type (government and non‐government), focus (disability generally, intellectual disability and diagnosis‐specific) and scope (local, state and national level). Organisation websites were scanned to identify content that met the inclusion criteria (outlined below). This was achieved by manually searching potentially relevant webpages and using website search functions where possible. Website searching was conducted by Z.Y. and a student intern independently. Snowball sampling occurred when links to external websites, including those from international organisations, were identified and reviewed for relevance. Snowball sampling was also conducted by Z. Y. and the student intern as well as T.N. and J.K. Initial searching was completed by Z.Y. and J.K. was available for in‐time queries when the application of the inclusion criteria was uncertain. Further, frequent team discussions were held during the search phase to ensure that the criteria and sample reflected the views of the team. Subsequent snowball sampling was conducted by Z.Y., T.N. and J.K. in parallel, where uncertainties could be discussed and clarified in real time.

#### 2.2.2 Search 2

An advanced Google search was conducted in incognito mode to avoid previous searches affecting the search results. See the protocol paper for more information (Yeap et al. 2024). The final search strategy is outlined in Table 1. The first 100 results were reviewed by (redacted) in close consultation with the research team. These results included international resources and did not specifically focus on Australian content. Duplicate websites were removed before the inclusion criteria were applied (same process as Search 1). Some of the resources identified in Search 2 came from the same websites, and as these were searched in their entirety, only unique websites were included. This search was undertaken by Z.Y. As above, J.K. and the team provided support resulting in a collaborative approach.

**TABLE 1:** Search strategies and key phrases used in Google advanced search.

Search terms and phrases (combine with AND)	
1	‘Intellectual disability’
2	health, **OR** ‘health literacy’, **OR** mental, **OR** physical, **OR** wellbeing

*Note:* Limited to the first 100 results available in English.

### 2.3 Resource Selection

#### 2.3.1 Inclusion and Exclusion Criteria

Resources were included if they contained content addressing physical or mental health and healthcare aimed at people with intellectual disability and/or their families that was freely available online. This includes other factors if they were directly linked to physical health, mental health, or healthcare (e.g., mental health supported by strong relationships). Resources were included from disability websites (not necessarily intellectual disability) if they included either Easy Read or plain English materials, as this suggests that they may be accessed by and accessible to people with intellectual disability. The following definitions of Easy Read and plain English from the Centre for Inclusive Design (2020) were used when classifying content. Easy Read texts were defined as those that use clear and basic language to form short sentences often presented as dot points with images to describe and explain information. Plain English texts use everyday language without jargon, in the form of short sentences and paragraphs. Training was conducted by the J.K. for the researchers conducting the search at the outset of coding to ensure that these definitions were clear and comprehensively understood to ensure compliance and consistency. Age, gender, health area and quality were not exclusion criteria. Resources were excluded if they were not presented in English and did not include a Google translate function. While Australian disability organisations were targeted in Search 1, resources from outside Australia were included if they were identified through snowball sampling. The reasons for excluding resources in the advanced Google search and the quantities of excluded resources are outlined in Figure 1. Some resources were excluded because they were aimed at healthcare providers and not people with intellectual disability. Academic papers were also excluded because they do not use accessible language and are not directed at people with intellectual disability and their families.

The research team defined tools as practical resources that aim to assist the person in home and healthcare settings by enhancing support (e.g., healthy living planning timetable), management (e.g., health tracking indicator), communication (e.g., communication books) or coping/engaging (e.g., social stories) health skills.

### 2.4 Collating, Summarising and Reporting the Results

Resources that met the inclusion criteria were compiled, and the following information was recorded: organisation details (name, government or non‐government, organisation location and URL), resource details [document or page title, URL for specific resource, presentation (e.g., text, pictures and Easy Read/plain English) and health area (e.g., physical health, mental health and sexual health)], targeted recipient (people with intellectual disability or family members, specified age group), health literacy domains addressed, and other characteristics such as whether references were provided, translation availability, and additional notes. The spreadsheet was populated by Z.Y., T.N., J.K., and the student intern and discussed within the research team. Where there were multiple relevant resources on a website, each resource was recorded as a separate entry.

Sørensen et al. (2012) health literacy domains were used to assess how health literacy was addressed in the resources. These include access (ability to find health information), understanding (ability to understand the content), appraise (ability to identify and reflect on important information and evaluate its quality) and apply (ability to use information and reflect on or rehearse content; Sørensen et al. 2012). The threshold for meeting the criterion of each health literacy domain was low to be inclusive, such that resources only had to minimally address the domain to satisfy conditions. See the protocol paper for information regarding the guiding questions used to evaluate how resources addressed the health literacy domains (Yeap et al. 2024).

## 3 Results

Research question 1 : What online health literacy resources are available for people with intellectual disability and their families?

**FIGURE 2:**
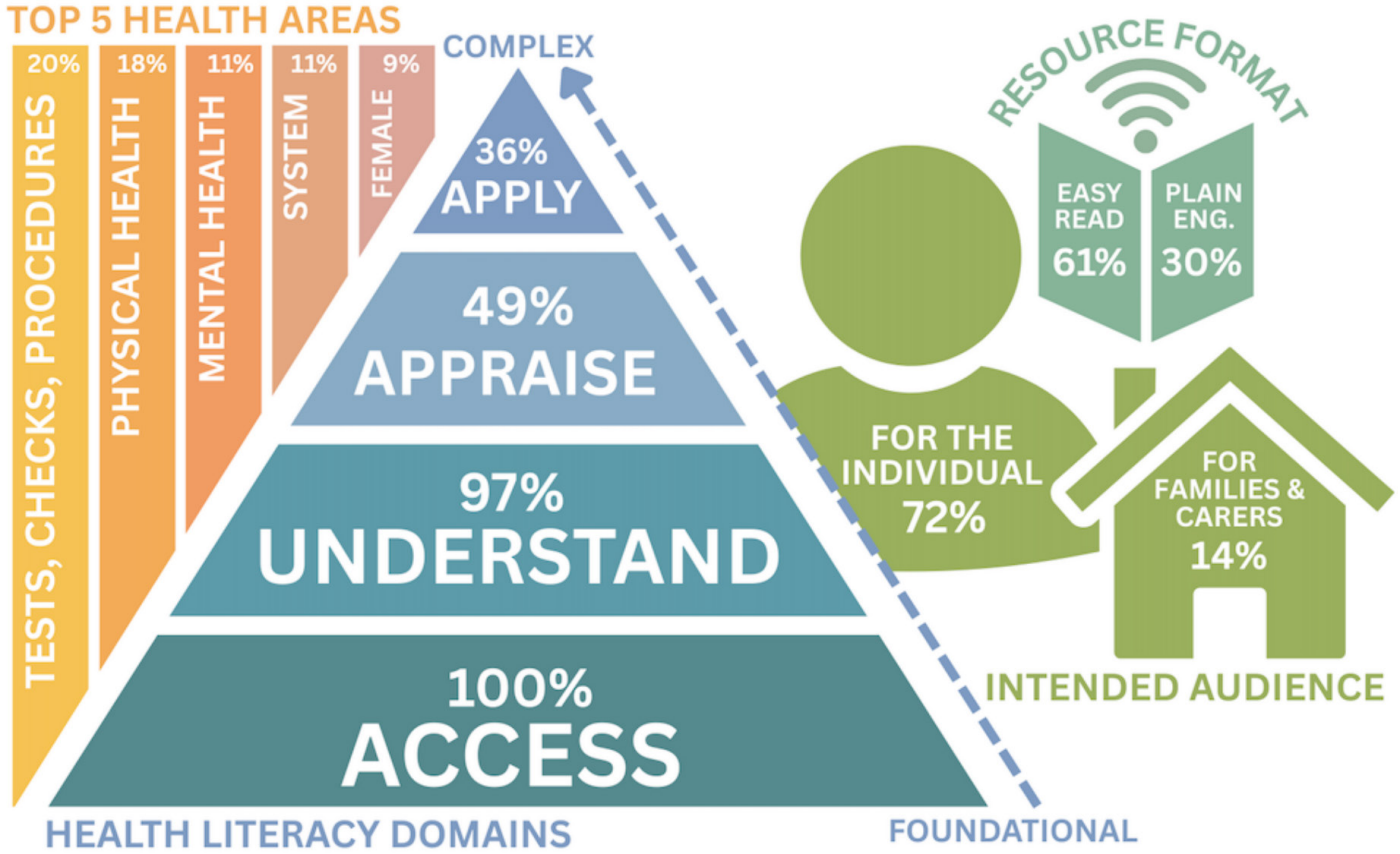
Visual summary of resource characteristics.

After initially scanning 172 websites across both searches, the final sample comprised 1165 resources from 60 websites. See Figure 2 for a visual representation of important findings. The most addressed area of health was tests, checks, and procedures (*n* = 227, 19.5%) followed by physical health (*n* = 213, 18.3%) and mental health (*n* = 135, 11.6%). Sexual health (*n* = 82, 7.0%) and men's health (*n* = 10, 0.9%) were covered the least, and most content came from nongovernmental organisations (*n* = 1138, 97.7%). Approximately a third of the resources came from Australia (*n* = 413, 35.5%) with most coming from the United Kingdom (*n* = 678, 58.2%). EasyHealth was the most cited organisation across the sample (*n* = 403, 34.6%; Figure 2).

Table 2 describes of the sample in terms of topic, formatting, intended audience and application of health literacy domains. Information was directed at people with intellectual disability (*n* = 837, 71. 8%) more than families and caregivers (*n* = 163, 14.0%). Most resources were aimed at adults or were not directed at a specific age group (*n* = 943, 80.9%). These groups are presented together because, while not specified, the topics and content suggest that adults are the most likely target audience. Almost a fifth (*n* = 198, 17.0%) of resources were directed at children and adolescents. The frequency of many of the health topics were similar for children and adolescents compared with those directed at adults and older adults (e.g., physical health, 16.2% vs. 18.7%, respectively); however, other topics such as mental health (5.6% vs. 12.8%, respectively) and sexual health (4.0% vs. 7.7%, respectively) were addressed less frequently. A chi squared test of independence was conducted to understand the relationship been age of intended audience (children and adolescents compared with adults and older adults) and resources covering mental health. This relationship was significant, *X* ^2^(1, *N* = 1165) = 8.47, *p* < 0.01 indicating substantially fewer mental health resources for young people. Another chi squared test of independence was completed for intended audience (children and adolescents compared to adults and older adults) and sexual health resource. While there were less resources this difference was not significant, *X* ^2^(1, *N* = 1165) = 3.28, *p* < 0.07.

**TABLE 2:** Description of resources and application of the health literacy domains.

Description of resources	*N* = 1165 (%)
Health area	
Tests, check and procedures	227 (19.5%)
Physical health	213 (18.3%)
Mental health	135 (11.6%)
Health Systems and services	129 (11.1%)
Female health	100 (8.6%)
Medication	96 (8.2%)
COVID‐19	88 (7.5%)
Illness, disease and condition	85 (7.3%)
Sexual health	82 (3.3%)
Men's health	10 (0.6%)
Presentation format	
Text and pictures	847 (72.7%)
Text only	216 (18.5%)
Text, pictures and video	26 (2.3%)
Video only	36 (1.3%)
Text and video	33 (2.8%)
Pictures only	7 (0.6%)
Language accessibility	
Easy Read	706 (60.6%)
Plain English	353 (30.3%)
Easy Read and plain English	102 (8.7%)
Not accessible (not sufficient language accessibility)	4 (0.3%)
Intended audience^a^	
People with intellectual disability	837 (71.8%)
Both person with intellectual disability and family/caregivers	165 (14.2%)
Family/caregivers	163 (14.0%)
Age‐specific	
None or adults	943 (80.9%)
Children and adolescents	198 (17.0%)
Older adults	24 (2.1%)
Health literacy domains^a^	
Access	1165 (100%)
Understand	1132 (97.2%)
Appraise	575 (49.4%)
Apply	415 (35.6%)

^a^ Fractions do not add up to 100% because resources could address multiple health literacy domains.

Table S1 includes the complete sample organised alphabetically by organisation to present the data and provide information on the resources available. However, the quality of these resources is not evaluated, meaning they may not present the best available, evidence‐based, accessible information and should therefore be used with caution.

Research question 2 : How do the domains of health literacy apply to the resources?

The application of the health literacy domains is presented in Table 2. All the resources met the criteria for access (*n* = 1165, 100%) and nearly all addressed understanding (*n* = 1132, 97.2%). The appraising (*n* = 575, 46.4%) and applying (*n* = 415, 35.6%) domains were less frequently addressed. A little over one quarter (28.7%, *n* = 119) of resources were healthcare tools and met the criteria for applying health information (Table 2).

Research question 3 : What formats are used to present information in the resources (e.g., Easy Read, pictorial)?

The format that resources were presented in is presented in Table 2. Content was most often presented using a combination of words and pictures (*n* = 847, 72.7%) followed by entirely text (*n* = 216, 18.5%). Content was generally presented in Easy Read (*n* = 706, 60.6%), plain English (*n* = 353, 30.3%), or both (*n* = 102, 8.8%). Very few resources had less accessible language, as indicated by the inclusion of complex terms without explanation (*n* = 4, 0.3%; Table 2).

## 4 Discussion

This review appears to be the first to systematically compile online health resources for people with intellectual disability and their families. A wide range of resources were found from a variety of sources that addressed multiple health areas and used different presentation styles. Tests, checks, and procedures and physical health were the most common health domains, and sexual health and men's health were the least addressed. Mental health and sexual health resources were limited for children and adolescents with intellectual disability. Most of the resources came from non‐government organisation websites from the United Kingdom and were most frequently from the EasyHealth website. Content was most often presented using pictures and words and in Easy Read. Though many resources addressed the health literacy domains of accessing and understanding health information, fewer resources addressed the more complex domains of appraising and using health information. These findings give insight into the free online health information landscape, including what is and what is not available for people with intellectual disability and their families. While Table S1 includes the resources identified in this study, it is not a curated resource, and information has not been assessed for quality.

The results of this study suggest gaps in the available suite of resources. Men's health was the area addressed the least in this sample. Males have a lower life expectancy than females worldwide, with the discrepancy ranging from 3.17 to 9.65 years depending on country of residence (Schöley et al. 2022). Research has found that men with mild to moderate intellectual disability can understand and act on health information, particularly with the support of health professionals who present information in a tailored way (Bollard 2017). This suggests that additional targeted online health resources for men may be needed.

Sexual health information was addressed less frequently than some other areas of health information. People with intellectual disability experience numerous barriers to accessing comprehensive and quality sexual health information and education (Matin et al. 2021; Michielsen and Brockschmidt 2021). Some factors that limit access to sexual education include perceptions of people with intellectual disability as asexual, rendering sexual education unnecessary, presenting information that is too general, and a lack of support for educators (Michielsen and Brockschmidt 2021). Research indicates this restricted access can result in limited sexual health knowledge (Matin et al. 2021). Additional online sexual health information may be beneficial and should be more readily available online.

Our search also found a lack of resources for Indigenous Australians and culturally and linguistically diverse groups with intellectual disability within the Australian setting. While we did not target specific groups with intellectual disability, these findings do suggest that more targeted and culturally secure health resources are needed to support culturally secure health care.

Mental health was the third most frequently identified health area in this sample, necessary because of the high prevalence of co‐occurring mental health problems in intellectual disability (Buckley et al. 2020). People with intellectual disability experience barriers to accessing mental health support (e.g., limited skilled workforce, lack of healthcare integration and collaboration; Whittle et al. 2018). Digital interventions (including online resources) have been identified as effective and affordable methods of treating mental health problems experienced by people with intellectual disability (Sheehan and Hassiotis 2017). However, further research regarding efficacy and ways to manage potential safety issues is needed (Sheehan and Hassiotis 2017).

All the resources met the minimum criteria for accessibility and understanding by presenting information in Easy Read or plain English. The number of Easy Read or plain English materials is unsurprising as this was an inclusion criterion for webpages that were not intellectual disability specific. However, Easy Read or plain English information presentation is important because, while literacy levels vary for people with intellectual disability, Easy Read health information has been identified as a preferred format for presenting health information for people with intellectual disability (Doherty et al. 2020). However, some research finds that using simplified language does not necessarily improve understanding of health information more than unmodified content and suggests that, to be accessible, information should be tailored to the individual (Chinn and Homeyard 2017). The Centre for Disease Control and Prevention (2023) has created a tool and evidence‐based resource with recommendations for developing resources for people with intellectual and developmental disability and very low literacy (Squiers et al. 2023).

The more complex health literacy domains (i.e., appraise and apply) were addressed less frequently than the less complex ones (i.e., access and understand). Appraising health information is partially about evaluating evidence for the information. Few resources included references for their content, limiting the capacity of readers to critically evaluate the information. While information sources may not be important to all readers, these details would improve opportunities for the critical evaluation of information quality. Applying health information can be the most complex form of health literacy, but it is the end goal. The sample included some tools designed to assist people with intellectual disability and their families to apply the information in healthcare settings (e.g., ‘Nourish’ a co‐designed gastrostomy education resource based on Social Cognitive Theory; Murphy et al. 2023). Further research is needed to evaluate the efficacy of these tools, identify techniques that are effective, and increase their application to other resources.

The next step in this project is to use the information presented in Table S1 to address the identified gaps and develop or modify some available resources to fill health literacy topic gaps for people with intellectual disability and their families. We will also consider available tools to evaluate the quality of online resources for people with intellectual disability. Importantly, we will also consider the information needs of people with intellectual disability and their supporters with very low to no literacy.

### 4.1 Strengths and Limitations

This study appears to be the first to synthesise online health resources. As such, this study and the accompanying protocol paper present a methodological example for future research that seeks to understand the types of information available online. A limitation of this study is that the sample does not represent all online health information resources. Our search was extensive; however, we recognise that an online search to identify every health resource globally would be impossible. Our results represent a large and comprehensive sample of online health information available to people with intellectual disability and their families and can suggest patterns of available information and avenues for resource development such as for mental and sexual health for children and adolescents. For example, we identify gaps that can inform future resource funding and development. Further, our search terms were broad and excluded specific conditions, groups, and varying use of terminology and labels (e.g., learning disability is often used in the United Kingdom). Additionally, this study did not evaluate the quality of the resources beyond the application of the health literacy domains. Future research should examine, develop, and apply a criterion to assess the quality of online health resources for people with intellectual disability and their families. Additionally, this study did not classify resources in terms of how they conform to Web Content Accessibility Guidelines (WCAG) 2.1 (2025) or included audio description or screen reader compatibility. Including this information would have enhanced the analysis of information accessibility. Further, inclusion and exclusion criteria training was provided and reliability was evaluated pragmatically with ongoing checking and collaboration including agreeance on classification of sample resources. However, reliability was not evaluated statistically.

## 5 Conclusions

Improving health literacy is an integral part of addressing the health disparities experienced by people with intellectual disability. People with intellectual disability look to the internet for health information but experience numerous barriers. This study identified a potential lack of information in areas such as mental health for children and adolescents and sexual and men's health and suggests more resources are needed in these areas. Most of the identified resources used accessible language; however, some research suggests the need for individualised information. We suggest that new resources include evidence for the health information presented and that health application tools should be developed and evaluated to account for individual needs.

## Ethics Statement

Ethical approval was not necessary as no new data were collected.

## Conflicts of Interest

The authors declare no conflicts of interest.

## Supporting information


**Table S1:** ‘Health resources for people with intellectual disability identified in this study’.

## Data Availability

All data are publicly available.

## References

[jir70028-bib-0001] Alfredsson Ågren, K. , A. Kjellberg , and H. Hemmingsson . 2020. “Internet Opportunities and Risks for Adolescents With Intellectual Disabilities: A Comparative Study of Parents' Perceptions.” Scandinavian Journal of Occupational Therapy 27, no. 8: 601–613. 32538241 10.1080/11038128.2020.1770330

[jir70028-bib-0002] Alshammari, M. , O. Doody , and I. Richardson . 2018. “Barriers to the Access and Use of Health Information by Individuals With Intellectual and Developmental Disability IDD: A Review of the Literature.” In 2018 IEEE International Conference on Healthcare Informatics (ICHI), 294–298. IEEE. 10.1109/ICHI.2018.00040.

[jir70028-bib-0003] Arksey, H. , and L. O'Malley . 2005. “Scoping Studies: Towards a Methodological Framework.” International Journal of Social Research Methodology 8, no. 1: 19–32. 10.1080/1364557032000119616.

[jir70028-bib-0004] Australian Government . 2021. “National Roadmap for Improving the Health of People With Intellectual Disability.” https://www.health.gov.au/sites/default/files/documents/2021/08/national‐roadmap‐for‐improving‐the‐health‐of‐people‐with‐intellectual‐disability.pdf.

[jir70028-bib-0005] Balogh, R. , M. Brownell , H. Ouellette‐Kuntz , and A. Colantonio . 2010. “Hospitalisation Rates for Ambulatory Care Sensitive Conditions for Persons With and Without an Intellectual Disability‐A Population Perspective.” Journal of Intellectual Disability Research 54, no. 9: 820–832. 10.1111/j.1365-2788.2010.01311.x. 20704636

[jir70028-bib-0006] Berkman, N. D. , S. L. Sheridan , K. E. Donahue , D. J. Halpern , and K. Crotty . 2011. “Low Health Literacy and Health Outcomes: An Updated Systematic Review.” Annals of Internal Medicine 155, no. 2: 97–107. 10.7326/0003-4819-155-2-201107190-00005. 21768583

[jir70028-bib-0007] Bollard, M. 2017. “Health Promotion and Intellectual Disability: Listening to Men.” Health & Social Care in the Community 25, no. 1: 185–193. 10.1111/hsc.12291. 26434374

[jir70028-bib-0008] Braithwaite, J. , Y. Zurynski , K. Ludlow , J. Holt , H. Augustsson , and M. Campbell . 2019. “Towards Sustainable Healthcare System Performance in the 21st Century in High‐Income Countries: A Protocol for a Systematic Review of the Grey Literature.” BMJ Open 9, no. 1: e025892. 10.1136/bmjopen-2018-025892. PMC6340467 30782754

[jir70028-bib-0009] Brennan, L. , L. Brewster , J. Lunn , et al. 2023. “How Do Hospitals Address Health Inequalities Experienced by Children and Young People: A Grey Literature Scoping Review Protocol.” BMJ Open 13, no. 4: e071682. 10.1136/bmjopen-2023-071682 PMC10151951 37105699

[jir70028-bib-0010] Buckley, N. , E. J. Glasson , W. Chen , et al. 2020. “Prevalence Estimates of Mental Health Problems in Children and Adolescents With Intellectual Disability: A Systematic Review and Meta‐Analysis.” Australian and New Zealand Journal of Psychiatry 54, no. 10: 970–984. 10.1177/0004867420924101. 32475125

[jir70028-bib-0011] Centers for Disease Control and Prevention (U.S.) . 2023. “How to Develop Products for Adults With Intellectual Developmental Disabilities and Extreme Low Literacy: A Product Development Tool.” https://www.cdc.gov/ccindex/pdf/idd‐ell‐product‐development‐tool‐508.pdf.

[jir70028-bib-0012] Centre for Inclusive Design . 2020. “A Guide to Creating Accessible Content.” https://centreforinclusivedesign.org.au/wp‐content/uploads/2020/04/Easy‐English‐vs‐Plain‐English_accessible.pdf.

[jir70028-bib-0013] Chadwick, D. , C. Wesson , and C. Fullwood . 2013. “Internet Access by People With Intellectual Disabilities: Inequalities and Opportunities.” Future Internet 5, no. 3: 376–397.

[jir70028-bib-0014] Chinn, D. 2014. “Critical Health Literacy Health Promotion and People With Intellectual Disabilities.” Asia‐Pacific Journal of Health, Sport and Physical Education 5, no. 3: 249–265. 10.1080/18377122.2014.940811.

[jir70028-bib-0015] Chinn, D. , and C. Homeyard . 2017. “Easy Read and Accessible Information for People With Intellectual Disabilities: Is it Worth It? A Meta‐Narrative Literature Review.” Health Expectations 20, no. 6: 1189–1200. 10.1111/hex.12520. 27862757 PMC5689240

[jir70028-bib-0016] Doherty, A. J. , H. Atherton , P. Boland , et al. 2020. “Barriers and Facilitators to Primary Health Care for People With Intellectual Disabilities and/or Autism: An Integrative Review.” BJGP Open 4, no. 3: 1–10. 10.3399/bjgpopen20X101030. PMC7465578 32605913

[jir70028-bib-0017] Dunn, K. , L. Hughes‐McCormack , and S. A. Cooper . 2018. “Hospital Admissions for Physical Health Conditions for People With Intellectual Disabilities: Systematic Review.” Journal of Applied Research in Intellectual Disabilities 31, no. 1: 1–10. 10.1111/jar.12360. 28467010

[jir70028-bib-0018] Emerson, E. 2021. “Inequalities and Inequities in the Health of People With Intellectual Disabilities.” In Oxford Research Encyclopedia of Global Public Health. Oxford University Press. 10.1093/acrefore/9780190632366.013.326.

[jir70028-bib-0019] Geukes, C. , D. Bruland , and Ä.‐D. Latteck . 2018. “Health Literacy in People With Intellectual Disabilities: A Mixed‐Method Literature Review.” Kontakt 20, no. 4: e416–e423.

[jir70028-bib-0020] Hughes‐McCormack, L. A. , E. Rydzewska , A. Henderson , C. MacIntyre , J. Rintoul , and S. A. Cooper . 2017. “Prevalence of Mental Health Conditions and Relationship With General Health in a Whole‐Country Population of People With Intellectual Disabilities Compared With the General Population.” BJPsych Open 3, no. 5: 243–248. 10.1192/bjpo.bp.117.005462. 29034100 PMC5620469

[jir70028-bib-0021] Jia, X. , Y. Pang , and L. S. Liu . 2021. “Online Health Information Seeking Behavior: A Systematic Review.” Healthcare 9, no. 12: 1740. 10.3390/healthcare9121740. 34946466 PMC8701665

[jir70028-bib-0022] Kuruppu Arachchi, T. , L. Sitbon , J. Zhang , S. Koplick , M. Hoogstrate , and M. Brereton . 2023. “Web Search to Access Health Information by Adults With Intellectual Disability.” Online Information Review 47, no. 6: 1098–1115. 10.1108/OIR-06-2021-0337.

[jir70028-bib-0023] Liao, P. , C. Vajdic , J. Trollor , and S. Reppermund . 2021. “Prevalence and Incidence of Physical Health Conditions in People With Intellectual Disability ‐ A Systematic Review.” PLoS ONE 16, no. 8: e0256294. 10.1371/journal.pone.0256294. 34428249 PMC8384165

[jir70028-bib-0024] Magnani, J. W. , M. S. Mujahid , H. D. Aronow , et al. 2018. “Health Literacy and Cardiovascular Disease: Fundamental Relevance to Primary and Secondary Prevention: A Scientific Statement From the American Heart Association.” Circulation 138, no. 2: e48–e74. 29866648 10.1161/CIR.0000000000000579 PMC6380187

[jir70028-bib-0025] Matin, B. K. , M. Ballan , F. Darabi , A. K. Karyani , M. Soofi , and S. Soltani . 2021. “Sexual Health Concerns in Women With Intellectual Disabilities: A Systematic Review in Qualitative Studies.” BMC Public Health 21: 1–21. 10.1186/s12889-021-12027-6. 34717594 PMC8556840

[jir70028-bib-0026] Michielsen, K. , and L. Brockschmidt . 2021. “Barriers to Sexuality Education for Children and Young People With Disabilities in the WHO European Region: A Scoping Review.” Sex Education 21, no. 6: 674–692. 10.1080/14681811.2020.1851181.

[jir70028-bib-0027] Murphy, N. , M. Ravikumara , M. Butterworth , et al. 2023. “A Co‐Designed Online Education Resource on Gastrostomy Feeding for Parents and Caregivers to Support Clinical Care.” Journal of Pediatric Gastroenterology and Nutrition 77, no. 5: 672–678. 10.1097/MPG.0000000000003925. 37612813

[jir70028-bib-0028] Paez, A. 2017. “Gray Literature: An Important Resource in Systematic Reviews.” Journal of Evidence‐Based Medicine 10, no. 3: 233–240. 28857505 10.1111/jebm.12266

[jir70028-bib-0030] Schöley, J. , J. M. Aburto , I. Kashnitsky , et al. 2022. “Life Expectancy Changes Since COVID‐19.” Nature Human Behaviour 6, no. 12: 1649–1659. 10.1038/s41562-022-01450-3. PMC9755047 36253520

[jir70028-bib-0031] Shahid, R. , M. Shoker , L. M. Chu , R. Frehlick , H. Ward , and P. Pahwa . 2022. “Impact of Low Health Literacy on Patients' Health Outcomes: A Multicenter Cohort Study.” BMC Health Services Research 22, no. 1: 1148. 10.1186/s12913-022-08527-9. 36096793 PMC9465902

[jir70028-bib-0032] Sheehan, R. , and A. Hassiotis . 2017. “Digital Mental Health and Intellectual Disabilities: State of the Evidence and Future Directions.” BMJ Mental Health 20, no. 4: 107–111. 10.1136/eb-2017-102759. PMC10516400 28947677

[jir70028-bib-0033] Sørensen, K. , S. Van den Broucke , J. Fullam , et al. 2012. “Health Literacy and Public Health: A Systematic Review and Integration of Definitions and Models.” BMC Public Health 12: 80. 10.1186/1471-2458-12-80. 22276600 PMC3292515

[jir70028-bib-0034] Squiers, L. , M. M. Lynch , S. L. Holt , et al. 2023. “Building Evidence for Principles to Guide the Development of Products for Adults WITH Intellectual and Developmental Disabilities and Extreme Low Literacy—A Product Development Tool.” Healthcare (Basel) 11, no. 12: 1742. 10.3390/healthcare11121742. 37372860 PMC10298040

[jir70028-bib-0035] Tricco, A. C. , E. Lillie , W. Zarin , et al. 2018. “PRISMA Extension for Scoping Reviews (PRISMA‐ScR): Checklist and Explanation.” Annals of Internal Medicine 169, no. 7: 467–473. 10.7326/m18-0850. 30178033

[jir70028-bib-0036] Tyrer, F. , R. Morriss , R. Kiani , S. K. Gangadharan , and M. J. Rutherford . 2022. “Mortality Disparities and Deprivation Among People With Intellectual Disabilities in England: 2000–2019.” Journal of Epidemiology and Community Health 76, no. 2: 168–174. 10.1136/jech-2021-216798. 34244310

[jir70028-bib-0037] United Nations . 2006. “The Convention on the Rights of Persons With Disabilites.” https://social.desa.un.org/issues/disability/crpd/convention‐on‐the‐rights‐of‐persons‐with‐disabilities‐articles. 10.1515/9783110208856.203 18348362

[jir70028-bib-0029] Web Content Accessibility Guidelines 2.1 . 2025. “W3C World Wide Web Consortium Recommendation.” https://www.w3.org/TR/YYYY/REC‐WCAG21‐YYYYMMDD/, latest version at https://www.w3.org/TR/WCAG21/.

[jir70028-bib-0038] Weise, J. C. , P. Srasuebkul , and J. N. Trollor . 2021. “Potentially Preventable Hospitalisations of People With Intellectual Disability in New South Wales.” Medical Journal of Australia 215, no. 1: 31–36. 10.5694/mja2.51088. 34028026

[jir70028-bib-0039] Whittle, E. L. , K. R. Fisher , S. Reppermund , R. Lenroot , and J. Trollor . 2018. “Barriers and Enablers to Accessing Mental Health Services for People With Intellectual Disability: A Scoping Review.” Journal of Mental Health Research in Intellectual and Developmental Disabilities 11, no. 1: 69–102. 10.1080/19315864.2017.1408724.

[jir70028-bib-0040] Wolf, M. S. , T. C. Davis , L. M. Curtis , et al. 2016. “A Patient‐Centered Prescription Drug Label to Promote Appropriate Medication Use and Adherence.” Journal of General Internal Medicine 31: 1482–1489. 27542666 10.1007/s11606-016-3816-x PMC5130952

[jir70028-bib-0041] Yeap, Z. , J. Keeley , R. Skoss , et al. 2024. “Online Health Literacy Resources for People with Intellectual Disability: Protocol for a Grey Literature Scoping Review.” BMJ Open 14, no. 11: e088509. 10.1136/bmjopen-2024-088509 PMC11590844 39581735

