# Supplementary material for: Online Health Literacy Resources for People With Intellectual Disability: A Grey Literature Scoping Review
Source: J Intellect Disabil Res. 2025 Aug 2;70(2):121–9. doi: 10.1111/jir.70028 (PMC12757201; doi:10.1111/jir.70028)
Supplement: Supplementary file 1 — Table S1: ‘Health resources for people with intellectual disability identified in this study’. [file JIR-70-121-s001.docx]

| **Organisation** | **Organisation URL** | **Webpage Title** | **Page URL** |
| --- | --- | --- | --- |
| Ability WA | <https://www.abilitywa.com.au/> | Emergency & COVID-19 Resources | [COVID-19 Resources (abilitywa.com.au)](https://www.abilitywa.com.au/information-hub/covid19-resources/) |
|  |  | Be Ready: Emergency and Disaster Planning Tool | [be-ready-tool.pdf (abilitywa.com.au)](https://www.abilitywa.com.au/media/3041/be-ready-tool.pdf) |
| Academic Autistic Spectrum Partnership in Research and Education (AASPIRE) | [https://autismandhealth.org](https://autismandhealth.org/) | Staying Healthy | [For Patients & Supporters of Autistic Adults (autismandhealth.org)](https://autismandhealth.org/?a=pt&p=main&theme=ltlc&size=small&t=pt_hth) |
|  |  | Excerise | [For Autistic Adults: Exercise (autismandhealth.org)](https://autismandhealth.org/?a=pt&p=detail&t=pt_hth&theme=ltlc&size=small&s=hth_exer) |
|  |  | Nutriton | [For Autistic Adults: Nutrition (autismandhealth.org)](https://autismandhealth.org/?a=pt&p=detail&t=pt_hth&theme=ltlc&size=small&s=hth_nutr) |
|  |  | Preventative Health | [For Autistic Adults: Preventative Care (autismandhealth.org)](https://autismandhealth.org/?a=pt&p=detail&t=pt_hth&theme=ltlc&size=small&s=hth_prev) |
|  |  | Forms & Worksheets | [For Patients & Supporters of Autistic Adults (autismandhealth.org)](https://autismandhealth.org/?a=pt&p=main&theme=ltlc&size=small&t=pt_frm) |
|  |  | Healthcare | [For Patients & Supporters of Autistic Adults (autismandhealth.org)](https://autismandhealth.org/?a=pt&p=main&theme=ltlc&size=small&t=pt_hc) |
|  |  | Your Rights in Healthcare | [For Patients & Supporters of Autistic Adults (autismandhealth.org)](https://autismandhealth.org/?a=pt&p=main&theme=ltlc&size=small&t=pt_rts) |
| Amaze | <https://www.amaze.org.au/> | Puberty and Autism | [Information Sheet 27 - Puberty and autism.pub (amaze.org.au)](https://www.amaze.org.au/wp-content/uploads/2019/08/Amaze-Information-Sheet-Puberty-and-autism.pdf) |
|  |  | Sleep problems in autistic children | [Microsoft Word - Information sheet 9 - Sleep problems in autistic children (2) (amaze.org.au)](https://www.amaze.org.au/wp-content/uploads/2019/08/Amaze-Information-sheet-Sleep-problems-in-autistic-children.pdf) |
|  |  | Going to the GP about my mental health – A guide for Autistic women | [Going to the GP about my mental health - A guide for Autistic women - (amaze.org.au)](https://www.amaze.org.au/guide-for-autistic-women/) |
|  |  | Medicare benefits for people under 25 years (PDF) | <https://www.amaze.org.au/wp-content/uploads/2023/05/Medicare-benefits-for-children-and-young-people.pdf> |
|  |  | Medicare benefits for adults 25 years and over (PDF) | <https://www.amaze.org.au/wp-content/uploads/2023/05/Medicare-benefits-provided-for-adults.pdf> |
|  |  | Using Tampons | [Using-Tampons-Social-Script_FINAL2023.pdf (amaze.org.au)](https://www.amaze.org.au/wp-content/uploads/2021/05/Using-Tampons-Social-Script_FINAL2023.pdf) |
|  |  | Using a Pad | [Using-a-Pad-Social-Script-February-2019_RS-FINAL_2023-updated.pdf (amaze.org.au)](https://www.amaze.org.au/wp-content/uploads/2021/05/Using-a-Pad-Social-Script-February-2019_RS-FINAL_2023-updated.pdf) |
| Australian Commission on Safety and Quality in Healthcare (ACSQHC) | <https://www.safetyandquality.gov.au/> | About healthcare rights for people with cognitive impairment | [About healthcare rights for people with cognitive impairment (safetyandquality.gov.au)](https://www.safetyandquality.gov.au/sites/default/files/2020-06/20021_about_healthcare_rights_for_people_with_cognitive_impairment_final_draft_0.pdf) |
|  |  | About me: Your health care is about you | [About Me: Questions to ask about Reasonable Adjustment - Easy Read Fact Sheet (safetyandquality.gov.au)](https://www.safetyandquality.gov.au/sites/default/files/2023-03/about_me_questions_to_ask_about_reasonable_adjustment_-_easy_read_fact_sheet.pdf) |
| Australian Government Department of Health and Aged Care | [https://www.health.gov.au](https://www.health.gov.au/) | COVID-19 vaccination - Easy Read resources | <https://www.health.gov.au/resources/collections/covid-19-vaccination-easy-read-resources> |
| Autism Association of WA (AAWA) | <https://www.autism.org.au/> | Autism and Mental Health Resource | [Autism and Mental Health Resource - Autism Association of Western Australia](https://www.autism.org.au/accessing-mental-health-and-healthcare-services/autism-and-mental-health/autism-and-mental-health-resource/) |
|  |  | Accessing Help for Mental Health Concerns | [Accessing Help for Mental Health Concerns - Autism Association of Western Australia](https://www.autism.org.au/accessing-mental-health-and-healthcare-services/autism-and-mental-health/accessing-help-for-mental-health-concerns/) |
|  |  | Accessing Mental Health Supports | [AccessingMentalHealthSupports_0822.pdf (juiceboxcreative-autismwa.s3.ap-southeast-2.amazonaws.com)](https://juiceboxcreative-autismwa.s3.ap-southeast-2.amazonaws.com/wp-content/uploads/2022/03/15150017/AccessingMentalHealthSupports_0822.pdf) |
|  |  | Recommended Books & Websites | [Recommended Books & Websites - Autism Association of Western Australia](https://www.autism.org.au/accessing-mental-health-and-healthcare-services/autism-and-mental-health/recommended-books-websites/) |
|  |  | Community Inclusion & Support | [Community Inclusion & Support - Autism Association of Western Australia](https://www.autism.org.au/accessing-mental-health-and-healthcare-services/autism-and-mental-health/community-inclusion-support/) |
|  |  | COVID-19 | [COVID-19 - Autism Association of Western Australia](https://www.autism.org.au/resource-library/coronavirus-covid-19-update/) |
|  |  | Accessing Dental and Healthcare Services | [Accessing Dental and Healthcare Services - Autism Association of Western Australia](https://www.autism.org.au/resource-library/accessing-dental-and-healthcare-services/) |
|  |  | Dental Filling Social Story | https://juiceboxcreative-autismwa.s3.ap-southeast-2.amazonaws.com/wp-content/uploads/2020/05/13113835/Dental-filling-social-story.pdf |
|  |  | Dental Needle Social Story | https://juiceboxcreative-autismwa.s3.ap-southeast-2.amazonaws.com/wp-content/uploads/2020/05/13113838/Dental-needle-social-story.pdf |
|  |  | Dental Visit Social Story | https://juiceboxcreative-autismwa.s3.ap-southeast-2.amazonaws.com/wp-content/uploads/2020/05/13113841/Dentist-visit-social-story.pdf |
|  |  | Tooth Extraction Social Story | https://juiceboxcreative-autismwa.s3.ap-southeast-2.amazonaws.com/wp-content/uploads/2020/05/13113918/Tooth-extraction-social-story.pdf |
|  |  | Dental Information Card | https://juiceboxcreative-autismwa.s3.ap-southeast-2.amazonaws.com/wp-content/uploads/2021/04/27084928/Dental-Information-card.pdf |
|  |  | Dental Visuals | https://juiceboxcreative-autismwa.s3.ap-southeast-2.amazonaws.com/wp-content/uploads/2021/04/27084932/Dentist-Visuals-updated.pdf |
|  |  | CT Scan Social Story | https://juiceboxcreative-autismwa.s3.ap-southeast-2.amazonaws.com/wp-content/uploads/2016/11/21151634/CT-scan-social-story-1.pdf |
|  |  | Getting an ECG Social Story | https://juiceboxcreative-autismwa.s3.ap-southeast-2.amazonaws.com/wp-content/uploads/2020/05/13113845/ECG-social-story-1.pdf |
|  |  | Getting a Cast Social Story | https://juiceboxcreative-autismwa.s3.ap-southeast-2.amazonaws.com/wp-content/uploads/2020/05/13113908/Plastering-social-story-1.pdf |
|  |  | Getting An Injection Social Story | https://juiceboxcreative-autismwa.s3.ap-southeast-2.amazonaws.com/wp-content/uploads/2020/05/13113853/Getting-an-Injection-Social-story-1.pdf |
|  |  | Having an Operation Social Story | https://juiceboxcreative-autismwa.s3.ap-southeast-2.amazonaws.com/wp-content/uploads/2020/05/13113905/Operations-Social-story-1.pdf |
|  |  | IV Access Social Story | https://juiceboxcreative-autismwa.s3.ap-southeast-2.amazonaws.com/wp-content/uploads/2020/05/13113857/IV-Access-social-story-1.pdf |
|  |  | MRI Social Story | https://juiceboxcreative-autismwa.s3.ap-southeast-2.amazonaws.com/wp-content/uploads/2020/05/13113901/MRI-social-story-1.pdf |
|  |  | Taking Medication Social Story | https://juiceboxcreative-autismwa.s3.ap-southeast-2.amazonaws.com/wp-content/uploads/2020/05/13113912/Taking-Medication-social-story-1.pdf |
|  |  | Tonsillectomy Social Story | https://juiceboxcreative-autismwa.s3.ap-southeast-2.amazonaws.com/wp-content/uploads/2020/05/13113915/Tonsillectomy-Social-story-1.pdf |
|  |  | Ultrasound Social Story | https://juiceboxcreative-autismwa.s3.ap-southeast-2.amazonaws.com/wp-content/uploads/2016/11/21151726/Ultrasound-social-story-1.pdf |
|  |  | Medical Information card | https://juiceboxcreative-autismwa.s3.ap-southeast-2.amazonaws.com/wp-content/uploads/2019/03/01132709/Medical-Information-card.pdf |
|  |  | The Cool Report MRI | https://juiceboxcreative-autismwa.s3.ap-southeast-2.amazonaws.com/wp-content/uploads/2019/03/01132705/The-Cool-Report-MRI.pdf |
|  |  | Health Visuals | https://juiceboxcreative-autismwa.s3.ap-southeast-2.amazonaws.com/wp-content/uploads/2021/04/28130756/Health-Visuals.pdf |
|  |  | Blood Test Social Story | https://juiceboxcreative-autismwa.s3.ap-southeast-2.amazonaws.com/wp-content/uploads/2016/11/21151622/Blood-Tests-social-story-1.pdf |
| Black Dog Institute | <https://www.blackdoginstitute.org.au/> | Healthy Mind - Recognising Feelings | [healthymind.org.au/Topic.aspx?AreaID=1#](https://www.healthymind.org.au/Topic.aspx?AreaID=1) |
|  |  | Breathe and relax | [healthymind.org.au/Topic.aspx?AreaID=2#](https://www.healthymind.org.au/Topic.aspx?AreaID=2) |
|  |  | Taming anger | [healthymind.org.au/Topic.aspx?AreaID=3#](https://www.healthymind.org.au/Topic.aspx?AreaID=3) |
|  |  | Having more fun | [https://www.healthymind.org.au/Topic.aspx?AreaID=4#](https://www.healthymind.org.au/Topic.aspx?AreaID=4) |
|  |  | Tackling unhelpful thinking | [healthymind.org.au/Topic.aspx?AreaID=5#](https://www.healthymind.org.au/Topic.aspx?AreaID=5) |
|  |  | Omega-3 and mood disorders | [Omega-3-and-mood-disorders-fact-sheet.pdf (blackdoginstitute.org.au)](https://www.blackdoginstitute.org.au/wp-content/uploads/2022/08/Omega-3-and-mood-disorders-fact-sheet.pdf?sfvrsn=4) |
| Books Beyond Words | [booksbeyondwords.co.uk](https://booksbeyondwords.co.uk/) | Health | [Free downloads — Beyond Words (booksbeyondwords.co.uk)](https://booksbeyondwords.co.uk/resources-dl/#health) |
| Carer Gateway | <https://www.carergateway.gov.au/> | Managing health and behaviour | [Managing health and behaviour \| Carer Gateway](https://www.carergateway.gov.au/managing-health-behaviour) |
|  |  | Working with health services | [Working with health services \| Carer Gateway](https://www.carergateway.gov.au/working-health-services) |
| Centers for Disease Control and Prevention | <https://www.cdc.gov/> | Disability and Health Information for Women with Disabilities | [Disability and Health Information for Women with Disabilities \| CDC](https://www.cdc.gov/ncbddd/disabilityandhealth/women.html) |
|  |  | [Breast Cancer Screening — The Right to Know Campaign](https://www.cdc.gov/right-to-know/) | [Four Women Tell Their Stories \| Breast Cancer Screening — The Right to Know Campaign \| CDC](https://www.cdc.gov/right-to-know/php/campaign-home/?CDC_AAref_Val=https://www.cdc.gov/ncbddd/disabilityandhealth/righttoknow/) |
|  |  | Breast Cancer Screening Test | <https://www.cdc.gov/right-to-know/media/pdfs/TipsheetsENG.pdf> |
|  |  | Breast Cancer Basics | [Breast Cancer Basics \| Breast Cancer \| CDC](https://www.cdc.gov/breast-cancer/about/?CDC_AAref_Val=https://www.cdc.gov/cancer/breast/basic_info/index.htm) |
|  |  | Disability and Healthy Living | [Disability and Health Healthy Living \| CDC](https://www.cdc.gov/ncbddd/disabilityandhealth/healthyliving.html) |
|  |  | Tips for Communicating with Female Patients with Intellectual Disabilities | https://www.cdc.gov/ncbddd/disabilityandhealth/materials/communicating-with-female-patients.html |
|  |  | Disability and Health Related Conditions | [Disability and Health Related Conditions \| CDC](https://www.cdc.gov/ncbddd/disabilityandhealth/relatedconditions.html) |
|  |  | About Spina Bifida | <https://www.cdc.gov/spina-bifida/about/index.html> |
|  |  | Living with Spina Bifida | https://www.cdc.gov/spina-bifida/living-with/index.html |
|  |  | Manage Spina Bifida | https://www.cdc.gov/spina-bifida/treatment/index.html |
|  |  | Multiple Sclerosis | https://www.ninds.nih.gov/health-information/disorders/multiple-sclerosis |
|  |  | Fatigue | https://medlineplus.gov/ency/article/003088.htm |
|  |  | Disability and Obesity | https://www.cdc.gov/ncbddd/disabilityandhealth/obesity.html |
| Centre for Developmental Disability Health, Monash Health | <https://cddh.monashhealth.org/> | Accessing mental health services for people with an intellectual disability | [accessing-mental-health-services-for-people-with-id-2015.pdf (monashhealth.org)](https://cddh.monashhealth.org/wp-content/uploads/2016/11/accessing-mental-health-services-for-people-with-id-2015.pdf) |
|  |  | A quick reference guide to hospital care for people with a disability | [MOIRA Cover FINAL REV2004 (monashhealth.org)](https://cddh.monashhealth.org/wp-content/uploads/2017/01/hospital-care.pdf) |
|  |  | Pap tests: the plain facts | [pap-tests-the-plain-facts.pdf (monashhealth.org)](https://cddh.monashhealth.org/wp-content/uploads/2016/11/pap-tests-the-plain-facts.pdf) |
| CHW School-Link | <http://www.schoollink.chw.edu.au/> | Children and young people with learning disabilities - understanding their mental health | [Children-Young-People-with-Learning-Disabilities.pdf (sqspcdn.com)](https://static1.1.sqspcdn.com/static/f/551166/27603088/1498202800267/Children-Young-People-with-Learning-Disabilities.pdf?token=H38ZH%2BM9Ucy2OEpvzmOS3hxQUvw%3D) |
| Consumers Health Forum of Australia | <https://chf.org.au/be-health-aware> | Being Well | <https://chf.org.au/be-health-aware#wellbeing> |
|  |  | What to ask your doctor | <https://chf.org.au/be-health-aware#health1> |
| Council for Intellectual Disability | <https://cid.org.au/> | How to cope in a heatwave | [How to cope in a heatwave - Council for Intellectual Disability (cid.org.au)](https://cid.org.au/resource/how-to-cope-in-a-heatwave/) |
|  |  | Ageing and health | [6-Ageing-and-health.pdf (cid.org.au)](https://cid.org.au/wp-content/uploads/2019/10/6-Ageing-and-health.pdf) |
|  |  | Healthy Lifestyles | [8-Healthy-lifestyles.pdf (cid.org.au)](https://cid.org.au/wp-content/uploads/2019/10/8-Healthy-lifestyles.pdf) |
|  |  | Preventative Health | [10-Preventive-health.pdf (cid.org.au)](https://cid.org.au/wp-content/uploads/2019/10/10-Preventive-health.pdf) |
|  |  | Alcohol and other drugs | [14-Alcohol-and-other-drugs-.pdf (cid.org.au)](https://cid.org.au/wp-content/uploads/2019/10/14-Alcohol-and-other-drugs-.pdf) |
|  |  | Challenging behaviour and health | [12-Challenging-behaviour-and-health.pdf (cid.org.au)](https://cid.org.au/wp-content/uploads/2019/10/12-Challenging-behaviour-and-health.pdf) |
|  |  | Me and my medication | [Me-and-my-medication-health-guide.pdf (cid.org.au)](https://cid.org.au/wp-content/uploads/2019/11/Me-and-my-medication-health-guide.pdf) |
|  |  | Caring for your teeth fact sheet | [Caring for your teeth Easy Read Fact Sheet (cid.org.au)](https://cid.org.au/wp-content/uploads/2021/09/Caring-for-your-teeth-Easy-Read-Fact-Sheet.pdf) |
|  |  | Dental Care | [11-Dental-care.pdf (cid.org.au)](https://cid.org.au/wp-content/uploads/2019/10/11-Dental-care.pdf) |
|  |  | Going to the Dentist | [20-Going-to-the-dentist.pdf (cid.org.au)](https://cid.org.au/wp-content/uploads/2019/10/20-Going-to-the-dentist.pdf) |
|  |  | Health care plans for  chronic health problems | [Chronic Health Plans Easy Read Fact Sheet (cid.org.au)](https://cid.org.au/wp-content/uploads/2021/09/Chronic-Health-Plans-Easy-Read-Fact-Sheet.pdf) |
|  |  | Mental Health Guide | [Mental-Health-Guide-V4-1.pdf (cid.org.au)](https://cid.org.au/wp-content/uploads/2023/07/Mental-Health-Guide-V4-1.pdf) |
|  |  | Wellbeing is Important | [ER-Wellbeing.pdf (cid.org.au)](https://cid.org.au/wp-content/uploads/2023/03/ER-Wellbeing.pdf) |
|  |  | Tips to help mental health | [Tips-to-help-mental-health.pdf (cid.org.au)](https://cid.org.au/wp-content/uploads/2019/07/Tips-to-help-mental-health.pdf) |
|  |  | Mental Health | [13-Mental-health.pdf (cid.org.au)](https://cid.org.au/wp-content/uploads/2019/10/13-Mental-health.pdf) |
|  |  | Anchor exercise to help you feel calm | [Anchor exercise to help you feel calm - Council for Intellectual Disability (cid.org.au)](https://cid.org.au/resource/anchor-calming-exercise/) |
|  |  | After visiting the doctor | [After-visiting-the-doctor-health-guide.pdf (cid.org.au)](https://cid.org.au/wp-content/uploads/2019/11/After-visiting-the-doctor-health-guide.pdf) |
|  |  | Finding a new doctor | [Finding-a-new-doctor-health-guide.pdf (cid.org.au)](https://cid.org.au/wp-content/uploads/2019/11/Finding-a-new-doctor-health-guide.pdf) |
|  |  | Your right to good health care fact sheet | [Your right to good health care Easy Read Fact Sheet (cid.org.au)](https://cid.org.au/wp-content/uploads/2021/09/Your-right-to-good-health-care-Easy-Read-Fact-Sheet.pdf) |
|  |  | Health Services fact sheet for adults | [Adult Health Services Easy Read Factsheet (cid.org.au)](https://cid.org.au/wp-content/uploads/2021/09/Adult-Health-Services-Easy-Read-Fact-Sheet.pdf) |
|  |  | Children- what kind of health services are there? | [3-Children-what-kinds-of-health-services-are-there.pdf (cid.org.au)](https://cid.org.au/wp-content/uploads/2019/10/3-Children-what-kinds-of-health-services-are-there.pdf) |
|  |  | Adults- what kind of health services are there? | [5-Adults-what-kinds-of-health-services-are-there.pdf (cid.org.au)](https://cid.org.au/wp-content/uploads/2019/10/5-Adults-what-kinds-of-health-services-are-there.pdf) |
|  |  | Finding the right doctor | [19-Finding-the-right-doctor.pdf (cid.org.au)](https://cid.org.au/wp-content/uploads/2019/10/19-Finding-the-right-doctor.pdf) |
|  |  | Specialised intellectual disability health services | [21-Specialised-intellectual-disability-health-services.pdf (cid.org.au)](https://cid.org.au/wp-content/uploads/2019/10/21-Specialised-intellectual-disability-health-services.pdf) |
|  |  | Getting the most out of Medicare | [22-Getting-the-most-out-of-Medicare.pdf (cid.org.au)](https://cid.org.au/wp-content/uploads/2019/10/22-Getting-the-most-out-of-Medicare.pdf) |
|  |  | Going to the doctor- tips and tricks | [23-Going-to-the-doctor-tips-and-tricks.pdf (cid.org.au)](https://cid.org.au/wp-content/uploads/2019/10/23-Going-to-the-doctor-tips-and-tricks.pdf) |
|  |  | Helping the doctor understand the person | [24-Helping-the-doctor-understand-the-person.pdf (cid.org.au)](https://cid.org.au/wp-content/uploads/2019/10/24-Helping-the-doctor-understand-the-person.pdf) |
|  |  | Going to the hospital | [26-Going-to-hospital-.pdf (cid.org.au)](https://cid.org.au/wp-content/uploads/2019/10/26-Going-to-hospital-.pdf) |
|  |  | Consent to medical treatment | [28-Consent-to-medical-treatment.pdf (cid.org.au)](https://cid.org.au/wp-content/uploads/2019/10/28-Consent-to-medical-treatment.pdf) |
|  |  | Rights and complaints | [29-Rights-and-complaints.pdf (cid.org.au)](https://cid.org.au/wp-content/uploads/2019/10/29-Rights-and-complaints.pdf) |
|  |  | My Health Matters | [My Health Matters folder - Council for Intellectual Disability (cid.org.au)](https://cid.org.au/resource/my-health-matters-folder/) |
|  |  | My Health Appointment Form | [My Health Appointment Form - Easy Read \| CID](https://cid.org.au/resource/my-health-appointment-form/) |
|  |  | Managing Menstruation | [18-Managing-menstruation.pdf (cid.org.au)](https://cid.org.au/wp-content/uploads/2019/10/18-Managing-menstruation.pdf) |
|  |  | Me and my doctor | [Me-and-my-doctor-guide-V4-2.pdf (cid.org.au)](https://cid.org.au/wp-content/uploads/2023/07/Me-and-my-doctor-guide-V4-2.pdf) |
|  |  | Yearly Health Checks: Health Fact Sheet | [Yearly Health Check Easy Read Fact Sheet (cid.org.au)](https://cid.org.au/wp-content/uploads/2021/09/Yearly-Health-Check-Easy-Read-Fact-Sheet.pdf) |
|  |  | Types of Health Checks fact sheet | [Types of Health Checks Easy Read Fact Sheet (cid.org.au)](https://cid.org.au/wp-content/uploads/2021/09/Types-of-Health-Checks-Easy-Read-Fact-Sheet.pdf) |
|  |  | Going to hospital for surgery | [Going-to-hospital-for-surgery-health-guide.pdf (cid.org.au)](https://cid.org.au/wp-content/uploads/2019/11/Going-to-hospital-for-surgery-health-guide.pdf) |
|  |  | Annual Health Assessments | [9-Annual-health-assessments.pdf (cid.org.au)](https://cid.org.au/wp-content/uploads/2019/10/9-Annual-health-assessments.pdf) |
|  |  | Look after your mental health during COVID-19 | [Look after your mental health during COVID-19 - Council for Intellectual Disability (cid.org.au)](https://cid.org.au/our-stories/look-after-your-mental-health-during-coronavirus/) |
|  |  | Covid Information | [COVID information - Council for Intellectual Disability (cid.org.au)](https://cid.org.au/covid-19/) |
|  |  | Visruses and staying healthy | [Viruses-staying-healthy-Council-Intellectual-Disability.pdf (cid.org.au)](https://cid.org.au/wp-content/uploads/2020/03/Viruses-staying-healthy-Council-Intellectual-Disability.pdf) |
|  |  | Some signs of sickness | [Some-signs-of-sickness-health-guide.pdf (cid.org.au)](https://cid.org.au/wp-content/uploads/2019/11/Some-signs-of-sickness-health-guide.pdf) |
|  |  | Children- Signs of illness | [2–Children-signs-of-Illness.pdf (cid.org.au)](https://cid.org.au/wp-content/uploads/2019/10/2%E2%80%93Children-signs-of-Illness.pdf) |
|  |  | Adults- Signs of illness | [4-Adults-signs-of-Illness-.pdf (cid.org.au)](https://cid.org.au/wp-content/uploads/2019/10/4-Adults-signs-of-Illness-.pdf) |
|  |  | Sexuality | [16-Sexuality.pdf (cid.org.au)](https://cid.org.au/wp-content/uploads/2019/10/16-Sexuality.pdf) |
|  |  | Contraception | <https://cid.org.au/wp-content/uploads/2019/10/17-Contraception.pdf> |
|  |  | My Health Cards | [Health Conversation Cards - My Health Cards \| CID](https://cid.org.au/resource/my-health-cards/) |
|  |  | How to use the My Health Cards | https://cid.org.au/wp-content/uploads/2024/05/Health-My-Health-Cards-Guide-WEB.pdf |
|  |  | Tailorable Easy Read health letters | https://cid.org.au/resource/easy-read-health-letters/ |
|  |  | Signs of sickness | https://cid.org.au/wp-content/uploads/2024/05/Health-ER-Signs-of-Sickness.pdf |
|  |  | Me and my medication guide | https://cid.org.au/wp-content/uploads/2019/11/health-ER-Me-and-my-medication-guide.pdf |
|  |  | How to find a new doctor guide | https://cid.org.au/wp-content/uploads/2024/04/health-ER-How-to-find-a-new-doctor-guide.pdf |
|  |  | My Health Worker Guide | https://cid.org.au/wp-content/uploads/2024/03/health-ER-My-Health-Worker-Guide-V4.pdf |
|  |  | personal health records | [25-Personal-health-records.pdf (cid.org.au)](https://cid.org.au/wp-content/uploads/2019/10/25-Personal-health-records.pdf) |
|  |  | Causes of intellectual disability and health care | [15-Causes-of-intellectual-disability-and-health-care.pdf (cid.org.au)](https://cid.org.au/wp-content/uploads/2019/10/15-Causes-of-intellectual-disability-and-health-care.pdf) |
| Department of Developmental Disability Neuropsychiatry (3DN) | <https://www.3dn.unsw.edu.au/> | Wellbeing record | <https://www.3dn.unsw.edu.au/sites/default/files/documents/Wellbeing-record.pdf> |
|  |  | Getting help with your mental health | [Getting help with your mental health \| Department of Developmental Disability Neuropsychiatry (3DN) (unsw.edu.au)](https://www.3dn.unsw.edu.au/consumers_gettinghelp) |
|  |  | People who can help me with my mental health | [3DN_WhoCanHelp_MentalHealth_ERv1.pdf (unsw.edu.au)](https://www.3dn.unsw.edu.au/sites/default/files/documents/3DN_WhoCanHelp_MentalHealth_ERv1.pdf) |
|  |  | Questions you can ask when you want to find a mental health service in the community | [3DN_Questions_Community_ERv1.pdf (unsw.edu.au)](https://www.3dn.unsw.edu.au/sites/default/files/documents/3DN_Questions_Community_ERv1.pdf) |
|  |  | Questions you can ask when you go to hospital for your mental health | [3DN_Questions_Hospital_ERv1.pdf (unsw.edu.au)](https://www.3dn.unsw.edu.au/sites/default/files/documents/3DN_Questions_Hospital_ERv1.pdf) |
|  |  | Word list | [3DN_Wordlist_ERv1.pdf (unsw.edu.au)](https://www.3dn.unsw.edu.au/sites/default/files/documents/3DN_Wordlist_ERv1.pdf) |
|  |  | Making a complaint about mental health care | [3DN_Complaints_MentalHealth_ERv1.pdf (unsw.edu.au)](https://www.3dn.unsw.edu.au/sites/default/files/documents/3DN_Complaints_MentalHealth_ERv1.pdf) |
| Department of Health and Aged Care | <https://www.health.gov.au/> | Adult Comprehensive Health Assessment Program (CHAP) – Annual Health Assessment for People with Intellectual Disability | [CHAP Comprehensive Health Assessment Program – Annual Health Assessment for People with Intellectual Disability – ADULT](https://www.health.gov.au/sites/default/files/2023-05/adult-comprehensive-health-assessment-program-chap-annual-health-assessment-for-people-with-intellectual-disability.pdf) |
| Developmental Disability WA | <https://www.ddwa.org.au/> | Planning to go to the Dentist | <http://www.ddwa.org.au/resources/planning-to-go-to-the-dentist/> |
|  |  | Your Dental Health | <http://www.ddwa.org.au/resources/your-dental-health-wa/> |
|  |  | Nourish with Gastrostomy | <https://ddwa.org.au/online-learning/nourish-with-gastrostomy-outline/> |
|  |  | Working Together: Therapy in the Early Years | <http://www.ddwa.org.au/resources/working-together-therapy-in-the-early-years/> |
|  |  | COVID-19 Easy Read Resources | [COVID-19 Easy Read Resources • Developmental Disability WA - DDWA](https://ddwa.org.au/covid-19-information/covid-19-easy-read-resources/) |
|  |  | Supporting people with disability during COVID 19 | [Supporting people with a disability during covid-19 • Developmental Disability WA - DDWA](https://ddwa.org.au/covid-19-information/supporting-people-with-a-disability-during-covid-19/) |
|  |  | There’s no such thing as a silly question | [There’s no such thing as a silly question • Developmental Disability WA - DDWA](https://ddwa.org.au/resources/theres-no-such-thing-as-a-silly-question/) |
|  |  | Health Care Guide: For People Who Need Extra Support | <http://www.ddwa.org.au/resources/health-care-guide> |
|  |  | Intellectual Disability Mental Health First Aid Manual | <https://www.deai.com.au/wp-content/uploads/2014/11/Intellectual-Disability-Mental-Health-First-Aid-Manual-2nd-Ed-2010.pdf> |
| Disability Gateway | <https://www.disabilitygateway.gov.au/> | Health and wellbeing | [Health and wellbeing \| Disability Gateway](https://www.disabilitygateway.gov.au/health-wellbeing) |
| Down Syndrome Australia | <https://www.downsyndrome.org.au/> | Obstructive Sleep Apnoea and children with Down Syndrom | [Untitled (downsyndrome.org.au)](https://www.downsyndrome.org.au/wp-content/uploads/2021/02/OSA-and-Down-Syndrome_2021.pdf) |
|  |  | Sleep in children with Down Syndrome (0-2 years) | <https://www.downsyndrome.org.au/blog/resource/sleep-in-children-with-down-syndrome-0-2-yrs/> |
|  |  | Healthy Sleeping Habits for Teens with Down Syndrom | [Untitled (downsyndrome.org.au)](https://www.downsyndrome.org.au/wp-content/uploads/2021/02/Sleep-in-Teenagers-with-Down-Syndrome_2021.pdf) |
|  |  | Dental health for people with Down syndrome | [DSA-dental-health-C03.pdf (downsyndrome.org.au)](https://www.downsyndrome.org.au/wp-content/uploads/2020/02/DSA-dental-health-C03.pdf) |
|  |  | Down Syndrome Good Health App | [Down Syndrome Good Health App – Down Syndrome Australia](https://www.downsyndrome.org.au/resources/apps/down-syndrome-good-health-app/) |
|  |  | Down syndrome and mental health | [Down syndrome and mental health](https://www.downsyndrome.org.au/wp-content/uploads/2020/02/DSAMentalhealthresourceweb.pdf) |
|  |  | COVID-19 | [COVID-19 – Down Syndrome Australia](https://www.downsyndrome.org.au/resources/covid-19/) |
|  |  | Dementia and Down Syndrome | [Dementia and Down syndrome – Down Syndrome Australia](https://www.downsyndrome.org.au/resources/health/dementia-and-down-syndrome/) |
|  |  | Healthy Relationships and Sexuality | [Healthy Relationships and Sexuality: Information for people with Down syndrome and their families](https://www.downsyndrome.org.au/wp-content/uploads/2020/07/DSA_Healthy-relationships-guide.pdf) |
|  |  | Adolescence | [Adolescence (downsyndrome.org.au)](https://www.downsyndrome.org.au/wp-content/uploads/2020/05/DSA_Adolescence_June-22_WEB.pdf) |
|  |  | Staying Healthy | [Microsoft Word - Staying Healthy Tips Easy Read WORD_FINAL.docx (downsyndrome.org.au)](https://www.downsyndrome.org.au/wp-content/uploads/2020/11/Staying-Healthy-Tips-Easy-Read-PDF_Web.pdf) |
|  |  | Ageing | [Ageing (downsyndrome.org.au)](https://www.downsyndrome.org.au/wp-content/uploads/2020/04/DSA_Ageing_Jun22_WEB-1.pdf) |
|  |  | Down syndrome: A practical guide to ageing well | [Down syndrome – A practical guide to ageing well](https://www.downsyndrome.org.au/wp-content/uploads/2020/04/DSAApracticalguidetoageingwellweb.pdf) |
|  |  | Medical Care Guidelines for Adults with Down syndrome | [2022 Family-Friendly GLOBAL Adult Guideline.pdf (downsyndrome.org.au)](https://www.downsyndrome.org.au/wp-content/uploads/2022/07/2022-Family-Friendly-GLOBAL-Adult-Guideline.pdf) |
| EasyHealth | <https://www.easyhealth.org.uk/> | Alcohol | <https://www.easyhealth.org.uk/resources/13-alcohol> |
|  |  | Alcohol and Epilepsy | <https://hubble-live-assets.s3.eu-west-1.amazonaws.com/easy-health/file_asset/file/22/alcohol-and-epilepsy.pdf> |
|  |  | Coming for a drink | [coming_for_a_drink.pdf (hubble-live-assets.s3.eu-west-1.amazonaws.com)](https://hubble-live-assets.s3.eu-west-1.amazonaws.com/easy-health/file_asset/file/16/coming_for_a_drink.pdf) |
|  |  | Smoking and alcohol | [Microsoft Word - smoking and alcohol.doc (hubble-live-assets.s3.eu-west-1.amazonaws.com)](https://hubble-live-assets.s3.eu-west-1.amazonaws.com/easy-health/file_asset/file/763/Smoking-and-Alcohol.pdf) |
|  |  | Easy Read - Allergies' | [Easy Read - Allergies' \| Easy Health](https://www.easyhealth.org.uk/resources/allergies) |
|  |  | All about asthma | All about asthma |
|  |  | My asthma inhalers card (when to use each inhaler) | <https://hubble-live-assets.s3.eu-west-1.amazonaws.com/easy-health/file_asset/file/988/my-asthma-inhaler_easy-to-read_digital_-_2022_2.pdf> |
|  |  | What to do in an asthma attack | [asthma-attack_easy-to-read_digital2022_2.pdf (hubble-live-assets.s3.eu-west-1.amazonaws.com)](https://hubble-live-assets.s3.eu-west-1.amazonaws.com/easy-health/file_asset/file/990/asthma-attack_easy-to-read_digital2022_2.pdf) |
|  |  | Easy Read - Fibroids | <https://hubble-live-assets.s3.eu-west-1.amazonaws.com/easy-health/file_asset/file/1325/fibroids.pdf> |
|  |  | Abdominal aortic  aneurysm (AAA) screening | <https://hubble-live-assets.s3.eu-west-1.amazonaws.com/easy-health/file_asset/file/905/Abdominal_Aortic_Aneurysms_screening_easy_guide_August_2020.pdf> |
|  |  | Look after your back | [Look After Your Back \| Easy Health](https://www.easyhealth.org.uk/resources/24-look-after-your-back) |
|  |  | Low back pain | [Low Back Pain \| Easy Health](https://www.easyhealth.org.uk/resources/26-low-back-pain) |
|  |  | Lower back pain | [Lower Back Pain Easy Read Leaflet (hubble-live-assets.s3.eu-west-1.amazonaws.com)](https://hubble-live-assets.s3.eu-west-1.amazonaws.com/easy-health/file_asset/file/31/lower-back-pain.pdf) |
|  |  | My urinary catheter passport | [My urinary catheter passpor](https://www.easyhealth.org.uk/resources/category/30-bladder) |
|  |  | Giving a Wee Sample - easy read [G.O.S.H] | [Giving_a_Wee_Sample_-_easy_read__G.O.S.H_.pdf (hubble-live-assets.s3.eu-west-1.amazonaws.com)](https://hubble-live-assets.s3.eu-west-1.amazonaws.com/easy-health/file_asset/file/1396/Giving_a_Wee_Sample_-_easy_read__G.O.S.H_.pdf) |
|  |  | Keeping your bladder healthy | [keeping your bladder healthy er (hubble-live-assets.s3.eu-west-1.amazonaws.com)](https://hubble-live-assets.s3.eu-west-1.amazonaws.com/easy-health/file_asset/file/1402/Keeping_your_bladder_healthy_-_easy_read__G.O.S.H_.pdf) |
|  |  | Tummy trouble? Blood in your wee? Speak to your doctor - poster from the NHS | [Tummy trouble? Blood in your wee? Speak to your doctor (hubble-live-assets.s3.eu-west-1.amazonaws.com)](https://hubble-live-assets.s3.eu-west-1.amazonaws.com/easy-health/file_asset/file/1003/NHS-abdo-uro_Poster-EasyRead-Accessible.pdf) |
|  |  | Have You Seen Blood In Your Pee? | [ISL107 ER Be Clear on cancer Blood in Pee (hubble-live-assets.s3.eu-west-1.amazonaws.com)](https://hubble-live-assets.s3.eu-west-1.amazonaws.com/easy-health/file_asset/file/78/Have-you-seen-blood-your-pee-even-once-then-its-time-to-tell-your-doctor.pdf) |
|  |  | Bowel and Bladder Awareness | [Bowel and Bladder Awareness \| Easy Health](https://www.easyhealth.org.uk/resources/36-bowel-and-bladder-awareness) |
|  |  | Flexible Cystoscopy | <https://hubble-live-assets.s3.eu-west-1.amazonaws.com/easy-health/file_asset/file/55/Flexible-Cystoscopy.pdf> |
|  |  | How do I go to  the toilet by  myself? | [How-do-i-go-to-the-toilet-by-myself-a-guide-for-boys.pdf (hubble-live-assets.s3.eu-west-1.amazonaws.com)](https://hubble-live-assets.s3.eu-west-1.amazonaws.com/easy-health/file_asset/file/56/How-do-i-go-to-the-toilet-by-myself-a-guide-for-boys.pdf) |
|  |  | How Do I Go To The Toilet By Myself? A Guide For Girls | [How Do I Go To The Toilet By Myself? A Guide For Girls \| Easy Health](https://www.easyhealth.org.uk/resources/41-how-do-i-go-to-the-toilet-by-myself-a-guide-for-girls) |
|  |  | Trouble With Your Waterworks | [trouble_with_your_waterworks.pdf (hubble-live-assets.s3.eu-west-1.amazonaws.com)](https://hubble-live-assets.s3.eu-west-1.amazonaws.com/easy-health/file_asset/file/58/trouble_with_your_waterworks.pdf) |
|  |  | Urology Investigation | [urology-investigation.pdf (hubble-live-assets.s3.eu-west-1.amazonaws.com)](https://hubble-live-assets.s3.eu-west-1.amazonaws.com/easy-health/file_asset/file/59/urology-investigation.pdf) |
|  |  | Making a Decision about Varicose Veins | <https://hubble-live-assets.s3.eu-west-1.amazonaws.com/easy-health/file_asset/file/1391/Making_a_Decision_about_Varicose-Veins-easy-read__Winton_Centre_.pdf> |
|  |  | How to take your own blood pressure | [How to take your blood pressure (hubble-live-assets.s3.eu-west-1.amazonaws.com)](https://hubble-live-assets.s3.eu-west-1.amazonaws.com/easy-health/file_asset/file/1337/Easy_Read_how_to_take_your_Blood_Pressure.pdf) |
|  |  | Having your blood pressure taken: Information for patients | <https://hubble-live-assets.s3.eu-west-1.amazonaws.com/easy-health/file_asset/file/52/Having-your-blood-pressure-taken.pdf> |
|  |  | What is Blood Pressure? | <https://hubble-live-assets.s3.eu-west-1.amazonaws.com/easy-health/file_asset/file/19/what_is_blood_pressure.pdf> |
|  |  | Atenolol | <https://hubble-live-assets.s3.eu-west-1.amazonaws.com/easy-health/file_asset/file/223/Atenolol.pdf> |
|  |  | Propranolol | [Risperidone (hubble-live-assets.s3.eu-west-1.amazonaws.com)](https://hubble-live-assets.s3.eu-west-1.amazonaws.com/easy-health/file_asset/file/248/Propranolol.pdf) |
|  |  | Making a Decision about an Inguinal-Hernia easy-read | <https://hubble-live-assets.s3.eu-west-1.amazonaws.com/easy-health/file_asset/file/1406/Making-a-decision-about-inguinal-Hernia-easy-read__Winton_Centre_.pdf> |
|  |  | Making a Decision about Gallstones [Winton Centre] | [hubble-live-assets.s3.eu-west-1.amazonaws.com/easy-health/file_asset/file/1408/Making_a_Decision_about_Gallstones-easy-read__Winton_Centre_.pdf](https://hubble-live-assets.s3.eu-west-1.amazonaws.com/easy-health/file_asset/file/1408/Making_a_Decision_about_Gallstones-easy-read__Winton_Centre_.pdf) |
|  |  | Giving a Poo Sample - easy read [G.O.S.H] | [16ER0074 giving a poo sample (hubble-live-assets.s3.eu-west-1.amazonaws.com)](https://hubble-live-assets.s3.eu-west-1.amazonaws.com/easy-health/file_asset/file/1398/Giving_a_Poo_Sample_-_easy_read__G.O.S.H_.pdf) |
|  |  | Healthy Bowels - easy read [G.O.S.H] | [Healthy Bowels - easy read [G.O.S.H] \| Easy Health](https://www.easyhealth.org.uk/resources/healthy_bowels-easy-read-g-o-s-h) |
|  |  | NHS: Bowel Cancer Screening - Easy Read | <https://hubble-live-assets.s3.eu-west-1.amazonaws.com/easy-health/file_asset/file/1351/2023.04.17_nhs-bowel-screening_FIT_EasyRead-Leaflet-WebAcc.pdf> |
|  |  | Easy Read - Endometriosis | [endometriosis.pdf (hubble-live-assets.s3.eu-west-1.amazonaws.com)](https://hubble-live-assets.s3.eu-west-1.amazonaws.com/easy-health/file_asset/file/1323/endometriosis.pdf) |
|  |  | Easy Read - Fibroids | <https://hubble-live-assets.s3.eu-west-1.amazonaws.com/easy-health/file_asset/file/1325/fibroids.pdf> |
|  |  | What are the signs of Constipation? | [A4_Patient_Leaflet_20.4_AW_1_a.indd (hubble-live-assets.s3.eu-west-1.amazonaws.com)](https://hubble-live-assets.s3.eu-west-1.amazonaws.com/easy-health/file_asset/file/1265/NHS_Constpation.pdf) |
|  |  | If you are worried about cancer speak to your doctor | <https://www.easyhealth.org.uk/resources/speak-to-your-doctor-if-you-are-worried-about-cancer> |
|  |  | Bowel cancer screening: an easy guide to having a CTC scan | <https://hubble-live-assets.s3.eu-west-1.amazonaws.com/easy-health/file_asset/file/911/CTC_scan_easy_guide.pdf> |
|  |  | Bowel cancer screening: an easy guide to having a colonoscopy | [Bowel_cancer_screening_colonoscopy_easy_guide (hubble-live-assets.s3.eu-west-1.amazonaws.com)](https://hubble-live-assets.s3.eu-west-1.amazonaws.com/easy-health/file_asset/file/896/Bowel_cancer_screening_colonoscopy_easy_guide__3_.pdf) |
|  |  | An Easy Guide to Bowel Cancer Screening | [Bowel_cancer_screening_colonoscopy_easy_guide (hubble-live-assets.s3.eu-west-1.amazonaws.com)](https://hubble-live-assets.s3.eu-west-1.amazonaws.com/easy-health/file_asset/file/896/Bowel_cancer_screening_colonoscopy_easy_guide__3_.pdf) |
|  |  | An Easy Guide to  Having a Colonoscopy | [Colonoscopy 16pp lft (hubble-live-assets.s3.eu-west-1.amazonaws.com)](https://hubble-live-assets.s3.eu-west-1.amazonaws.com/easy-health/file_asset/file/70/An-Easy-Guide-to-Having-a-Colonoscopy.pdf) |
|  |  | Bowel Cancer | <https://hubble-live-assets.s3.eu-west-1.amazonaws.com/easy-health/file_asset/file/73/bowel-cancer.pdf> |
|  |  | Constipation | <https://hubble-live-assets.s3.eu-west-1.amazonaws.com/easy-health/file_asset/file/54/constipation.pdf> |
|  |  | Preventing Constipation | <https://hubble-live-assets.s3.eu-west-1.amazonaws.com/easy-health/file_asset/file/60/preventing_constipation.pdf> |
|  |  | Trouble Pooing (Constipation) | <https://hubble-live-assets.s3.eu-west-1.amazonaws.com/easy-health/file_asset/file/10/touble-pooing-constipation.pdf> |
|  |  | Bowel Cancer Screening | <https://hubble-live-assets.s3.eu-west-1.amazonaws.com/easy-health/file_asset/file/46/bowel-screening.pdf> |
|  |  | Making a Decision about Managing Type 1 Diabetes | <https://hubble-live-assets.s3.eu-west-1.amazonaws.com/easy-health/file_asset/file/1386/Making_a_decision_about_managing_Type_1_Diabetes-easy-read__Witon_Centre_.pdf> |
|  |  | NHS: New ways your pharmacist can help you - Easy Read Leaflet | [New ways your pharmacist can help you (hubble-live-assets.s3.eu-west-1.amazonaws.com)](https://hubble-live-assets.s3.eu-west-1.amazonaws.com/easy-health/file_asset/file/1348/240415_NHS_Pharmacy_EasyRead_2-page-leaflet_Web.pdf) |
|  |  | Information about Procyclidine | [NURSES’ POWER TO DETAIN A PERSDON BEING TREATED FOR MENTAL DISORDER AS A HOSPITAL IN-PATIENT (hubble-live-assets.s3.eu-west-1.amazonaws.com)](https://hubble-live-assets.s3.eu-west-1.amazonaws.com/easy-health/file_asset/file/1344/Procyclidine-easy-read.pdf) |
|  |  | Video - "Don't Miss Out" - Stories about Annual Health Checks [By MENCAP] | [NHS_free-health-check.jpg (2480×3508) (hubble-live-assets.s3.eu-west-1.amazonaws.com)](https://hubble-live-assets.s3.eu-west-1.amazonaws.com/easy-health/file_asset/file/1279/NHS_free-health-check.jpg) |
|  |  | Easy read - Surgery for primary breast cancer [By Breast Cancer Now] | [20872_02 BCN256 Surgery for primary breast cancer LOW RES.indd (hubble-live-assets.s3.eu-west-1.amazonaws.com)](https://hubble-live-assets.s3.eu-west-1.amazonaws.com/easy-health/file_asset/file/1259/easy_read_bcn256_surgery_for_primary_breast_cancer.pdf) |
|  |  | Lemsip | [Lemsip.pdf (hubble-live-assets.s3.eu-west-1.amazonaws.com)](https://hubble-live-assets.s3.eu-west-1.amazonaws.com/easy-health/file_asset/file/288/Lemsip.pdf) |
|  |  | I am taking Lithium | [Risperidone (hubble-live-assets.s3.eu-west-1.amazonaws.com)](https://hubble-live-assets.s3.eu-west-1.amazonaws.com/easy-health/file_asset/file/245/Lithium.pdf) |
|  |  | I am taking S.S.R.I. Antidepressants | [Microsoft Word - SSRI easy read.doc (hubble-live-assets.s3.eu-west-1.amazonaws.com)](https://hubble-live-assets.s3.eu-west-1.amazonaws.com/easy-health/file_asset/file/32/Im-taking-ssri-antidepressants.pdf) |
|  |  | I am taking Tricyclic Antidepressants | [Microsoft Word - tricyclid antidepressants easy read.doc (hubble-live-assets.s3.eu-west-1.amazonaws.com)](https://hubble-live-assets.s3.eu-west-1.amazonaws.com/easy-health/file_asset/file/249/im-taking-tricyclic-antidepressants.pdf) |
|  |  | Getting To Know Your Pharmacist | [YourPharmacist_5b.cdr (hubble-live-assets.s3.eu-west-1.amazonaws.com)](https://hubble-live-assets.s3.eu-west-1.amazonaws.com/easy-health/file_asset/file/214/getting-to-know-your-pharmacist.pdf) |
|  |  | How To Make Choices About Taking Medication | [HowToMakeChoices_low-res_1.vp (hubble-live-assets.s3.eu-west-1.amazonaws.com)](https://hubble-live-assets.s3.eu-west-1.amazonaws.com/easy-health/file_asset/file/215/How-to-make-choices-about-taking-medication.pdf) |
|  |  | Information About My Medication | [Medication Template (hubble-live-assets.s3.eu-west-1.amazonaws.com)](https://hubble-live-assets.s3.eu-west-1.amazonaws.com/easy-health/file_asset/file/216/information-about-my-medication.pdf) |
|  |  | Medication Matters | [Medication-matters.pdf (hubble-live-assets.s3.eu-west-1.amazonaws.com)](https://hubble-live-assets.s3.eu-west-1.amazonaws.com/easy-health/file_asset/file/217/Medication-matters.pdf) |
|  |  | My Medication | [MyMedication.vp (hubble-live-assets.s3.eu-west-1.amazonaws.com)](https://hubble-live-assets.s3.eu-west-1.amazonaws.com/easy-health/file_asset/file/218/My-medication.pdf) |
|  |  | When and How To Take Your Medicine | [When and how to take your medicine (hubble-live-assets.s3.eu-west-1.amazonaws.com)](https://hubble-live-assets.s3.eu-west-1.amazonaws.com/easy-health/file_asset/file/219/When-and-how-to-take-your-medicine.pdf) |
|  |  | Your guide to taking medicine for behaviour problems | [untitled (hubble-live-assets.s3.eu-west-1.amazonaws.com)](https://hubble-live-assets.s3.eu-west-1.amazonaws.com/easy-health/file_asset/file/220/Your-guide-to-taking-medicine-for-behaviour-problems.pdf) |
|  |  | All My Medications | [AllMyMedications_06.vp (hubble-live-assets.s3.eu-west-1.amazonaws.com)](https://hubble-live-assets.s3.eu-west-1.amazonaws.com/easy-health/file_asset/file/213/All-my-medications.pdf) |
|  |  | Ibuprofen | [Ibuprofen.pdf (hubble-live-assets.s3.eu-west-1.amazonaws.com)](https://hubble-live-assets.s3.eu-west-1.amazonaws.com/easy-health/file_asset/file/283/Ibuprofen.pdf) |
|  |  | I’m Taking Atypical Antipsychotics | [I’m Taking Atypical Antipsychotics \| Easy Health](https://www.easyhealth.org.uk/resources/288-i-m-taking-atypical-antipsychotics) |
|  |  | Fluphenazine | [Risperidone (hubble-live-assets.s3.eu-west-1.amazonaws.com)](https://hubble-live-assets.s3.eu-west-1.amazonaws.com/easy-health/file_asset/file/233/Fluphenazine.pdf) |
|  |  | Olanzapine | [Risperidone (hubble-live-assets.s3.eu-west-1.amazonaws.com)](https://hubble-live-assets.s3.eu-west-1.amazonaws.com/easy-health/file_asset/file/246/Olanzapine.pdf) |
|  |  | Risperidone | [Risperidone (hubble-live-assets.s3.eu-west-1.amazonaws.com)](https://hubble-live-assets.s3.eu-west-1.amazonaws.com/easy-health/file_asset/file/251/Risperidone.pdf) |
|  |  | Amisulpride | [Risperidone (hubble-live-assets.s3.eu-west-1.amazonaws.com)](https://hubble-live-assets.s3.eu-west-1.amazonaws.com/easy-health/file_asset/file/221/Amisulpride.pdf) |
|  |  | Aripiprazole | [Risperidone (hubble-live-assets.s3.eu-west-1.amazonaws.com)](https://hubble-live-assets.s3.eu-west-1.amazonaws.com/easy-health/file_asset/file/222/Aripiprazole.pdf) |
|  |  | Carbamazepine | [Risperidone (hubble-live-assets.s3.eu-west-1.amazonaws.com)](https://hubble-live-assets.s3.eu-west-1.amazonaws.com/easy-health/file_asset/file/224/Carbamazepine.pdf) |
|  |  | Chlorpromazine | Risperidone (hubble-live-assets.s3.eu-west-1.amazonaws.com) |
|  |  | Citalopram | [Risperidone (hubble-live-assets.s3.eu-west-1.amazonaws.com)](https://hubble-live-assets.s3.eu-west-1.amazonaws.com/easy-health/file_asset/file/226/Citalopram.pdf) |
|  |  | Clozapine | [Risperidone (hubble-live-assets.s3.eu-west-1.amazonaws.com)](https://hubble-live-assets.s3.eu-west-1.amazonaws.com/easy-health/file_asset/file/227/Clozapine.pdf) |
|  |  | Diazepam | [Risperidone (hubble-live-assets.s3.eu-west-1.amazonaws.com)](https://hubble-live-assets.s3.eu-west-1.amazonaws.com/easy-health/file_asset/file/228/Diazepam.pdf) |
|  |  | Dosulepin | [Risperidone (hubble-live-assets.s3.eu-west-1.amazonaws.com)](https://hubble-live-assets.s3.eu-west-1.amazonaws.com/easy-health/file_asset/file/229/Dosulepin.pdf) |
|  |  | Escitalopram | [Risperidone (hubble-live-assets.s3.eu-west-1.amazonaws.com)](https://hubble-live-assets.s3.eu-west-1.amazonaws.com/easy-health/file_asset/file/230/Escitalopram.pdf) |
|  |  | Fluoxetine | [Risperidone (hubble-live-assets.s3.eu-west-1.amazonaws.com)](https://hubble-live-assets.s3.eu-west-1.amazonaws.com/easy-health/file_asset/file/231/Fluoxetine.pdf) |
|  |  | Flupentixol | [Risperidone (hubble-live-assets.s3.eu-west-1.amazonaws.com)](https://hubble-live-assets.s3.eu-west-1.amazonaws.com/easy-health/file_asset/file/232/Flupentixol.pdf) |
|  |  | Fluvoxamine | [Risperidone (hubble-live-assets.s3.eu-west-1.amazonaws.com)](https://hubble-live-assets.s3.eu-west-1.amazonaws.com/easy-health/file_asset/file/234/Fluvoxamine.pdf) |
|  |  | Haloperidol | [Risperidone (hubble-live-assets.s3.eu-west-1.amazonaws.com)](https://hubble-live-assets.s3.eu-west-1.amazonaws.com/easy-health/file_asset/file/235/Haloperidol.pdf) |
|  |  | I am taking Carbamazepine | [Microsoft Word - easy read carbamazapine.doc (hubble-live-assets.s3.eu-west-1.amazonaws.com)](https://hubble-live-assets.s3.eu-west-1.amazonaws.com/easy-health/file_asset/file/20/im-taking-carbamarzepine.pdf) |
|  |  | I am taking Clozapine | [Microsoft Word - clozapine easy read.doc (hubble-live-assets.s3.eu-west-1.amazonaws.com)](https://hubble-live-assets.s3.eu-west-1.amazonaws.com/easy-health/file_asset/file/240/im-taking-clozapine.pdf) |
|  |  | I am taking Lamotrigine | <https://hubble-live-assets.s3.eu-west-1.amazonaws.com/easy-health/file_asset/file/28/im-taking-lamotragine.pdf> |
|  |  | I am taking Valproate | [Microsoft Word - valproate easy read.doc (hubble-live-assets.s3.eu-west-1.amazonaws.com)](https://hubble-live-assets.s3.eu-west-1.amazonaws.com/easy-health/file_asset/file/257/im-taking-valproate.pdf) |
|  |  | Lithium | [Risperidone (hubble-live-assets.s3.eu-west-1.amazonaws.com)](https://hubble-live-assets.s3.eu-west-1.amazonaws.com/easy-health/file_asset/file/236/Lithium.pdf) |
|  |  | Lofepramine | [Risperidone (hubble-live-assets.s3.eu-west-1.amazonaws.com)](https://hubble-live-assets.s3.eu-west-1.amazonaws.com/easy-health/file_asset/file/238/Lofepramine.pdf) |
|  |  | Lorazepam | <https://hubble-live-assets.s3.eu-west-1.amazonaws.com/easy-health/file_asset/file/239/Lorazepam.pdf> |
|  |  | Mirtazapine | [Risperidone (hubble-live-assets.s3.eu-west-1.amazonaws.com)](https://hubble-live-assets.s3.eu-west-1.amazonaws.com/easy-health/file_asset/file/241/Mirtazapine.pdf) |
|  |  | Paroxetine | [Risperidone (hubble-live-assets.s3.eu-west-1.amazonaws.com)](https://hubble-live-assets.s3.eu-west-1.amazonaws.com/easy-health/file_asset/file/247/Paroxetine.pdf) |
|  |  | Quetiapine | [Risperidone (hubble-live-assets.s3.eu-west-1.amazonaws.com)](https://hubble-live-assets.s3.eu-west-1.amazonaws.com/easy-health/file_asset/file/250/Quetiapine.pdf) |
|  |  | Sertraline | <https://hubble-live-assets.s3.eu-west-1.amazonaws.com/easy-health/file_asset/file/252/Sertraline.pdf> |
|  |  | Sulpiride | <https://hubble-live-assets.s3.eu-west-1.amazonaws.com/easy-health/file_asset/file/254/Sulpiride.pdf> |
|  |  | Topiramate | <https://hubble-live-assets.s3.eu-west-1.amazonaws.com/easy-health/file_asset/file/255/Topiramate.pdf> |
|  |  | Trazodone | [Risperidone (hubble-live-assets.s3.eu-west-1.amazonaws.com)](https://hubble-live-assets.s3.eu-west-1.amazonaws.com/easy-health/file_asset/file/256/Trazodone.pdf) |
|  |  | Trifluoperazine | <https://hubble-live-assets.s3.eu-west-1.amazonaws.com/easy-health/file_asset/file/258/Trifluoperazine.pdf> |
|  |  | Valproate | [Risperidone (hubble-live-assets.s3.eu-west-1.amazonaws.com)](https://hubble-live-assets.s3.eu-west-1.amazonaws.com/easy-health/file_asset/file/259/Valproate.pdf) |
|  |  | Venlafaxine | <https://hubble-live-assets.s3.eu-west-1.amazonaws.com/easy-health/file_asset/file/8/Venlafaxine.pdf> |
|  |  | Zuclopentixol | [Zuclopentixol \| Easy Health](https://www.easyhealth.org.uk/resources/286-zuclopentixol) |
|  |  | Choking Awareness | [Choking Awareness \| Easy Health](https://www.easyhealth.org.uk/resources/96-choking-awareness) |
|  |  | General anaesthetic for dental treatment - Easy read leaflet | <https://hubble-live-assets.s3.eu-west-1.amazonaws.com/easy-health/file_asset/file/1062/Anaesthetic.pdf> |
|  |  | Having fluoride varnish applied by the dental team - Easy read leaflet | <https://hubble-live-assets.s3.eu-west-1.amazonaws.com/easy-health/file_asset/file/1054/Varnish.pdf> |
|  |  | Intra-nasal sedation at the dentist - Easy read leaflet | [Print (hubble-live-assets.s3.eu-west-1.amazonaws.com)](https://hubble-live-assets.s3.eu-west-1.amazonaws.com/easy-health/file_asset/file/1060/Intra-nasal.pdf) |
|  |  | Intravenous sedation at the dentist | [Print (hubble-live-assets.s3.eu-west-1.amazonaws.com)](https://hubble-live-assets.s3.eu-west-1.amazonaws.com/easy-health/file_asset/file/1058/Intravenous.pdf) |
|  |  | Dental care (young patients) - NHS leaflet | <https://hubble-live-assets.s3.eu-west-1.amazonaws.com/easy-health/file_asset/file/1017/A4_Dental_2018.pdf> |
|  |  | Appointment communication cards | <https://hubble-live-assets.s3.eu-west-1.amazonaws.com/easy-health/file_asset/file/1008/CCG_My_Appointment_Easy-Read__1_.pdf> |
|  |  | Coming to have your  teeth checked | <https://hubble-live-assets.s3.eu-west-1.amazonaws.com/easy-health/file_asset/file/361/Coming-to-have-your-teeth-checked.pdf> |
|  |  | Going To The Dentist For Sedation | <https://hubble-live-assets.s3.eu-west-1.amazonaws.com/easy-health/file_asset/file/323/going-to-the-dentist-for-sedation.pdf> |
|  |  | Having an injection at the dentist | <https://hubble-live-assets.s3.eu-west-1.amazonaws.com/easy-health/file_asset/file/325/having_an_injection_at_the_dentist.pdf> |
|  |  | Keeping My Mouth Healthy Action Plan | <https://hubble-live-assets.s3.eu-west-1.amazonaws.com/easy-health/file_asset/file/327/Keeping-my-mouth-healthy-action-plan.pdf> |
|  |  | Keeping my Mouth HealthyChecklist | <https://hubble-live-assets.s3.eu-west-1.amazonaws.com/easy-health/file_asset/file/329/keeping-my-mouth-healthy-checklist.pdf> |
|  |  | When You Have Teeth Out | <https://hubble-live-assets.s3.eu-west-1.amazonaws.com/easy-health/file_asset/file/11/when_you_have_teeth_out.pdf> |
|  |  | Dysphagia | [Dysphagia \| Easy Health](https://www.easyhealth.org.uk/resources/167-dysphagia) |
|  |  | Diabetic eye screening | <https://hubble-live-assets.s3.eu-west-1.amazonaws.com/easy-health/file_asset/file/958/An_easy_guide_to_diabetic_eye_screening.pdf> |
|  |  | Screen use and your eyes | <https://hubble-live-assets.s3.eu-west-1.amazonaws.com/easy-health/file_asset/file/775/Screen_use_and_your_eyes_-_July_2021.pdf> |
|  |  | A Visit To The Optician | <https://www.easyhealth.org.uk/resources/198-a-visit-to-the-optician> |
|  |  | Eye Surgery Support Plan | <https://www.easyhealth.org.uk/resources/194-eye-surgery-support-plan> |
|  |  | Having An Eye Test | <https://hubble-live-assets.s3.eu-west-1.amazonaws.com/easy-health/file_asset/file/168/Having_an_eye_test__easy_read_.pdf> |
|  |  | Information For People With Learning Disabilities Who Have Visual Impairments | Information For People With Learning Disabilities Who Have Visual Impairments |
|  |  | Looking After Your Eyelids | <https://hubble-live-assets.s3.eu-west-1.amazonaws.com/easy-health/file_asset/file/172/looking_after_your_eyelids.pdf> |
|  |  | Diabetes Care: Taking Care of Your Eyes | <https://hubble-live-assets.s3.eu-west-1.amazonaws.com/easy-health/file_asset/file/131/Diabetes-care-taking-care-of-your-eyes.pdf> |
|  |  | Different Epileptic Fits | <https://hubble-live-assets.s3.eu-west-1.amazonaws.com/easy-health/file_asset/file/163/different_epileptic_fits.pdf> |
|  |  | Epilepsy | <https://hubble-live-assets.s3.eu-west-1.amazonaws.com/easy-health/file_asset/file/94/epilepsy.pdf> |
|  |  | Epilepsy Medicines | <https://hubble-live-assets.s3.eu-west-1.amazonaws.com/easy-health/file_asset/file/165/epilepsy_medicines.pdf> |
|  |  | Epilepsy: Staying Safe | <https://hubble-live-assets.s3.eu-west-1.amazonaws.com/easy-health/file_asset/file/99/Epilepsy-staying-safe.pdf> |
|  |  | Information About Epilepsy | <https://hubble-live-assets.s3.eu-west-1.amazonaws.com/easy-health/file_asset/file/3/information_about_epilepsy.pdf> |
|  |  | Valproate | <https://hubble-live-assets.s3.eu-west-1.amazonaws.com/easy-health/file_asset/file/259/Valproate.pdf> |
|  |  | Having a Hearing Test [Audiology Assessment] | [Having a Hearing Test [Audiology Assessment] \| Easy Health](https://www.easyhealth.org.uk/resources/having-a-hearing-test-audiology-assessment) |
|  |  | All About Tinnitus | <https://hubble-live-assets.s3.eu-west-1.amazonaws.com/easy-health/file_asset/file/93/All_about_tinnitus.pdf> |
|  |  | Audiology Information | <https://hubble-live-assets.s3.eu-west-1.amazonaws.com/easy-health/file_asset/file/147/audiology-information.pdf> |
|  |  | Dry Ear | <https://hubble-live-assets.s3.eu-west-1.amazonaws.com/easy-health/file_asset/file/148/dry_ear.pdf> |
|  |  | Getting Hearing Aids | <https://www.easyhealth.org.uk/resources/176-getting-hearing-aids> |
|  |  | Glue Ear and Grommets | <https://hubble-live-assets.s3.eu-west-1.amazonaws.com/easy-health/file_asset/file/149/glue-ear-grommets.pdf> |
|  |  | Having Trouble Hearing | <https://hubble-live-assets.s3.eu-west-1.amazonaws.com/easy-health/file_asset/file/150/having_trouble_hearing.pdf> |
|  |  | Mild Hearing Loss | [mild hearing loss 2.04.06.pub (hubble-live-assets.s3.eu-west-1.amazonaws.com)](https://hubble-live-assets.s3.eu-west-1.amazonaws.com/easy-health/file_asset/file/156/mild_hearing_loss.pdf) |
|  |  | Moderate Hearing Loss | <https://hubble-live-assets.s3.eu-west-1.amazonaws.com/easy-health/file_asset/file/157/moderate_hearing_loss.pdf> |
|  |  | My Ears and Hearing | <https://hubble-live-assets.s3.eu-west-1.amazonaws.com/easy-health/file_asset/file/155/My-ears-and-hearing.pdf> |
|  |  | Self Help for Tinnitus | <https://hubble-live-assets.s3.eu-west-1.amazonaws.com/easy-health/file_asset/file/152/Self_help_for_tinnitus.pdf> |
|  |  | Severe/Profound Hearing Loss | <https://hubble-live-assets.s3.eu-west-1.amazonaws.com/easy-health/file_asset/file/158/severe_or_profound_hearing_loss.pdf> |
|  |  | Tinnitus and Hearing Aids | <https://hubble-live-assets.s3.eu-west-1.amazonaws.com/easy-health/file_asset/file/151/Tinnitus_and_hearing_aids.pdf> |
|  |  | Tinnitus Services | [Tinnitus_services.pdf (hubble-live-assets.s3.eu-west-1.amazonaws.com)](https://hubble-live-assets.s3.eu-west-1.amazonaws.com/easy-health/file_asset/file/153/Tinnitus_services.pdf) |
|  |  | How To Look After Your Feet | [how-to-look-after-your-feet.pdf (hubble-live-assets.s3.eu-west-1.amazonaws.com)](https://hubble-live-assets.s3.eu-west-1.amazonaws.com/easy-health/file_asset/file/187/how-to-look-after-your-feet.pdf) |
|  |  | Visiting The Chiropodist | <https://hubble-live-assets.s3.eu-west-1.amazonaws.com/easy-health/file_asset/file/188/visiting-the-chiropodist.pdf> |
|  |  | Choosing If You Want To Have Your Gallbladder Out | <https://hubble-live-assets.s3.eu-west-1.amazonaws.com/easy-health/file_asset/file/189/Gallbladder_-_choosing__Eido_.pdf> |
|  |  | Gallbladder Removal | <https://hubble-live-assets.s3.eu-west-1.amazonaws.com/easy-health/file_asset/file/190/Gall-bladder-removal.pdf> |
|  |  | What is a Hiatus Hernia? | [What is a Hiatus Hernia? \| Easy Health](https://www.easyhealth.org.uk/resources/230-what-is-a-hiatus-hernia) |
|  |  | Look after your body - personal hygiene resource pack | <https://hubble-live-assets.s3.eu-west-1.amazonaws.com/easy-health/file_asset/file/1102/Personal_Care__Hygiene__1_.pdf> |
|  |  | Caring for our teeth - foods to have during or between meals | <https://hubble-live-assets.s3.eu-west-1.amazonaws.com/easy-health/file_asset/file/1021/Caring.pdf> |
|  |  | Healthy Eating | <https://hubble-live-assets.s3.eu-west-1.amazonaws.com/easy-health/file_asset/file/192/Healthy-Eating.pdf> |
|  |  | Planning your meals | <https://hubble-live-assets.s3.eu-west-1.amazonaws.com/easy-health/file_asset/file/194/Planning-Your-Meals.pdf> |
|  |  | Get Healthy! Live Longer! | [Get Healthy! Live Longer! \| Easy Health](https://www.easyhealth.org.uk/resources/219-get-healthy-live-longer) |
|  |  | A Week of Healthy Lunches | [a-week-of-healthy-lunches.pdf (hubble-live-assets.s3.eu-west-1.amazonaws.com)](https://hubble-live-assets.s3.eu-west-1.amazonaws.com/easy-health/file_asset/file/191/a-week-of-healthy-lunches.pdf) |
|  |  | Choosing What You Eat and Drink | <https://hubble-live-assets.s3.eu-west-1.amazonaws.com/easy-health/file_asset/file/193/Choosing-What-You-Eat-and-Drink.pdf> |
|  |  | Shape Up | <https://www.easyhealth.org.uk/resources/224-shape-up> |
|  |  | Stay Well This Winter 2023 | <https://hubble-live-assets.s3.eu-west-1.amazonaws.com/easy-health/file_asset/file/1005/221124_SWTW2022_HUHY_Stay_Well_Leaflet_EasyRead_WebAcc.pdf> |
|  |  | Keeping clean | <https://hubble-live-assets.s3.eu-west-1.amazonaws.com/easy-health/file_asset/file/1100/keeping_clean_easy_read.pdf> |
|  |  | Look after your body - personal hygiene resource pack | <https://hubble-live-assets.s3.eu-west-1.amazonaws.com/easy-health/file_asset/file/1102/Personal_Care__Hygiene__1_.pdf> |
|  |  | Cleaning Your Hands: Keeping Yourself and Others Free of Germs in Hospital | [NHS (hubble-live-assets.s3.eu-west-1.amazonaws.com)](https://hubble-live-assets.s3.eu-west-1.amazonaws.com/easy-health/file_asset/file/207/Cleaning_Your_Hands.pdf) |
|  |  | Germ Academy | [Germ Academy \| Easy Health](https://www.easyhealth.org.uk/resources/239-germ-academy) |
|  |  | How To Wash Your Hands | <https://hubble-live-assets.s3.eu-west-1.amazonaws.com/easy-health/file_asset/file/206/How-to-wash-your-hands.pdf> |
|  |  | Keep Clean | [keep_clean.pdf (hubble-live-assets.s3.eu-west-1.amazonaws.com)](https://hubble-live-assets.s3.eu-west-1.amazonaws.com/easy-health/file_asset/file/208/keep_clean.pdf) |
|  |  | Keep Healthy... Wash Your Hands | [Washing your hands Information (hubble-live-assets.s3.eu-west-1.amazonaws.com)](https://hubble-live-assets.s3.eu-west-1.amazonaws.com/easy-health/file_asset/file/205/Keep-healthy-wash-your-hands.pdf) |
|  |  | Checking your balls (testicles) | <https://hubble-live-assets.s3.eu-west-1.amazonaws.com/easy-health/file_asset/file/1455/Checking-your-balls-testicles.pdf> |
|  |  | Making a Decision about an Enlarged Prostate | [NHS Making a decision about heavy periods (hubble-live-assets.s3.eu-west-1.amazonaws.com)](https://hubble-live-assets.s3.eu-west-1.amazonaws.com/easy-health/file_asset/file/1389/Making_a_decision_about_an_Enlarged_Prostate-easy-read-version__Winton_Centre_.pdf) |
|  |  | My body, my choice | <https://hubble-live-assets.s3.eu-west-1.amazonaws.com/easy-health/file_asset/file/811/My_body__my_choice.pdf> |
|  |  | Men - Know About Your Prostate | [PROSTATE_easy_read.pdf (hubble-live-assets.s3.eu-west-1.amazonaws.com)](https://hubble-live-assets.s3.eu-west-1.amazonaws.com/easy-health/file_asset/file/764/PROSTATE_easy_read.pdf) |
|  |  | Testicular Awareness | [Microsoft Word - Testicular-Cancer-word.doc (hubble-live-assets.s3.eu-west-1.amazonaws.com)](https://hubble-live-assets.s3.eu-west-1.amazonaws.com/easy-health/file_asset/file/14/testicular-awareness-no-author.pdf) |
|  |  | Chlamydia Information for men | <https://hubble-live-assets.s3.eu-west-1.amazonaws.com/easy-health/file_asset/file/309/chlamydia_men.pdf> |
|  |  | How To Look After My Balls | <https://hubble-live-assets.s3.eu-west-1.amazonaws.com/easy-health/file_asset/file/311/How-to-look-after-my-balls.pdf> |
|  |  | Love, Sex and You | <https://hubble-live-assets.s3.eu-west-1.amazonaws.com/easy-health/file_asset/file/303/Love-sex-and-you.pdf> |
|  |  | Let’s talk about PUBERTY | [DSS Puberty output2.indd (hubble-live-assets.s3.eu-west-1.amazonaws.com)](https://hubble-live-assets.s3.eu-west-1.amazonaws.com/easy-health/file_asset/file/301/Lets_Talk_About_Puberty.pdf) |
|  |  | My Prostate: What Changes Should I Look Out For? | [My_prostate.pdf (hubble-live-assets.s3.eu-west-1.amazonaws.com)](https://hubble-live-assets.s3.eu-west-1.amazonaws.com/easy-health/file_asset/file/298/My_prostate.pdf) |
|  |  | How To Put On a Condom | <https://hubble-live-assets.s3.eu-west-1.amazonaws.com/easy-health/file_asset/file/121/How-to-put-on-a-condom.jpg> |
|  |  | What is Postural Care? | [What Is Postural Care? \| Easy Health](https://www.easyhealth.org.uk/resources/337-what-is-postural-care) |
|  |  | Physiotherapy for Adults with a Learning Disability | [Physiotherapy for Adults with a Learning Disability \| Easy Health](https://www.easyhealth.org.uk/resources/336-physiotherapy-for-adults-with-a-learning-disability) |
|  |  | I AM GROWING  INTO A WOMAN | <https://hubble-live-assets.s3.eu-west-1.amazonaws.com/easy-health/file_asset/file/300/I-am-growing-into-a-woman.pdf> |
|  |  | Growing up, gaining independence | <https://hubble-live-assets.s3.eu-west-1.amazonaws.com/easy-health/file_asset/file/1074/Growing_up_gaining_independence_ER0186_FINAL_Aug18__1_.pdf> |
|  |  | Having a PEG | [Having a PEG \| Easy Health](https://www.easyhealth.org.uk/resources/331-having-a-peg) |
|  |  | What is Procyclidine? | <https://hubble-live-assets.s3.eu-west-1.amazonaws.com/easy-health/file_asset/file/1344/Procyclidine-easy-read.pdf> |
|  |  | What is a Forceps birth? | <https://hubble-live-assets.s3.eu-west-1.amazonaws.com/easy-health/file_asset/file/1333/What-is-a-Forceps-birth_Easy-Read_LDN.pdf> |
|  |  | Help With Your Pain | <https://hubble-live-assets.s3.eu-west-1.amazonaws.com/easy-health/file_asset/file/285/Help-with-your-pain.pdf> |
|  |  | Patient Controlled Analgesia (PCA) | <https://hubble-live-assets.s3.eu-west-1.amazonaws.com/easy-health/file_asset/file/286/Patient-Controlled-Analgesia.pdf> |
|  |  | The Pain Is In My… | <https://hubble-live-assets.s3.eu-west-1.amazonaws.com/easy-health/file_asset/file/287/the_pain_is_in_my.pdf> |
|  |  | Epidurals For Pain | <https://hubble-live-assets.s3.eu-west-1.amazonaws.com/easy-health/file_asset/file/284/Epidurals-for-pain.pdf> |
|  |  | A Guide to Your CPAP | <https://www.england.nhs.uk/wp-content/uploads/2023/11/A-guide-to-your-CPAP-easy-read.pdf> |
|  |  | Getting a good night’s sleep | <https://hubble-live-assets.s3.eu-west-1.amazonaws.com/easy-health/file_asset/file/1076/Getting_a_good_nights_sleep_ER0367_FINAL_Jun20.pdf> |
|  |  | Sleep | [Sleep.pdf (hubble-live-assets.s3.eu-west-1.amazonaws.com)](https://hubble-live-assets.s3.eu-west-1.amazonaws.com/easy-health/file_asset/file/25/Sleep.pdf) |
|  |  | Keep Yourself Healthy: A Guide to Giving Up Smoking | [Keep Yourself Healthy: A Guide to Giving Up Smoking \| Easy Health](https://www.easyhealth.org.uk/resources/367-keep-yourself-healthy-a-guide-to-giving-up-smoking) |
|  |  | What is a stroke? | <https://www.easyhealth.org.uk/resources/what-is-a-stroke-video> |
|  |  | Act FAST If You Think Someone Is Having A Stroke | <https://hubble-live-assets.s3.eu-west-1.amazonaws.com/easy-health/file_asset/file/1068/230313_nhs-act-fast-stroke_EasyRead_Leaflet_WebAcc.pdf> |
|  |  | Stroke Thrombolysis Information Sheet | [Stroke Thrombolysis – Information Sheet (hubble-live-assets.s3.eu-west-1.amazonaws.com)](https://hubble-live-assets.s3.eu-west-1.amazonaws.com/easy-health/file_asset/file/319/Stroke-Thrombolysis-_-Information-Sheet.pdf) |
|  |  | Keep Yourself Healthy: Eating Well and Staying Fit – 1 Being Overweight | <https://hubble-live-assets.s3.eu-west-1.amazonaws.com/easy-health/file_asset/file/359/Keep-Yourself-Healthy-Eating-Well-and-Staying-Fit-1-Being-Overweight.pdf> |
|  |  | Overweight | <https://hubble-live-assets.s3.eu-west-1.amazonaws.com/easy-health/file_asset/file/331/overweight.pdf> |
|  |  | Weight | <https://hubble-live-assets.s3.eu-west-1.amazonaws.com/easy-health/file_asset/file/332/weight.pdf> |
|  |  | ADHD - Attention Deficit Hyperactivity Disorder | [ADHD - Attention Deficit Hyperactivity Disorder \| Easy Health](https://www.easyhealth.org.uk/resources/adhd-attention-deficit-hyperactivity-disorder) |
|  |  | Good mental health guide | <https://hubble-live-assets.s3.eu-west-1.amazonaws.com/easy-health/file_asset/file/981/Guide_to_good_mental_health_-_An_easy_read_guide_by_Learning_Disability_Wales.pdf> |
|  |  | The Stress Bucket | [hubble-live-assets.s3.eu-west-1.amazonaws.com/easy-health/file_asset/file/983/11.06.2020_StressBucket_easyread_v5-doxnqc.pdf](https://hubble-live-assets.s3.eu-west-1.amazonaws.com/easy-health/file_asset/file/983/11.06.2020_StressBucket_easyread_v5-doxnqc.pdf) |
|  |  | What is dementia? | <https://hubble-live-assets.s3.eu-west-1.amazonaws.com/easy-health/file_asset/file/931/what_is_dementia_er1.pdf> |
|  |  | Alcohol Relapse Prevention Plan | [view.officeapps.live.com/op/view.aspx?src=https%3A%2F%2Fhubble-live-assets.s3.eu-west-1.amazonaws.com%2Feasy-health%2Ffile_asset%2Ffile%2F830%2FCGL_Relapse_Prevention_Plan__1_.docx&wdOrigin=BROWSELINK](https://view.officeapps.live.com/op/view.aspx?src=https%3A%2F%2Fhubble-live-assets.s3.eu-west-1.amazonaws.com%2Feasy-health%2Ffile_asset%2Ffile%2F830%2FCGL_Relapse_Prevention_Plan__1_.docx&wdOrigin=BROWSELINK) |
|  |  | Mental Health Patient Safety Plan | [MH_Patient_Safety_Plan__1_.docx (live.com)](https://view.officeapps.live.com/op/view.aspx?src=https%3A%2F%2Fhubble-live-assets.s3.eu-west-1.amazonaws.com%2Feasy-health%2Ffile_asset%2Ffile%2F832%2FMH_Patient_Safety_Plan__1_.docx&wdOrigin=BROWSELINK) |
|  |  | Mental Health: Anxiety, Depression, and Schizophrenia | [Mental-Health.pdf (hubble-live-assets.s3.eu-west-1.amazonaws.com)](https://hubble-live-assets.s3.eu-west-1.amazonaws.com/easy-health/file_asset/file/360/Mental-Health.pdf) |
|  |  | A guide to writing your recovery plan | [A guide to writing your recovery plan - easy read (hubble-live-assets.s3.eu-west-1.amazonaws.com)](https://hubble-live-assets.s3.eu-west-1.amazonaws.com/easy-health/file_asset/file/263/A-guide-to-writing-your-recovery-plan.pdf) |
|  |  | Anxiety | [Anxiety final (hubble-live-assets.s3.eu-west-1.amazonaws.com)](https://hubble-live-assets.s3.eu-west-1.amazonaws.com/easy-health/file_asset/file/265/anxiety-st-georges.pdf) |
|  |  | How are you today? | [How-are-you-today.pdf (hubble-live-assets.s3.eu-west-1.amazonaws.com)](https://hubble-live-assets.s3.eu-west-1.amazonaws.com/easy-health/file_asset/file/269/How-are-you-today.pdf) |
|  |  | Looking After Your Mental Health | <https://hubble-live-assets.s3.eu-west-1.amazonaws.com/easy-health/file_asset/file/270/looking_after_your_mental_health.pdf> |
|  |  | Making an appeal | [Microsoft Word - appeal section 2 (hubble-live-assets.s3.eu-west-1.amazonaws.com)](https://hubble-live-assets.s3.eu-west-1.amazonaws.com/easy-health/file_asset/file/266/appeal_mental_health_act_section_2.pdf) |
|  |  | Post-Traumatic Stress Disorder (PTSD) | [Publication PTSDeasyreadleaflet (hubble-live-assets.s3.eu-west-1.amazonaws.com)](https://hubble-live-assets.s3.eu-west-1.amazonaws.com/easy-health/file_asset/file/271/post-traumatic-stress-disorder-PTSD.pdf) |
|  |  | Depression | [hubble-live-assets.s3.eu-west-1.amazonaws.com/easy-health/file_asset/file/130/Depression.pdf](https://hubble-live-assets.s3.eu-west-1.amazonaws.com/easy-health/file_asset/file/130/Depression.pdf) |
|  |  | Anger | [untitled (hubble-live-assets.s3.eu-west-1.amazonaws.com)](https://hubble-live-assets.s3.eu-west-1.amazonaws.com/easy-health/file_asset/file/47/Anger.pdf) |
|  |  | Challenging Behaviour | [PowerPoint Presentation (hubble-live-assets.s3.eu-west-1.amazonaws.com)](https://hubble-live-assets.s3.eu-west-1.amazonaws.com/easy-health/file_asset/file/49/Challenging-Behaviour.pdf) |
|  |  | Anxiety | [Anxiety.pdf (hubble-live-assets.s3.eu-west-1.amazonaws.com)](https://hubble-live-assets.s3.eu-west-1.amazonaws.com/easy-health/file_asset/file/45/Anxiety.pdf) |
|  |  |  |  |
|  |  | Supporting People with Disabilities Coping With Grief and Loss | [Supporting People with Disabilities Coping With Grief and Loss \| Easy Health](https://www.easyhealth.org.uk/resources/163-supporting-people-with-disabilities-coping-with-grief-and-loss) |
|  |  | Seeing the Psychologist for a Cognitive Assessment | [Seeing the Psychologist for a Cognitive Assessment \| Easy Health](https://www.easyhealth.org.uk/resources/339-seeing-the-psychologist-for-a-cognitive-assessment) |
|  |  | Consent and capacity | <https://hubble-live-assets.s3.eu-west-1.amazonaws.com/easy-health/file_asset/file/117/Consent-and-capacity.pdf> |
|  |  | Making choices about your health care. | [Making-choices.pdf (hubble-live-assets.s3.eu-west-1.amazonaws.com)](https://hubble-live-assets.s3.eu-west-1.amazonaws.com/easy-health/file_asset/file/105/Making-choices.pdf) |
|  |  | Mental Capacity Act An Easy Read Guide 2005 | [Mental Capacity Act 2005: An easy read guide (hubble-live-assets.s3.eu-west-1.amazonaws.com)](https://hubble-live-assets.s3.eu-west-1.amazonaws.com/easy-health/file_asset/file/103/Mental_Capacity_Act_2005_easy_read_guide.pdf) |
|  |  | The Mental Capacity Act An easy read summary of the Mental Capacity Act 2005. | [Mental Capacity Act 2005 Summary - easy read (hubble-live-assets.s3.eu-west-1.amazonaws.com)](https://hubble-live-assets.s3.eu-west-1.amazonaws.com/easy-health/file_asset/file/101/mental_capacity_act.pdf) |
|  |  | What to do if you are not happy with your healthcare | [What To Do If You Are Not Happy With Your Healthcare \| Easy Health](https://www.easyhealth.org.uk/resources/99-what-to-do-if-you-are-not-happy-with-your-healthcare) |
|  |  | Consent: Making decisions about your  health care and treatment | <https://hubble-live-assets.s3.eu-west-1.amazonaws.com/easy-health/file_asset/file/118/Consent.pdf> |
|  |  | Consent: A Guide For People With Learning Disabilities | Consent: A Guide For People With Learning Disabilities |
|  |  | Going to the Doctor | [Going to the Doctor \| Easy Health](https://www.easyhealth.org.uk/resources/159-going-to-the-doctor) |
|  |  | Questions To Ask When You Go To The Doctor or To The Hospital | [Questions To Ask When You Go To The Doctor or To The Hospital \| Easy Health](https://www.easyhealth.org.uk/resources/157-questions-to-ask-when-you-go-to-the-doctor-or-to-the-hospital) |
|  |  | Discharged From Hospital Against Medical Advice | [Discharge against medical advice 260111 (hubble-live-assets.s3.eu-west-1.amazonaws.com)](https://hubble-live-assets.s3.eu-west-1.amazonaws.com/easy-health/file_asset/file/268/discharged-from-hospital-against-medical-advice.pdf) |
|  |  | You Are Pregnant: Your Choices | [You Are Pregnant: Your Choices](https://www.easyhealth.org.uk/resources/243-you-are-pregnant-your-choices) |
|  |  | What is an abortion? | [What_is_an_abortion_-_master.pdf](https://hubble-live-assets.s3.eu-west-1.amazonaws.com/easy-health/file_asset/file/174/What_is_an_abortion_-_master.pdf) |
|  |  | Breast | [Easy Health](https://www.easyhealth.org.uk/resources/category/33-breast) |
|  |  | Signs and symptoms of menopause | [02. menopause signs and symptoms (November-2021) ER (hubble-live-assets.s3.eu-west-1.amazonaws.com)](https://hubble-live-assets.s3.eu-west-1.amazonaws.com/easy-health/file_asset/file/1327/menopause-signs-and-symptoms-.pdf) |
|  |  | Periods | [Easy Health](https://www.easyhealth.org.uk/resources/category/119-periods) |
|  |  | Menopause | [Easy Health](https://www.easyhealth.org.uk/resources/category/118-menopause) |
|  |  | Pregnancy | <https://www.easyhealth.org.uk/resources/category/112-pregnancy> |
|  |  | Anaesthetic | [Easy Health](https://www.easyhealth.org.uk/resources/category/19-anaesthetic) |
|  |  | Abdominal Aortic Aneurysms (AAA) screening | [Abdominal Aortic Aneurysms (AAA) screening \| Easy Health](https://www.easyhealth.org.uk/resources/abdominal-aortic-aneurysms) |
|  |  | Annual Health Check | [Easy Health](https://www.easyhealth.org.uk/resources/category/105-annual-health-check) |
|  |  | Easy guide to screening for Down’s syndrome, Edwards’ syndrome and Patau’s syndrome (pregnancy) | [Easy guide to screening for Down’s syndrome, Edwards’ syndrome and Patau’s syndrome (pregnancy) \| Easy Health](https://www.easyhealth.org.uk/resources/easy-guide-to-screening-for-down-s-syndrome-edwards-syndrome-and-patau-s-syndrome-pregnancy) |
|  |  | Easy guide to screening tests for sickle cell disease and thalassaemia when you are pregnant | [Easy guide to screening tests for sickle cell disease and thalassaemia when you are pregnant \| Easy Health](https://www.easyhealth.org.uk/resources/easy-guide-to-screening-tests-for-sickle-cell-disease-and-thalassaemia-when-you-are-pregnant) |
|  |  | Bone Marrow Biopsy | [Bone Marrow Biopsy \| Easy Health](https://www.easyhealth.org.uk/resources/390-bone-marrow-biopsy) |
|  |  | Biopsy | [Biopsy \| Easy Health](https://www.easyhealth.org.uk/resources/389-biopsy) |
|  |  | Blood Test | [Easy Health](https://www.easyhealth.org.uk/resources/category/28-blood-test) |
|  |  | CT Scan | [Easy Health](https://www.easyhealth.org.uk/resources/category/154-ct-scan) |
|  |  | Endoscopy | [Endoscopy \| Easy Health](https://www.easyhealth.org.uk/resources/399-endoscopy) |
|  |  | EEG | [Easy Health](https://www.easyhealth.org.uk/resources/category/96-eeg) |
|  |  | Having a chest x-ray | https://hubble-live-assets.s3.eu-west-1.amazonaws.com/easy-health/file_asset/file/345/Having-a-Chest-X-Ray.pdf |
|  |  | Abdominal Cancer | [Easy Health](https://www.easyhealth.org.uk/resources/category/162-abdominal-cancer) |
|  |  | Osteoporosis | [Osteoporosis \| Easy Health](https://www.easyhealth.org.uk/resources/321-osteoporosis) |
|  |  | Cancer | [Easy Health](https://www.easyhealth.org.uk/resources/category/7-cancer) |
|  |  | Easy Read - Diagnosed with primary breast cancer | <https://hubble-live-assets.s3.eu-west-1.amazonaws.com/easy-health/file_asset/file/1263/easy_read_-_bcn255_diagnosed_with_primary_breast_cancer.pdf> |
|  |  | HPV vaccination - Easy read guide | <https://hubble-live-assets.s3.eu-west-1.amazonaws.com/easy-health/file_asset/file/1146/UKHSA_12378_easy-read_HPV_vaccine_information.pdf> |
|  |  | Stay healthy - Be safe in the sun - easy read (Macmillan) | <https://hubble-live-assets.s3.eu-west-1.amazonaws.com/easy-health/file_asset/file/1078/MAC16335_ER_E06_Be%2BSafe%2Bin%2Bthe%2BSun.pdf> |
|  |  | Diabetic eye screening: easy read invitation letter template (NHS) | <https://www.easyhealth.org.uk/resources/diabetic-eye-screening-easy-read-invitation-letter-template-nhs> |
|  |  | The end of life - Easy read leaflet | <https://hubble-live-assets.s3.eu-west-1.amazonaws.com/easy-health/file_asset/file/1064/MAC16343_ER_E04_The_end_of_life.pdf> |
|  |  | Spotting signs of Lung Cancer - poster from the NHS | <https://hubble-live-assets.s3.eu-west-1.amazonaws.com/easy-health/file_asset/file/997/lung-cancer-symptoms_easy-read_web-print.pdf> |
|  |  | Breast screening normal result easy read letter template - NHS | <https://www.easyhealth.org.uk/resources/nhs-breast-screening-normal-result-easy-read-letter-template> |
|  |  | Breast screening recall easy read letter template - NHS | <https://www.easyhealth.org.uk/resources/nhs-breast-screening-recall-to-assessment-easy-read-letter-template> |
|  |  | Breat screening technical recall easy read letter template - NHS | <https://www.easyhealth.org.uk/resources/nhs-breast-screening-technical-recall-easy-read-letter-template#google_vignette> |
|  |  | Cervical screening: easy read invitation letter template - NHS | <https://www.easyhealth.org.uk/resources/cervical-screening-easy-read-invitation-letter-template> |
|  |  | Breast screening invitation easy read letter template | <https://www.easyhealth.org.uk/resources/nhs-breast-screening-invitation-easy-read-letter-template> |
|  |  | Bowel cancer screening easy read invitation letter content | <https://www.easyhealth.org.uk/resources/bowel-cancer-screening-easy-read-invitation-letter-content> |
|  |  | Bowel cancer screening results letter (A183) - more tests needed - Easy read letter | <https://www.easyhealth.org.uk/resources/bowel-cancer-screening-a183-more-tests-needed-easy-read-letter-content> |
|  |  | Bowel cancer screening S2 no further tests required eady read letter content | <https://www.easyhealth.org.uk/resources/bowel-cancer-screening-s2-no-further-tests-required-easy-read-letter-content> |
|  |  | [Bowel cancer screening S2 previous cancer no further tests required easy read letter content](https://www.easyhealth.org.uk/resources/bowel-cancer-screening-s2-previous-cancer-no-further-tests-required-easy-read-letter-content) | <https://www.easyhealth.org.uk/resources/bowel-cancer-screening-s2-previous-cancer-no-further-tests-required-easy-read-letter-content> |
|  |  | [Bowel cancer screening invite letter (easy read)](https://www.easyhealth.org.uk/resources/bowel-cancer-screening_invite_letter_easy_read_nhs) | <https://www.easyhealth.org.uk/resources/bowel-cancer-screening_invite_letter_easy_read_nhs> |
|  |  | Breast screening | <https://hubble-live-assets.s3.eu-west-1.amazonaws.com/easy-health/file_asset/file/898/Easy_guide_to_breast_screening.pdf> |
|  |  | What is Mouth Cancer? | <https://hubble-live-assets.s3.eu-west-1.amazonaws.com/easy-health/file_asset/file/356/What_is_mouth_cancer_-_Easy_Read.pdf> |
|  |  | Cancer Screenings for People with Learning Disabilities | <https://www.easyhealth.org.uk/resources/353-cancer-screenings-for-people-with-learning-disabilities> |
|  |  | How Macmillan Cancer Support Can Help You | <https://hubble-live-assets.s3.eu-west-1.amazonaws.com/easy-health/file_asset/file/106/how-macmillan-can-help-you.pdf> |
|  |  | Keep Yourself Healthy: A Guide to Having a Smear Test | <https://hubble-live-assets.s3.eu-west-1.amazonaws.com/easy-health/file_asset/file/97/Keep-Yourself-Healthy-A-Guide-to-Having-a-Smear-Test.pdf> |
|  |  | Keep Yourself Healthy: Do I Need a Smear Test? | <https://hubble-live-assets.s3.eu-west-1.amazonaws.com/easy-health/file_asset/file/98/Keep-Yourself-Healthy-Do-i-Need-a-Smear-Test.pdf> |
|  |  | Prostate Cancer | <https://hubble-live-assets.s3.eu-west-1.amazonaws.com/easy-health/file_asset/file/83/Prostate-Cancer-South-Staffordshire.pdf> |
|  |  | Radiotherapy | <https://hubble-live-assets.s3.eu-west-1.amazonaws.com/easy-health/file_asset/file/85/radiotherapy.pdf> |
|  |  | Skin Cancer | <https://hubble-live-assets.s3.eu-west-1.amazonaws.com/easy-health/file_asset/file/86/skin-cancer.pdf> |
|  |  | Skin Cancer and Sun Safety | <https://hubble-live-assets.s3.eu-west-1.amazonaws.com/easy-health/file_asset/file/1/Skin-Cancer-Sun-Safety.pdf> |
|  |  | Sun Beds | <https://hubble-live-assets.s3.eu-west-1.amazonaws.com/easy-health/file_asset/file/84/Sun-Beds.pdf> |
|  |  | Telling You About Oesophageal Cancer | <https://hubble-live-assets.s3.eu-west-1.amazonaws.com/easy-health/file_asset/file/87/Telling-you-about-Oesophageal-Cancer.pdf> |
|  |  | Telling You About Stomach Cancer | <https://hubble-live-assets.s3.eu-west-1.amazonaws.com/easy-health/file_asset/file/88/Telling-you-about-Stomach-Cancer.pdf> |
|  |  | The Smear Test Film | <https://www.easyhealth.org.uk/resources/80-the-smear-test-film> |
|  |  | Treatment for Cancer | <https://hubble-live-assets.s3.eu-west-1.amazonaws.com/easy-health/file_asset/file/92/Treatment-for-cancer.pdf> |
|  |  | Breast Cancer and Breast Screening | <https://hubble-live-assets.s3.eu-west-1.amazonaws.com/easy-health/file_asset/file/74/Breast-Cancer-Breast-Screening.pdf> |
|  |  | Cervical Cancer and Cervical Screening | <https://hubble-live-assets.s3.eu-west-1.amazonaws.com/easy-health/file_asset/file/75/Cervical-Cancer-Cervical-Screening.pdf> |
|  |  | Chemotherapy | <https://hubble-live-assets.s3.eu-west-1.amazonaws.com/easy-health/file_asset/file/77/chemotherapy.pdf> |
|  |  | Lung Cancer | <https://hubble-live-assets.s3.eu-west-1.amazonaws.com/easy-health/file_asset/file/79/Lung-Cancer.pdf> |
|  |  | Ovarian Cancer | <https://hubble-live-assets.s3.eu-west-1.amazonaws.com/easy-health/file_asset/file/80/Ovarian-Cancer.pdf> |
|  |  | Prostate Cancer | <https://hubble-live-assets.s3.eu-west-1.amazonaws.com/easy-health/file_asset/file/81/Prostate-Cancer.pdf> |
|  |  | About the Cervical Cancer Vaccination | <https://hubble-live-assets.s3.eu-west-1.amazonaws.com/easy-health/file_asset/file/69/About-the-Cervical-Cancer-Vaccination.pdf> |
|  |  | An Easy Guide to Breast Screening | <https://hubble-live-assets.s3.eu-west-1.amazonaws.com/easy-health/file_asset/file/66/easy-guide-breast-screening.pdf> |
|  |  | Be Breast Aware | <https://hubble-live-assets.s3.eu-west-1.amazonaws.com/easy-health/file_asset/file/63/Be_Breast_Aware.pdf> |
|  |  | Breat Cancer and How To Spot it |  |
|  |  | Do The Test |  |
|  |  | Having A Mammogram |  |
|  |  | Keep Yourself Healthy: A Guide to Examining Your Breasts |  |
|  |  | My Boobs and Me |  |
|  |  | Spotting Signs of Abdominal Cancer |  |
|  |  | Taking Care of Your Breasts |  |
|  |  | Cervical Screening Test |  |
|  |  | Spotting sings of cancer |  |
|  |  | Lung and Breathing Problems (COPD) | [Lung and Breathing Problems (COPD) \| Easy Health](https://www.easyhealth.org.uk/resources/411-lung-and-breathing-problems-copd) |
|  |  | COVID-19 | [Easy Health](https://www.easyhealth.org.uk/resources/category/10-covid-19) |
|  |  | Easy Read Guide to the Covid-19 Booster Vaccination | <https://hubble-live-assets.s3.eu-west-1.amazonaws.com/easy-health/file_asset/file/819/UKHSA-12222-COVID-19-booster-vaccination-easy-read.pdf> |
|  |  | Love in Lockdown | <https://hubble-live-assets.s3.eu-west-1.amazonaws.com/easy-health/file_asset/file/350/Love%2Bin%2BLockdown%2B-%2Bweb.pdf> |
|  |  | Having Your Oxygen Levels Checked | <https://hubble-live-assets.s3.eu-west-1.amazonaws.com/easy-health/file_asset/file/349/Oxygen-levels.pdf> |
|  |  | Having a Vaccine for Coronavirus | <https://hubble-live-assets.s3.eu-west-1.amazonaws.com/easy-health/file_asset/file/200/Having%2Ba%2BVaccine%2Bfor%2BCoronavirus.pdf> |
|  |  | Let's Talk About... When Someone is Ill or Dies From Coronavirus | <https://hubble-live-assets.s3.eu-west-1.amazonaws.com/easy-health/file_asset/file/146/Lets%2Btalk%2Babout%2Bwhen%2Bsomeone%2Bdies%2Bfrom%2BCOVID.pdf> |
|  |  | A guide to your COVID-19 vaccination | <https://hubble-live-assets.s3.eu-west-1.amazonaws.com/easy-health/file_asset/file/21/PHE_11843_Covid-19_Easy-read_leaflet.pdf> |
|  |  | Coronavirus (Covid-19): Getting Better in Hospital | <https://hubble-live-assets.s3.eu-west-1.amazonaws.com/easy-health/file_asset/file/114/Hospital-and-coronavirus.pdf> |
|  |  | Coronavirus Vaccine in a Hospital | <https://hubble-live-assets.s3.eu-west-1.amazonaws.com/easy-health/file_asset/file/112/Coronavirus-Vaccine-HOSPITAL-FEB21.pdf> |
|  |  | Covid-19 Hospital Summary | <https://hubble-live-assets.s3.eu-west-1.amazonaws.com/easy-health/file_asset/file/104/CV19-Hospital-summary.pdf> |
|  |  | Covid-19 Swab Test | <https://hubble-live-assets.s3.eu-west-1.amazonaws.com/easy-health/file_asset/file/113/Covid-19_Swab_test.pdf> |
|  |  | Having a Test for Coronavirus | <https://hubble-live-assets.s3.eu-west-1.amazonaws.com/easy-health/file_asset/file/111/Having%2Ba%2BTest%2Bfor%2BCoronavirus.pdf> |
|  |  | Using a Pulse Oximeter to Check You Are OK | <https://hubble-live-assets.s3.eu-west-1.amazonaws.com/easy-health/file_asset/file/82/Pulse-Oximeter-Easy-Read-final-online-v4.pdf> |
|  |  | Dementia | [Easy Health](https://www.easyhealth.org.uk/resources/category/48-dementia) |
|  |  | Dementia | <https://hubble-live-assets.s3.eu-west-1.amazonaws.com/easy-health/file_asset/file/128/Dementia.pdf> |
|  |  | NHS Health Check: Dementia | <https://hubble-live-assets.s3.eu-west-1.amazonaws.com/easy-health/file_asset/file/126/NHS_Health_Check_Dementia_Leaflet.pdf> |
|  |  | Tablets For People Who Have Dementia | <https://hubble-live-assets.s3.eu-west-1.amazonaws.com/easy-health/file_asset/file/129/Tablets-for-people-who-have-Dementia.pdf> |
|  |  | What is Dementia? | <https://hubble-live-assets.s3.eu-west-1.amazonaws.com/easy-health/file_asset/file/127/what-is-dementia-Easy-Read.pdf> |
|  |  | Diabetes | [Easy Health](https://www.easyhealth.org.uk/resources/category/82-diabetes) |
|  |  | Diabetes | <https://hubble-live-assets.s3.eu-west-1.amazonaws.com/easy-health/file_asset/file/133/diabetes.pdf> |
|  |  | Diabetes ABC Advice | <https://hubble-live-assets.s3.eu-west-1.amazonaws.com/easy-health/file_asset/file/132/Diabetes-ABC.pdf> |
|  |  | Diana has Diabetes | <https://www.easyhealth.org.uk/resources/154-diana-has-diabetes> |
|  |  | Getting Started If You Have Diabetes | <https://hubble-live-assets.s3.eu-west-1.amazonaws.com/easy-health/file_asset/file/134/getting-Started-with-diabetes.pdf> |
|  |  | Jo's Story | <https://www.easyhealth.org.uk/resources/153-jo-s-story> |
|  |  | What To Do When You Have Type 2 Diabetes | <https://hubble-live-assets.s3.eu-west-1.amazonaws.com/easy-health/file_asset/file/135/What-to-do-when-you-have-Type-2-diabetes.pdf> |
|  |  | Heart Disease | [Easy Health](https://www.easyhealth.org.uk/resources/category/107-heart-disease) |
|  |  | Don't ignore the signs of a heart attack - poster/flyer from the NHS | <https://hubble-live-assets.s3.eu-west-1.amazonaws.com/easy-health/file_asset/file/1253/231111_HeartAttack_EasyRead-Poster-Accessible.pdf> |
|  |  | Live With a Healthy Heart | <https://www.easyhealth.org.uk/resources/322-live-with-a-healthy-heart> |
|  |  | Coronary Heart Disease | <https://hubble-live-assets.s3.eu-west-1.amazonaws.com/easy-health/file_asset/file/212/Coronary_Heart_Disease.pdf> |
|  |  | A Guide To Having A Healthy Heart | <https://hubble-live-assets.s3.eu-west-1.amazonaws.com/easy-health/file_asset/file/185/A-Guide-To-Having-A-Healthy-Heart.pdf> |
|  |  | Coronary Heart Disease | <https://hubble-live-assets.s3.eu-west-1.amazonaws.com/easy-health/file_asset/file/195/Coronary-Heart-Disease.pdf> |
|  |  | Making Life Better For Adults With Congenital Heart Disease | <https://hubble-live-assets.s3.eu-west-1.amazonaws.com/easy-health/file_asset/file/196/making-life-better-for-adults-with-congenital-heart-disease.pdf> |
|  |  | Your Heart Surgery and Recovery | <https://hubble-live-assets.s3.eu-west-1.amazonaws.com/easy-health/file_asset/file/197/Your-Heart-Surgery-and-Recovery.pdf> |
|  |  | Parkinson's Disease | [Easy Health](https://www.easyhealth.org.uk/resources/category/127-parkinsons-disease) |
|  |  | What is Procyclidine? | <https://hubble-live-assets.s3.eu-west-1.amazonaws.com/easy-health/file_asset/file/1344/Procyclidine-easy-read.pdf> |
|  |  | Parkinson's Disease | <https://hubble-live-assets.s3.eu-west-1.amazonaws.com/easy-health/file_asset/file/291/parkinsons-disease.pdf> |
|  |  | Parkinson's Disease Information Leaflet | <https://hubble-live-assets.s3.eu-west-1.amazonaws.com/easy-health/file_asset/file/290/Parkinsons-Information-leaflet.pdf> |
|  |  | Sepsis | [Easy Health](https://www.easyhealth.org.uk/resources/category/140-sepsis) |
|  |  | After you've had Sepsis | <https://hubble-live-assets.s3.eu-west-1.amazonaws.com/easy-health/file_asset/file/1478/easy-read-problems-after-sepsis-.pdf> |
|  |  | How to avoid sepsis | <https://hubble-live-assets.s3.eu-west-1.amazonaws.com/easy-health/file_asset/file/1476/easy-read-how-to-avoid-sepsis.pdf> |
|  |  | Signs of Sepsis and What to Do | <https://hubble-live-assets.s3.eu-west-1.amazonaws.com/easy-health/file_asset/file/1474/easy-read-signs-of-sepsis-and-what-to-do.pdf> |
|  |  | DiaDi | <https://hubble-live-assets.s3.eu-west-1.amazonaws.com/easy-health/file_asset/file/936/What_is_Sepsis_-_Easy_Read.pdf> |
|  |  | Sepsis Easy Read | <https://hubble-live-assets.s3.eu-west-1.amazonaws.com/easy-health/file_asset/file/307/Sepsis-Easy-Read-version-ILDP-Nov-18.pdf> |
|  |  | Sepsis Plain English | <https://hubble-live-assets.s3.eu-west-1.amazonaws.com/easy-health/file_asset/file/308/Sepsis-Plain-English-Version-ILDP-Oct-18.pdf> |
|  |  | Sexual Health | [Easy Health](https://www.easyhealth.org.uk/resources/category/137-sexual-health) |
|  |  | Menstruation | <https://hubble-live-assets.s3.eu-west-1.amazonaws.com/easy-health/file_asset/file/1469/Menstruation_Easy_Read.pdf> |
|  |  | My Prostate, What Changes should I look out for? | <https://hubble-live-assets.s3.eu-west-1.amazonaws.com/easy-health/file_asset/file/1467/find-out-about-your-prostate_ifm__Easy_Read_.pdf> |
|  |  | Keeping your Bladder Healthy - easy read [G.O.S.H] | <https://hubble-live-assets.s3.eu-west-1.amazonaws.com/easy-health/file_asset/file/1402/Keeping_your_bladder_healthy_-_easy_read__G.O.S.H_.pdf> |
|  |  | Easy Read - How will my baby be born? | <https://hubble-live-assets.s3.eu-west-1.amazonaws.com/easy-health/file_asset/file/1331/How-will-my-baby-be-born_Easy-Read_LDN.pdf> |
|  |  | Easy Read - What is a Forceps birth? | <https://hubble-live-assets.s3.eu-west-1.amazonaws.com/easy-health/file_asset/file/1333/What-is-a-Forceps-birth_Easy-Read_LDN.pdf> |
|  |  | Easy Read - Signs and Symptoms of Menopause | <https://hubble-live-assets.s3.eu-west-1.amazonaws.com/easy-health/file_asset/file/1327/menopause-signs-and-symptoms-.pdf> |
|  |  | Easy Read - What is menopause? | <https://hubble-live-assets.s3.eu-west-1.amazonaws.com/easy-health/file_asset/file/1329/Menopause.pdf> |
|  |  | Caesarean birth - Easy read resource | <https://hubble-live-assets.s3.eu-west-1.amazonaws.com/easy-health/file_asset/file/1166/Ceasarean-easy_read.pdf> |
|  |  | Information leaflet to support consent form for Caesarean section (C-Section) - easy read | <https://hubble-live-assets.s3.eu-west-1.amazonaws.com/easy-health/file_asset/file/1169/C-section_consent.pdf> |
|  |  | Having a smear test - easy read | <https://hubble-live-assets.s3.eu-west-1.amazonaws.com/easy-health/file_asset/file/1142/easyread_having_smear_test_compressed_1-1__1_.pdf> |
|  |  | LGBTQ+ An easy read guide - Change & MESMAC | <https://hubble-live-assets.s3.eu-west-1.amazonaws.com/easy-health/file_asset/file/1036/LGBTQ-booklet-18-05-20.pdf> |
|  |  | Transgender - easy read guide - Choice Support & Change leaflet | <https://hubble-live-assets.s3.eu-west-1.amazonaws.com/easy-health/file_asset/file/1038/Transgender-easy-read-guide-For-Web.pdf> |
|  |  | 5 HIV facts leaflet | <https://hubble-live-assets.s3.eu-west-1.amazonaws.com/easy-health/file_asset/file/961/HIV-easy_read.pdf> |
|  |  | HIV - AIDS information guide | <https://hubble-live-assets.s3.eu-west-1.amazonaws.com/easy-health/file_asset/file/963/HIV-AIDS-Apr-18.pdf> |
|  |  | Easy guide to screening tests for sickle cell diseases and thalasseamia when you are pregnant | <https://hubble-live-assets.s3.eu-west-1.amazonaws.com/easy-health/file_asset/file/950/Easy_guide_to_screening_tests_for_sickle_cell_disease_and_thalassaemia_when_you_are_pregnant_easy_read_SCT_section_June_2020.pdf> |
|  |  | Screening for hepatitis B, HIV and syphilis when you are pregnant | <https://hubble-live-assets.s3.eu-west-1.amazonaws.com/easy-health/file_asset/file/948/Screening_for_hepatitis_B__HIV_and_syphilis_when_you_are_pregnant_easy_read_IDPS_section_June_2020.pdf> |
|  |  | Let's Talk About Sex! | <https://hubble-live-assets.s3.eu-west-1.amazonaws.com/easy-health/file_asset/file/813/Let_s_talk_about_sex___1_.pdf> |
|  |  | Me and My Body | <https://hubble-live-assets.s3.eu-west-1.amazonaws.com/easy-health/file_asset/file/809/Me_and_my_body.pdf> |
|  |  | My Body My Choice | <https://hubble-live-assets.s3.eu-west-1.amazonaws.com/easy-health/file_asset/file/811/My_body__my_choice.pdf> |
|  |  | Chlamydia: Information for Men | <https://hubble-live-assets.s3.eu-west-1.amazonaws.com/easy-health/file_asset/file/309/chlamydia_men.pdf> |
|  |  | Chlamydia: Information for Women | <https://hubble-live-assets.s3.eu-west-1.amazonaws.com/easy-health/file_asset/file/310/chlamydia_women.pdf> |
|  |  | Sexually Transmitted Infections (STIs) | <https://hubble-live-assets.s3.eu-west-1.amazonaws.com/easy-health/file_asset/file/312/SexuallyTransmittedInfections.pdf> |
|  |  | What Is Inside? | <https://hubble-live-assets.s3.eu-west-1.amazonaws.com/easy-health/file_asset/file/5/what_is_inside.pdf> |
|  |  | Your Private Parts | <https://hubble-live-assets.s3.eu-west-1.amazonaws.com/easy-health/file_asset/file/23/your_private_parts.pdf> |
|  |  | Love, Sex, and You | <https://hubble-live-assets.s3.eu-west-1.amazonaws.com/easy-health/file_asset/file/303/Love-sex-and-you.pdf> |
|  |  | Let's Talk About Puberty | <https://hubble-live-assets.s3.eu-west-1.amazonaws.com/easy-health/file_asset/file/301/Lets_Talk_About_Puberty.pdf> |
|  |  | How To Put On a Condom | <https://hubble-live-assets.s3.eu-west-1.amazonaws.com/easy-health/file_asset/file/121/How-to-put-on-a-condom.jpg> |
|  |  | The Contraceptive Implant | <https://hubble-live-assets.s3.eu-west-1.amazonaws.com/easy-health/file_asset/file/122/the-contraceptive-implant.pdf> |
|  |  | Visitng the Sexual Health Clinic | <https://hubble-live-assets.s3.eu-west-1.amazonaws.com/easy-health/file_asset/file/4/Visiting-the-sexual-health-clinic.pdf> |
|  |  | Health Action Plan | [Easy Health](https://www.easyhealth.org.uk/resources/category/103-health-action-plan) |
|  |  | Health Action Plans | https://hubble-live-assets.s3.eu-west-1.amazonaws.com/easy-health/file_asset/file/167/health-action-plans.pdf |
|  |  | My Health Action Plan | https://hubble-live-assets.s3.eu-west-1.amazonaws.com/easy-health/file_asset/file/183/My-Health-Action-Plan-Easy-read.pdf |
|  |  | My Health Action Plan | https://hubble-live-assets.s3.eu-west-1.amazonaws.com/easy-health/file_asset/file/176/my-health-action-plan.pdf |
|  |  | My Health Record | https://hubble-live-assets.s3.eu-west-1.amazonaws.com/easy-health/file_asset/file/180/My-health-record.pdf |
|  |  | What is a Health Action Plan? | https://hubble-live-assets.s3.eu-west-1.amazonaws.com/easy-health/file_asset/file/100/What-is-a-health-action-plan.pdf |
|  |  | Get Healthy! Live Longer! | [Get Healthy! Live Longer! \| Easy Health](https://www.easyhealth.org.uk/resources/219-get-healthy-live-longer) |
|  |  | Going for a Prostate Examination | https://hubble-live-assets.s3.eu-west-1.amazonaws.com/easy-health/file_asset/file/1471/Going-for-a-Prostate-Examination__Easy_Read_.pdf |
|  |  | Attend Anywhere Appointments [Information for Patients] | https://flipbooks.leedsth.nhs.uk/LN004801.pdf |
|  |  | Cervical screening myth busting: Cali's story | https://www.youtube.com/watch?v=U8DHFXeKrBk |
|  |  | About ovarian cancer | https://hubble-live-assets.s3.eu-west-1.amazonaws.com/easy-health/file_asset/file/1414/About_Ovarian_Cancer__Ovacome_.pdf |
|  |  | Tests for ovarian cancer | https://hubble-live-assets.s3.eu-west-1.amazonaws.com/easy-health/file_asset/file/1416/Tests_for_Ovarian_Cancer__Ovacome_.pdf |
|  |  | Stages of ovarian cancer | https://hubble-live-assets.s3.eu-west-1.amazonaws.com/easy-health/file_asset/file/1412/Stages_of_Ovarian_Cancer__Ovacome_.pdf |
|  |  | Ovarian cancer signs | https://hubble-live-assets.s3.eu-west-1.amazonaws.com/easy-health/file_asset/file/1410/What_is_Ovarian_Cancer__Ovacome_.pdf |
|  |  | Making a decision about enlarged (big) prostate | https://hubble-live-assets.s3.eu-west-1.amazonaws.com/easy-health/file_asset/file/1389/Making_a_decision_about_an_Enlarged_Prostate-easy-read-version__Winton_Centre_.pdf |
|  |  | Cervical Screening Myths [Questions & Answers] | https://hubble-live-assets.s3.eu-west-1.amazonaws.com/easy-health/file_asset/file/1347/Cervical_Screening_Q_A__2_.mp4 |
|  |  | Easy Read - Fibroids | https://hubble-live-assets.s3.eu-west-1.amazonaws.com/easy-health/file_asset/file/1323/endometriosis.pdf |
|  |  | Easy Read - What is a Learning Disability | https://hubble-live-assets.s3.eu-west-1.amazonaws.com/easy-health/file_asset/file/1285/Easy_read_leaflet_-_What_is_a_learning_disability.pdf |
|  |  | Reasonably Adjusted Respiratory Clinics for Patients with Asthma and a Learning Disability | https://www.youtube.com/watch?v=h9wIhNEZHLc |
|  |  | Diagnosed with primary breast cancer | https://hubble-live-assets.s3.eu-west-1.amazonaws.com/easy-health/file_asset/file/1263/easy_read_-_bcn255_diagnosed_with_primary_breast_cancer.pdf |
|  |  | What is an annual health check: Easy Health accessible video | https://youtu.be/ulOPUo7HJz0 |
|  |  | My Appointment Easy-read card (with pictures) | https://hubble-live-assets.s3.eu-west-1.amazonaws.com/easy-health/file_asset/file/1008/CCG_My_Appointment_Easy-Read__1_.pdf |
|  |  | My appointment card (text only) | https://hubble-live-assets.s3.eu-west-1.amazonaws.com/easy-health/file_asset/file/1009/CCG_My_Appointment_Written__1_.pdf |
|  |  | Your guide to getting a good healthcare if you have a learning disability | https://hubble-live-assets.s3.eu-west-1.amazonaws.com/easy-health/file_asset/file/975/MacMillan_easy_read_-_7_steps_to_equal_health_care-Your_guide_to_getting_good_healthcare.pdf |
|  |  | What is a Learning Disability? | https://hubble-live-assets.s3.eu-west-1.amazonaws.com/easy-health/file_asset/file/886/What-is-LD-leaflet-easy_read.pdf |
|  |  | About having a health check | https://hubble-live-assets.s3.eu-west-1.amazonaws.com/easy-health/file_asset/file/181/About-Having-a-health-check.pdf |
|  |  | Gold Standard Health Check | https://hubble-live-assets.s3.eu-west-1.amazonaws.com/easy-health/file_asset/file/182/Gold-Standard-Health-Check.pdf |
|  |  | Health Is Everyone's Responsibility | <https://youtu.be/p4T9QrUchTU> |
|  |  | What To Expect At Your Annual Health Check | https://hubble-live-assets.s3.eu-west-1.amazonaws.com/easy-health/file_asset/file/184/What-To-Expect-During-Your-Learning-Disability-Health-Check.pdf |
|  |  | Cold Weather Plan for England - Keeping healthy when it is really cold | https://hubble-live-assets.s3.eu-west-1.amazonaws.com/easy-health/file_asset/file/994/Cold_Weather_Plan_for_England_-_Keeping_healthy_when_it_is_cold.pdf |
|  |  | Dealing with damp and mould in your home | https://hubble-live-assets.s3.eu-west-1.amazonaws.com/easy-health/file_asset/file/992/Damp-Mold.pdf |
|  |  | Beat the Heat | https://hubble-live-assets.s3.eu-west-1.amazonaws.com/easy-health/file_asset/file/900/Beat_the_Heat_Poster_2022.pdf |
|  |  | An Easy Read Guide to Self Care | https://www.easyhealth.org.uk/resources/394-an-easy-read-guide-to-self-care |
|  |  | Healthy Lifestyle 1 | https://hubble-live-assets.s3.eu-west-1.amazonaws.com/easy-health/file_asset/file/334/healthy_lifestyle_1.pdf |
|  |  | Healthy Lifestyle 2 | https://hubble-live-assets.s3.eu-west-1.amazonaws.com/easy-health/file_asset/file/335/healthy_lifestyle_2.pdf |
|  |  | How Can You Be Healthy? | https://hubble-live-assets.s3.eu-west-1.amazonaws.com/easy-health/file_asset/file/336/How-you-can-be-healthy.pdf |
|  |  | Making Choices About Your Healthcare | https://hubble-live-assets.s3.eu-west-1.amazonaws.com/easy-health/file_asset/file/105/Making-choices.pdf |
| Eat for Health | [Eat For Health](https://www.eatforhealth.gov.au/) | Eat for Health calculators | [Eat for health calculators \| Eat For Health](https://www.eatforhealth.gov.au/eat-health-calculators) |
|  |  | Sample meal plan for men | [Sample meal plan for men \| Eat For Health](https://www.eatforhealth.gov.au/food-essentials/how-much-do-we-need-each-day/sample-meal-plan-men) |
|  |  | Sample meal plan for men Children aged 9 - 11 years sample meal plan | [adg_sample_meal_plan_child.pdf (eatforhealth.gov.au)](https://www.eatforhealth.gov.au/sites/default/files/2022-08/adg_sample_meal_plan_child.pdf) |
|  |  | Men aged 19 - 50 years sample meal plan | <https://www.eatforhealth.gov.au/sites/default/files/2022-08/adg_sample_meal_plan_men.pdf> |
|  |  | [Women aged 19 - 50 years sample meal plan](https://www.eatforhealth.gov.au/sites/default/files/2022-08/adg_sample_meal_plan_women.pdf) | [Women aged 19 - 50 years sample meal plan](https://www.eatforhealth.gov.au/sites/default/files/2022-08/adg_sample_meal_plan_women.pdf) |
| Family Planning Australia | <https://www.fpnsw.org.au/> | Being a Healthy Woman Factsheets | [Being a Healthy Woman Factsheets \| Family Planning NSW (fpnsw.org.au)](https://www.fpnsw.org.au/beingahealthywomanfactsheets) |
|  |  | Just checking - Cervical screening | https://www.youtube.com/watch?v=Hv98f_UxS_I |
|  |  | Betty's story | <https://www.fpnsw.org.au/sites/default/files/assets/Bettys_Story_20180423.pdf> |
|  |  | Just checking - Breast screening | https://www.youtube.com/watch?v=p4ozp5-dHfQ |
|  |  | Lisa's story | https://www.fpnsw.org.au/sites/default/files/assets/Lisas_Story_20180423.pdf |
|  |  | Just checking - Bowel screening | https://www.youtube.com/watch?v=idCEbIxhqJw |
|  |  | Bob's story | https://www.fpnsw.org.au/sites/default/files/assets/Bobs_Story_20180423.pdf |
|  |  | Just Checking- Easy English | <https://www.fpnsw.org.au/justchecking/easyenglish> |
|  |  | Safe sex and fun | <https://www.fpnsw.org.au/factsheets/individuals/disability/sex-safe-and-fun> |
|  |  | Just Checking- support person's tool | [Support_Persons_Tool_v02.pdf (fpnsw.org.au)](https://www.fpnsw.org.au/sites/default/files/assets/Support_Persons_Tool_v02.pdf) |
| Foundation for People with Learning Disabilities | <https://www.learningdisabilities.org.uk/> | An Easy Read Guide to Anxiety | [An Easy Read Guide to Anxiety \| Foundation for People with Learning Disabilities](https://www.learningdisabilities.org.uk/learning-disabilities/publications/easy-read-guide-anxiety) |
|  |  | Feeling Down: looking after my mental health | [Feeling down: looking after my mental health \| Foundation for People with Learning Disabilities](https://www.learningdisabilities.org.uk/learning-disabilities/publications/feeling-down-looking-after-my-mental-health) |
|  |  | Depression and learning disability | [Depression and learning disability \| Foundation for People with Learning Disabilities](https://www.learningdisabilities.org.uk/learning-disabilities/a-to-z/d/depression-and-learning-disability) |
|  |  | A Carer’s Guide to Depression in People with a Learning Disability | [Microsoft Word - A carers guide to depression[1].doc (moodcafe.co.uk)](https://www.moodcafe.co.uk/media/25008/Acarersguidetodepression.pdf) |
| General Medical Council | [https://www.gmc-uk.org](https://www.gmc-uk.org/) | Going to the doctor: what should happen | <https://www.gmc-uk.org/-/media/documents/going-to-the-doctor-what-should-happen---easy-read--english-1114_pdf-53919415.pdf> |
|  |  | What to do if you are not happy with your doctor | <https://www.gmc-uk.org/-/media/gmc-site/concerns/3274-gmc-concerns-about-a-doctor-easy-read-v2.pdf> |
| Great Ormond Street Hospital for Children | <https://www.gosh.nhs.uk/> | Easy Read information sheets | [Easy Read information sheets \| Great Ormond Street Hospital (gosh.nhs.uk)](https://www.gosh.nhs.uk/your-hospital-visit/easy-read-information-sheets/) |
|  |  | After you have had your tooth out (Easy Read) | https://media.gosh.nhs.uk/documents/After_you_have_had_a_tooth_out_ER0095_FINAL_Jun20_0.pdf |
|  |  | ECMO | https://media.gosh.nhs.uk/documents/ECMO_ER0064_FINAL_Sep16.pdf |
|  |  | Having a blood test (Easy Read) | https://media.gosh.nhs.uk/documents/Having_a_blood_test_ER0011_FINAL_May20.pdf |
|  |  | Having a clinic appointment | https://media.gosh.nhs.uk/documents/Having_a_clinic_appointment_ER0007_FINAL_Sep18_0.pdf |
|  |  | Having a drip (Easy Read) | https://media.gosh.nhs.uk/documents/Having_a_drip_ER0306_FINAL_Apr20.pdf |
|  |  | Having an NG tube | https://media.gosh.nhs.uk/documents/Having_an_NG_tube_ER0034_FINAL_Sep18.pdf |
|  |  | Injections for scars | https://media.gosh.nhs.uk/documents/Having_scar_injections_ER0236_FINAL_Oct18.pdf |
|  |  | Helping you cope with procedures | https://media.gosh.nhs.uk/documents/Helping_you_cope_with_procedures_ER0365_FINAL_Jul20.pdf |
|  |  | How to tell us a concern or make a complaint (Easy Read) | https://media.gosh.nhs.uk/documents/Helping_you_cope_with_procedures_ER0365_FINAL_Jul20.pdf |
|  |  | Keeping your bowels healthy | https://media.gosh.nhs.uk/documents/Healthy_bowels_ER0062_FINAL_Mar17_0.pdf |
|  |  | Growing up, gaining independence: families | <https://media.gosh.nhs.uk/documents/Growing_up_gaining_independence_ER0191_FINAL_Aug18.pdf> |
|  |  | Growing up, Gaining Independence: information for families of young people with a Learning Disability | https://media.gosh.nhs.uk/documents/GUGI_for_young_people_with_a_learning_diability.pdf |
|  |  | Keeping things private | https://media.gosh.nhs.uk/documents/Confidentiality_ER0223_FINAL_Sep18.pdf |
|  |  | Giving consent | https://media.gosh.nhs.uk/documents/Giving_consent_ER0024_FINAL_Sep18.pdf |
|  |  | Keeping organised and safe | https://media.gosh.nhs.uk/documents/Keeping_organised_and_safe_ER0227_FINAL_Sep18.pdf |
|  |  | Legal and financial stuff | https://media.gosh.nhs.uk/documents/Legal_and_financial_stuff_F0170_FINAL_Aug18.pdf |
|  |  | Managing your appointments | https://media.gosh.nhs.uk/documents/Managing_your_appointments_ER0229_FINAL_Sep18.pdf |
|  |  | Seeing the doctor on your own | https://media.gosh.nhs.uk/documents/Seeing_the_doctor_on_your_own_ER0169_FINAL_Aug18.pdf |
|  |  | Understanding your health | https://media.gosh.nhs.uk/documents/Understanding_your_health_ER0171_FINAL_Aug18.pdf |
|  |  | Having a 3 minute step test (Easy Read) | https://media.gosh.nhs.uk/documents/Having_a_3_minute_step_test_ER0333_FINAL_Apr20.pdf |
|  |  | Having a 6 minute walk test (Easy Read) | https://media.gosh.nhs.uk/documents/Having_a_6_Minute_Walk_test_ER0334_FINAL_Apr20.pdf |
|  |  | Having a blood clot removed (Easy Read) | https://media.gosh.nhs.uk/documents/Having_a_blood_clot_removed_ER0090_FINAL_Apr20.pdf |
|  |  | Having a bone scan | https://media.gosh.nhs.uk/documents/Having_a_bone_scan_ER0239_FINAL_Mar19.pdf |
|  |  | Having a brain angiogram (Easy Read) | https://media.gosh.nhs.uk/documents/Having_a_brain_angiogram_ER0104_FINAL_Apr20.pdf |
|  |  | Having a bronchoscopy and bronchogram (Easy Read) | https://media.gosh.nhs.uk/documents/Having_a_bronchoscopy_and_bronchogram_ER0088_FINAL_Apr20.pdf |
|  |  | Having colonic manometry (Easy Read) | https://media.gosh.nhs.uk/documents/Having_colonic_manometry_ER0247_FINAL_Apr20.pdf |
|  |  | Having a check-up before you fly (Easy Read) | https://media.gosh.nhs.uk/documents/Having_a_check_up_before_you_fly_ER0115_FINAL_Mar20.pdf |
|  |  | Having a contrast enema | https://media.gosh.nhs.uk/documents/Having_a_contrast_enema_ER0070_FINAL_Aug18.pdf |
|  |  | Having a contrast scan | https://media.gosh.nhs.uk/documents/Having_a_contrast_scan_ER0022_FINAL_Aug18.pdf |
|  |  | Having a CT scan | <https://media.gosh.nhs.uk/documents/Having_a_CT_scan_ER0021_FINAL_Mar19.pdf> |
|  |  | Having a CT scan under general anaesthetic | https://media.gosh.nhs.uk/documents/Having_a_CT_scan_with_GA_ER0218_FINAL_Mar19.pdf |
|  |  | Having a CT scan with sedation | https://media.gosh.nhs.uk/documents/Having_a_CT_scan_with_sedation_ER0222_FINAL_Mar19.pdf |
|  |  | Having a DMSA scan | https://media.gosh.nhs.uk/documents/Having_a_DMSA_scan_ER0112_FINAL_Mar19.pdf |
|  |  | Having a foodpipe manometry test (Easy Read) | https://media.gosh.nhs.uk/documents/Having_a_foodpipe_manometry_test_ER0248_FINAL_May20.pdf |
|  |  | Having a lung scan | https://media.gosh.nhs.uk/documents/Having_a_lung_scan_ER0134_FINAL_Mar19.pdf |
|  |  | Having a MAG3 scan | https://media.gosh.nhs.uk/documents/Having_a_MAG3_scan_ER0189_FINAL_Mar19.pdf |
|  |  | Having a MCUG bladder scan (Easy Read) | https://media.gosh.nhs.uk/documents/Having_a_MCUG_bladder_scan_ER0073_FINAL_Jun20.pdf |
|  |  | Having a nasal nitric oxide test (Easy Read) | https://media.gosh.nhs.uk/documents/Having_a_nasal_nitric_oxide_test_ER0335_FINAL_Apr20.pdf |
|  |  | Having a nephrostogram scan | https://media.gosh.nhs.uk/documents/Having_a_nephrostogram_ER0140_FINAL_Apr18.pdf |
|  |  | Having a nerve block (Easy Read) | <https://media.gosh.nhs.uk/documents/Having_a_nerve_block_ER0187_FINAL_Apr20.pdf> |
|  |  | Having a pregancy test before treatment | https://media.gosh.nhs.uk/documents/Having_a_pregnancy_test_ER0040_FINAL_Jul15.pdf |
|  |  | Having a salivary gland scan | https://media.gosh.nhs.uk/documents/Having_a_salivary_gland_scan_ER0151_FINAL_Apr18_0.pdf |
|  |  | Having a skin prick allergy test (Easy Read) | https://media.gosh.nhs.uk/documents/Having_a_skin_prick_allergy_test_ER0324_FINAL_Nov20.pdf |
|  |  | Having a transit study (Easy Read) | https://media.gosh.nhs.uk/documents/Having_a_transit_study_ER0158_FINAL_Jul20.pdf |
|  |  | Having a tummy assessment | https://media.gosh.nhs.uk/documents/Having_a_tummy_assessment_ER0220_FINAL_Feb19.pdf |
|  |  | Having a venogram | https://media.gosh.nhs.uk/documents/Having_a_venogram_ER0159_FINAL_Jul18.pdf |
|  |  | Having an anorectal manometry test (Easy Read) | https://media.gosh.nhs.uk/documents/Having_an_anorectal_manometry_test_ER0113_FINAL_May20.pdf |
|  |  | Having an Echo | https://media.gosh.nhs.uk/documents/Having_an_Echo.pdf |
|  |  | Having an ECG | https://media.gosh.nhs.uk/documents/Having_an_ECG_ER0019_FINAL_Nov14.pdf |
|  |  | Having an EGG (Easy Read) | https://media.gosh.nhs.uk/documents/Having_an_EGG_ER0253_FINAL_Jul20.pdf |
|  |  | Having an EEG (Easy Read) | https://media.gosh.nhs.uk/documents/Having_an_EEG_FINAL_Jul20.pdf |
|  |  | Having an Evoked Potential test (Easy Read) | https://media.gosh.nhs.uk/documents/Having_an_Evoked_Potential_test_FINAL_Jul20.pdf |
|  |  | Having an Exercise Induced Asthma test (Easy Read) | https://media.gosh.nhs.uk/documents/Having_an_Exercise_Induced_Asthma_test_ER0332_FINAL_Nov20_0.pdf |
|  |  | Having an eye angiogram (Easy Read) | https://media.gosh.nhs.uk/documents/Having_an_eye_angiogram_ER0323_FINAL_Jun20.pdf |
|  |  | Having an ultrasound scan | https://media.gosh.nhs.uk/documents/Having_an_ultrasound_scan_ER0015_FINAL_Aug18.pdf |
|  |  | Having an x-ray | <https://media.gosh.nhs.uk/documents/Having_an_x-ray_ER0008_FINAL_Aug18.pdf> |
|  |  | Having antroduodenal manometry (Easy Read) | https://media.gosh.nhs.uk/documents/Having_antroduodenal_manometry_ER0116_FINAL_Jul20.pdf |
|  |  | Having BAEP (Easy Read) | https://media.gosh.nhs.uk/documents/Having_BAEP_FINAL_Jul20.pdf |
|  |  | Having home video telemetry HVT (Easy Read) | https://media.gosh.nhs.uk/documents/Having_home_video_telemetry_ER0392_FINAL_Jul20.pdf |
|  |  | Having salivary gland injections | https://media.gosh.nhs.uk/documents/12ER0029_Having_salivary_gland_injections.pdf |
|  |  | Having SSEP (Easy Read) | https://media.gosh.nhs.uk/documents/Having_SSEP_FINAL_Jul20.pdf |
|  |  | Having a sleep EEG (Easy Read) | https://media.gosh.nhs.uk/documents/Having_a_sleep_EEG_ER0294_FINAL_Jul20.pdf |
|  |  | Having a sleep study (Easy Read) | https://media.gosh.nhs.uk/documents/Having_a_sleep_study_ER0152_FINAL_Apr20.pdf |
|  |  | Having spinal cord monitoring during an operation (Easy Read) | https://media.gosh.nhs.uk/documents/Having_spinal_cord_monitoring_during_an_operation_ER0393_FINAL_Jul20.pdf |
|  |  | Having telemetry (Easy Read) | https://media.gosh.nhs.uk/documents/Having_telemetry_monitoring_ER0242_FINAL_Jul20.pdf |
|  |  | Having VEP (Easy Read) | https://media.gosh.nhs.uk/documents/Having_VEP_FINAL_Jul20.pdf |
|  |  | What happens in a breathing test | https://media.gosh.nhs.uk/documents/Having_breathing_tests_ER0125_FINAL_Apr18.pdf |
|  |  | Having a biopsy (Easy Read) | https://media.gosh.nhs.uk/documents/Having_a_biopsy_ER0031_FINAL_Apr20.pdf |
|  |  | Having a cochlear implant operation (Easy Read) | https://media.gosh.nhs.uk/documents/Having_a_cochlear_implant_operation_ER0280_FINAL_Jul20.pdf |
|  |  | Having a drain (Easy Read) | https://media.gosh.nhs.uk/documents/Having_a_drain_ER0030_FINAL_Apr20.pdf |
|  |  | Having a heart operation | https://media.gosh.nhs.uk/documents/Having_a_heart_operation_ER0065_FINAL_Sep16.pdf |
|  |  | Having a liver biopsy (Easy Read) | https://media.gosh.nhs.uk/documents/Having_a_liver_biopsy_ER0133_FINAL_Apr20.pdf |
|  |  | Having a lumar puncture (Easy Read) | https://media.gosh.nhs.uk/documents/Having_a_lumbar_puncture_ER0245_FINAL_Apr20.pdf |
|  |  | Having a muscle biopsy (Easy Read) | https://media.gosh.nhs.uk/documents/Having_a_muscle_biopsy_ER0141_FINAL_Apr20.pdf |
|  |  | Having assessment before craniofacial surgery | https://media.gosh.nhs.uk/documents/Having_assessment_before_craniofacial_surgery_ER0249_v2_Apr20.pdf |
|  |  | Having dental treatment under sedation | https://media.gosh.nhs.uk/documents/Having_dental_treatment_under_sedation_ER0126_v1_Jun17.pdf |
|  |  | Having embolisation (Easy Read) | https://media.gosh.nhs.uk/documents/Having_embolisation_ER0083_FINAL_Apr20.pdf |
|  |  | Having oesophageal dilation | https://media.gosh.nhs.uk/documents/Having_oesophageal_dilatation_ER0084_FINAL_Apr18.pdf |
|  |  | Having salivary gland sclerotherapy | https://media.gosh.nhs.uk/documents/Having_sclerotherapy_ER0085_FINAL_Apr20.pdf |
|  |  | Having a skin biopsy (Easy Read) | https://media.gosh.nhs.uk/documents/Having_a_skin_biopsy_ER0147_FINAL_Apr20.pdf |
|  |  | Having craniofacial surgery without a frame (Easy Read) | https://media.gosh.nhs.uk/documents/Having_craniofacial_surgery_no_frame_ER0252_FINAL_Apr20.pdf |
|  |  | Having craniofacial surgery with a frame (Easy Read) | https://media.gosh.nhs.uk/documents/Having_craniofacial_surgery_with_frame_ER0250_FINAL_Apr20.pdf |
|  |  | Having craniofacial surgery with springs (Easy Read) | https://media.gosh.nhs.uk/documents/Having_craniofacial_surgery_with_springs_ER0309_v1_Apr20.pdf |
|  |  | Having your nose re-shaped (Easy Read) | https://media.gosh.nhs.uk/documents/Having_your_nose_reshaped_ER0251_FINAL_Apr20.pdf |
|  |  | Having your veins lasered (Easy Read) | https://media.gosh.nhs.uk/documents/Having_your_veins_lasered_ER0089_FINAL_Apr20.pdf |
|  |  | How to take liquid medicine (Easy Read) | https://media.gosh.nhs.uk/documents/How_to_take_liquid_medicine_ER0048_Apr20.pdf |
|  |  | How to take tablets or capsules (Easy Read) | https://media.gosh.nhs.uk/documents/How_to_take_tablets_or_capsules_ER0049_Apr20.pdf |
|  |  | How to use ear drops (Easy Read) | https://media.gosh.nhs.uk/documents/How_to_use_ear_drops_ER0050_Apr20.pdf |
|  |  | How to use eye drops (Easy Read) | https://media.gosh.nhs.uk/documents/How_to_use_eye_drops_ER0047_Apr20.pdf |
|  |  | How to use nose drops (Easy Read) | https://media.gosh.nhs.uk/documents/How_to_use_nose_drops_ER0046_Apr20.pdf |
|  |  | How to use an inhaler (Easy Read) | https://media.gosh.nhs.uk/documents/How_to_use_an_inhaler_ER0330_Apr20.pdf |
|  |  | How to use a suppository (Easy Read) | https://media.gosh.nhs.uk/documents/How_to_use_a_suppository_ER0331_Apr20.pdf |
|  |  | How to use eye ointment (Easy Read) | https://media.gosh.nhs.uk/documents/How_to_use_eye_ointment_ER0329_Apr20.pdf |
| Health Care Access Research and Developmental Disabilities (H-CARDD) | [Family Matters Toolkit (ddtoolkits.com)](https://familymatters.ddtoolkits.com/) | Resources for Caregivers | [Resources for Caregivers \| CAMH](https://www.camh.ca/en/professionals/professionals--projects/hcardd/health-care-resources/resources-for-caregivers) |
| Healthy Transitions | [Healthy Transitions \| Moving from Pediatric to Adult Health Care (healthytransitionsny.org)](https://healthytransitionsny.org/) | Guides | [Guides \| Healthy Transitions (healthytransitionsny.org)](https://healthytransitionsny.org/skills/) |
|  |  | Videos | [Videos \| Healthy Transitions (healthytransitionsny.org)](https://healthytransitionsny.org/videos/) |
| Heart Foundation | <https://www.heartfoundation.org.au/> | Healthy Living & Eating | [Healthy Living \| Heart Foundation](https://www.heartfoundation.org.au/Bundles/Healthy-Living-and-Eating?selectedfilter=heart%20health%20basics) |
|  |  | Physical activity and your heart health | <https://www.heartfoundation.org.au/healthy-living/physical-activity/physical-activity-and-exercise> |
|  |  | Personal Walking Plans | <https://heartfoundation.typeform.com/to/kwgdZH9V/?_gl=1*1gqukmv*_gcl_au*MTMwNjQ5NzgwOS4xNzI2ODE1MDA4*_ga*MTkwMjA3NTgyNC4xNzI2ODE1MDA5*_ga_1PZSTHJ79S*MTcyNjgxNTAwOS4xLjEuMTcyNjgxNTI4MS4zMC4wLjA.*_fplc*M2phOHJmRmdHR3ZMdE03RzlLUno4NzdPREJGRGR4cFVhTlBZdmxCUmF5STA3cnRsTCUyRmZWbGJGT0dDcTR2aTl5TlRLN3UwOVhwT1kzTk9NUzNsVWlhalc3QjltTCUyQk10MnNVVyUyQm9GaTNIZURVc1NUc3RMRmRmRHRMaElMT05BJTNEJTNE&typeform-source=walkingplans.heartfoundation.org.au> |
|  |  | Keep your heart healthy | [Keeping your heart healthy \| Heart Foundation](https://www.heartfoundation.org.au/healthy-living/keeping-your-heart-healthy) |
|  |  | Heart Foundation Walking | <https://www.heartfoundation.org.au/healthy-living/heart-foundation-walking> |
|  |  | Time to book a Heart Health Check? | [Time to book a Heart Health Check? \| Heart Foundation](https://www.heartfoundation.org.au/your-heart/heart-health-checks) |
|  |  | Benefits of physical activity for your heart | <https://www.heartfoundation.org.au/healthy-living/physical-activity/benefits-of-physical-activity> |
|  |  | Physical activity after a heart attack | <https://www.heartfoundation.org.au/your-heart/support/physical-activity-after-a-heart-attack> |
| Ideas | <http://www.ideas.org.au/> | Be Kind to your Kidneys | [Be Kind to Your Kidneys (ideas.org.au)](https://www.ideas.org.au/blogs/kind-to-kidneys.html?highlight=WyJoZWFsdGgiLCJoZWFsdGgncyJd) |
|  |  | How to get a Mental Health Care Plan | [How to get a Mental Health Care Plan (ideas.org.au)](https://www.ideas.org.au/blogs/how-to-get-a-mental-health-plan.html?highlight=WyJoZWFsdGgiLCJoZWFsdGgncyJd) |
|  |  | Telehealth | [Telehealth (ideas.org.au)](https://www.ideas.org.au/blogs/telehealth.html?highlight=WyJoZWFsdGgiLCJoZWFsdGgncyJd) |
|  |  | Women's Health Checks | [Women's Health Checks (ideas.org.au)](https://www.ideas.org.au/blogs/women-s-health-checks.html?highlight=WyJoZWFsdGgiLCJoZWFsdGgncyJd) |
|  |  | Annual Health Checks | [Annual Health Checks (ideas.org.au)](https://www.ideas.org.au/blogs/annual-health-checks.html?highlight=WyJoZWFsdGgiLCJoZWFsdGgncyJd) |
|  |  | Men's Health Checks | [Men's Health Checks (ideas.org.au)](https://www.ideas.org.au/blogs/men-s-health-checks.html) |
| Inclusion Australia | <https://www.inclusionaustralia.org.au/> | Living with Covid | [Living with COVID – Inclusion Australia](https://www.inclusionaustralia.org.au/resource/living-with-covid/) |
| Intellectual Disability Mental Health Connect | <https://idmhconnect.health/> | Information for people with intellectual disability | [I am a person with intellectual disability \| Intellectual Disability Mental Health Connect (idmhconnect.health)](https://idmhconnect.health/i-am-person-ID) |
|  |  | Tips for good mental health | <https://idmhconnect.health/tips-good-mental-health/ER> |
|  |  | Services for mental health | <https://idmhconnect.health/i-am-person-ID/services-mental-health> |
|  |  | Changes in my mental health | <https://idmhconnect.health/i-am-person-ID/changes-my-mental-health> |
|  |  | Getting support for my mental health | https://idmhconnect.health/i-am-person-ID/getting-support-my-mental-health |
|  |  | Communicating about my mental health | <https://idmhconnect.health/communicating-about-my-mental-health/ER> |
|  |  | First Nations peoples | [First Nations peoples \| Intellectual Disability Mental Health Connect (idmhconnect.health)](https://idmhconnect.health/first-nations-peoples/ER) |
|  |  | Culturally and linguistically diverse people | [Culturally and linguistically diverse people \| Intellectual Disability Mental Health Connect (idmhconnect.health)](https://idmhconnect.health/culturally-and-linguistically/ER) |
|  |  | LGBTQ+ people | [LGBTQ+ people \| Intellectual Disability Mental Health Connect (idmhconnect.health)](https://idmhconnect.health/lgbtq-people/ER) |
|  |  | People living in rural and remote areas | [People living in rural and remote areas \| Intellectual Disability Mental Health Connect (idmhconnect.health)](https://idmhconnect.health/people-living-rural-and-remote-areas/ER) |
|  |  | People in contact with the justice system | [People in contact with the justice system \| Intellectual Disability Mental Health Connect (idmhconnect.health)](https://idmhconnect.health/people-contact-justice-system/ER) |
|  |  | People with many supports | <https://idmhconnect.health/people-multiple-needs/ER> |
|  |  | People who have had trauma | <https://idmhconnect.health/people-who-have-had-trauma/ER> |
|  |  | I am not feeling better | [I am not feeling better \| Intellectual Disability Mental Health Connect (idmhconnect.health)](https://idmhconnect.health/i-am-not-feeling-better/ER) |
|  |  | Recognising mental health problems | [Recognising mental health problems \| Intellectual Disability Mental Health Connect (idmhconnect.health)](https://idmhconnect.health/i-am-family-or-support-person/recognising-mental-health-problems) |
|  |  | Communicating with someone about their mental health | [Communicating with someone about their mental health \| Intellectual Disability Mental Health Connect (idmhconnect.health)](https://idmhconnect.health/i-am-family-or-support-person/communicating-someone-about-their-mental-health) |
|  |  | Supporting someone along a mental health pathway | [Supporting someone along a mental health pathway \| Intellectual Disability Mental Health Connect (idmhconnect.health)](https://idmhconnect.health/i-am-family-or-support-person/supporting-persons-mental-health/supporting-someone-along-pathway) |
|  |  | Working together with the person and their team | [Working together with the person and their team \| Intellectual Disability Mental Health Connect (idmhconnect.health)](https://idmhconnect.health/i-am-family-or-support-person/supporting-persons-mental-health/working-together-person-and-their-team) |
|  |  | Srategies for common challenges | <https://idmhconnect.health/i-am-family-or-support-person/strategies-common-challenges> |
|  |  | Looking after myself | [Looking after myself \| Intellectual Disability Mental Health Connect (idmhconnect.health)](https://idmhconnect.health/i-am-family-or-support-person/looking-after-myself) |
| International Prader-Willi Syndrome Organisation | <https://ipwso.org/> | Diet and nutrition | [Diet and Nutrition - IPWSO](https://ipwso.org/information-for-families/dietary-management/) |
|  |  | Physical health and exercise | [Physical Health and Exercise - IPWSO](https://ipwso.org/information-for-families/physical-health-and-exercise/) |
|  |  | Behaviour and Mental Health | <https://ipwso.org/information-for-families/behaviour-and-mental-health/> |
|  |  | Behaviour and Mental health | [Behaviour and Mental Health - IPWSO](https://ipwso.org/information-for-families/behaviour-and-mental-health/) |
|  |  | Promoting Positive Behaviour | [Promoting Positive Behaviour - IPWSO](https://ipwso.org/information-for-families/behaviour-and-mental-health/promoting-positive-beahaviour/) |
|  |  | Confabulation | [Confabulation - IPWSO](https://ipwso.org/information-for-families/behaviour-and-mental-health/confabulation/) |
|  |  | Managing a Meltdown | [Managing a Meltdown - IPWSO](https://ipwso.org/information-for-families/behaviour-and-mental-health/managing-a-meltdown/) |
|  |  | A guide to rituals and obsessions | [A guide to rituals and obsessions - IPWSO](https://ipwso.org/information-for-families/behaviour-and-mental-health/a-guide-to-rituals-and-obsessions/) |
|  |  | Sexual Development | [Sexual Development - IPWSO](https://ipwso.org/information-for-families/sexual-development/) |
|  |  | Diet and Nutrition | <https://ipwso.org/information-for-families/dietary-management/> |
|  |  | Behaviour and Mental Health | <https://ipwso.org/information-for-families/behaviour-and-mental-health/> |
|  |  | Physical Health and Exercise | <https://ipwso.org/information-for-families/physical-health-and-exercise/> |
| Mencap | [https://www.mencap.org.uk/](https://qcidd.centre.uq.edu.au/) | Help with your mental health and wellbeing | [Help with your mental health and wellbeing \| Mencap](https://www.mencap.org.uk/get-involved/campaign-mencap/listen-us/help-your-mental-health-and-wellbeing) |
|  |  | What is mental health? | [What is mental health? \| Mencap](https://www.mencap.org.uk/easyread/what-mental-health) |
|  |  | How to tell if someone is struggling with their mental health | [How to tell if someone is struggling with their mental health.pdf](file:///C:/Users/Tnevill/Downloads/How%20to%20tell%20if%20someone%20is%20struggling%20with%20their%20mental%20health.pdf) |
|  |  | How to be a good listener | [How to be a good listener \| Mencap](https://www.mencap.org.uk/easyread/how-be-good-listener) |
|  |  | The importance of self-care | <https://www.mencap.org.uk/importance-self-care> |
|  |  | Why annual health checks are so important for people with a learning disability | [Why annual health checks are so important for people with a learning disability \| Mencap](https://www.mencap.org.uk/advice-and-support/health/why-annual-health-checks-are-so-important-people-learning-disability) |
|  |  | What is a Summary Care Record? | <https://www.mencap.org.uk/advice-and-support/health/what-summary-care-record> |
|  |  | Visiting hospital | [Visiting hospital \| Mencap](https://www.mencap.org.uk/get-involved/campaign-mencap/visiting-hospital) |
|  |  | Help in hospital - Hospital passports | [Health Guides: Hospital Passports, Summary Care Records And Flu Jabs \| Mencap](https://www.mencap.org.uk/advice-and-support/health-coronavirus/health-guides) |
|  |  | Reasonable adjustments | <https://www.mencap.org.uk/easyread/about-reasonable-adjustments> |
|  |  | Cancer Screening | [Easy Read Information About Cancer and Cancer Screening \| Mencap](https://www.mencap.org.uk/advice-and-support/health/cancer-screening) |
|  |  | Getting a flu jab | [Flu Jabs For People WIth A Learning Disability \| Mencap](https://www.mencap.org.uk/advice-and-support/health/getting-flu-jab) |
|  |  | Protect yourself from flu, have the flu vaccine | <https://www.mencap.org.uk/sites/default/files/2020-10/PHE_Flu_Easy_Read_Winter_2020_leaflet_A4_.pdf> |
|  |  | Guide to having your flu vaccination (jab) during the coronavirus pandemic | <https://www.mencap.org.uk/sites/default/files/2021-11/PHE_Flu_Vaccination_during_a_pandemic_Easy-Read_A4_flyer_2020_03__WEB_.pdf> |
|  |  | Get your free flu jab | <https://www.mencap.org.uk/sites/default/files/2020-11/PHE_flu_easy_read_poster_2020_2021.pdf> |
|  |  | We are here to help you stay well this winter | <https://www.mencap.org.uk/sites/default/files/2021-12/211220_SWTW2021_EasyRead_Leaflet_WebAcc.pdf> |
|  |  | Vaccine reasonable adjustments | <https://www.mencap.org.uk/easyread/vaccine-reasonable-adjustments> |
|  |  | Coronavirus (COVID-19) | [Easy Read Accessible Information About Coronavirus (COVID-19) For People With A Learning Disability, Families, Carers and Support Workers\| Mencap](https://www.mencap.org.uk/advice-and-support/coronavirus-covid-19) |
|  |  | What to do if you have coronavirus | <https://www.mencap.org.uk/easyread/what-should-i-do-if-i-have-coronavirus> |
|  |  | Tests for coronavirus | <https://www.mencap.org.uk/easyread/coronavirus-tests> |
|  |  | Face coverings | <https://www.mencap.org.uk/easyread/face-coverings> |
|  |  | How to keep clean | <https://www.mencap.org.uk/easyread/coronavirus-keeping-clean> |
|  |  | How to wash your hands | <https://www.mencap.org.uk/easyread/coronavirus-how-wash-your-hands> |
|  |  | Having a vaccine | <https://www.mencap.org.uk/easyread/vaccine-reasonable-adjustments> |
|  |  | The coronavirus booster | <https://www.mencap.org.uk/easyread/coronavirus-booster-vaccine> |
|  |  | Reasonable adjustments when having the vaccine | <https://www.mencap.org.uk/easyread/coronavirus-asking-reasonable-adjustments-when-having-your-vaccine> |
|  |  | Keeping safe in care homes or supported living | <https://www.mencap.org.uk/easyread/keeping-safe-care-homes> |
|  |  | Having visitors in a care home or supported living | <https://www.mencap.org.uk/easyread/visits-care-homes> |
|  |  | What if I have coronavirus and live in a care home? | <https://www.mencap.org.uk/easyread/what-should-i-do-if-i-have-coronavirus-and-live-care-home> |
|  |  | What may happen if I have to go into hospital with coronavirus? | <https://www.mencap.org.uk/easyread/what-may-happen-if-i-have-go-hospital-coronavirus> |
|  |  | Visiting someone in hospital with coronavirus | <https://www.mencap.org.uk/easyread/what-expect-if-someone-i-know-goes-hospital-coronavirus> |
|  |  | Health | [Health and Learning Disability - Advice and Support \| Mencap](https://www.mencap.org.uk/advice-and-support/health) |
|  |  | Getting help with your health | https://www.mencap.org.uk/advice-and-support/health-coronavirus/health-guides |
|  |  | Annual Health Checks | https://www.mencap.org.uk/easyread/annual-health-checks |
|  |  | Vaccines | https://www.mencap.org.uk/easyread/vaccines |
| National Autistic Society | <https://www.autism.org.uk/> | Breastfeeding | <https://www.autism.org.uk/advice-and-guidance/topics/physical-health/breastfeeding> |
|  |  | Physical health | [Physical health (autism.org.uk)](https://www.autism.org.uk/advice-and-guidance/topics/physical-health) |
|  |  | Going to the dentist – a guide for autistic adults | <https://www.autism.org.uk/advice-and-guidance/topics/physical-health/going-to-the-dentist/autistic-adults> |
|  |  | Going to the dentist – a guide for parents and families | <https://www.autism.org.uk/advice-and-guidance/topics/physical-health/going-to-the-dentist/parents> |
|  |  | Menstruation | <https://www.autism.org.uk/advice-and-guidance/topics/physical-health/menstruation> |
|  |  | Autism and menapause | [Menopause (autism.org.uk)](https://www.autism.org.uk/advice-and-guidance/topics/physical-health/menopause) |
|  |  | My health passport | <https://dy55nndrxke1w.cloudfront.net/file/24/.ZuLn47.Z3Oq7eJ.ZkJp.0QsoHD/Health_Passport_A4_Editable_2022.pdf> |
|  |  | Guidance notes for My Health  Passport | [Health_Passport_Guide_A4_2020.pdf (dy55nndrxke1w.cloudfront.net)](https://dy55nndrxke1w.cloudfront.net/file/24/WFAYQXOWFvh3OwiWFD2qWF1hkRN/Health_Passport_Guide_A4_2022.pdf) |
|  |  | Pregnancy and childbirth | <https://www.autism.org.uk/advice-and-guidance/topics/physical-health/pregnancy-and-childbirth> |
|  |  | Sleep – a guide for autistic adults | <https://www.autism.org.uk/advice-and-guidance/topics/physical-health/sleep/autistic-adults> |
|  |  | Sleep - a guide for parents of autistic children | <https://www.autism.org.uk/advice-and-guidance/topics/physical-health/sleep/parents> |
|  |  | Mental health | [Mental health (autism.org.uk)](https://www.autism.org.uk/advice-and-guidance/topics/mental-health) |
|  |  | Inpatient mental health hospitals | [Autistic people and inpatient mental health hospitals (autism.org.uk)](https://www.autism.org.uk/advice-and-guidance/topics/inpatient-mental-health-hospitals/autistic-people-and-inpatient-mental-health-hospit) |
|  |  | Loneliness | [Loneliness (autism.org.uk)](https://www.autism.org.uk/advice-and-guidance/topics/loneliness) |
|  |  | Sex education - a guide for parents | <https://www.autism.org.uk/advice-and-guidance/topics/family-life-and-relationships/sex-education/parents-and-carers> |
| National Disability Services | <https://www.nds.org.au/> | Tasmanian Healthy Eating Project: Cooking Videos | [Tasmanian Healthy Eating Project: Cooking videos (nds.org.au)](https://www.nds.org.au/index.php/resources/all-resources/tasmanian-healthy-eating-project-cooking-videos) |
| NDIS Quality and Safeguards Commission | <https://www.ndiscommission.gov.au/> | Practice Alert – Sun and summer safety | <https://www.ndiscommission.gov.au/sites/default/files/2022-12/Practice%20Alert%20%20Sun%20and%20summer%20safety%20accessible.docx> |
| NHS | <https://www.nhs.uk/> | Managing weight with a learning disability | [Managing weight with a learning disability - NHS (www.nhs.uk)](https://www.nhs.uk/live-well/healthy-weight/managing-your-weight/managing-weight-with-a-learning-disability/) |
|  |  | Going to hopsital - Easy Read | [Going to hospital - Easy Read : University College London Hospitals NHS Foundation Trust (uclh.nhs.uk)](https://www.uclh.nhs.uk/patients-and-visitors/patient-information-pages/going-hospital-easy-read) |
|  |  | Having an x-ray | https://flipbooks.leedsth.nhs.uk/LN005493.pdf |
|  |  | STOMP: Stopping the over medication of people with a learning disability, autism or both | https://www.england.nhs.uk/wp-content/uploads/2018/02/stomp-easy-read-leaflet.pdf |
|  |  | Annual Health Checks | [Learning disabilities - Annual health checks - NHS (www.nhs.uk)](https://www.nhs.uk/conditions/learning-disabilities/annual-health-checks/) |
| Organisation for Autism Research | [https://researchautism.org](https://ipwso.org/) | Sex Ed for Self-Advocates | <https://researchautism.org/self-advocates/sex-ed-for-self-advocates/> |
|  |  | Public Versus Private | <https://researchautism.org/self-advocates/sex-ed-for-self-advocates/public-versus-private/> |
|  |  | Puberty and the Body | <https://researchautism.org/self-advocates/sex-ed-for-self-advocates/puberty/> |
|  |  | Healthy Relationships | <https://researchautism.org/self-advocates/sex-ed-for-self-advocates/healthy-relationships/> |
|  |  | All About Consent | <https://researchautism.org/self-advocates/sex-ed-for-self-advocates/consent/> |
|  |  | Dating 101 | <https://researchautism.org/self-advocates/sex-ed-for-self-advocates/dating-101/> |
|  |  | Sexual Orientation and Gender Identity | <https://researchautism.org/self-advocates/sex-ed-for-self-advocates/sexual-orientation-and-gender-identity/> |
|  |  | Am I Ready? | <https://researchautism.org/self-advocates/sex-ed-for-self-advocates/am-i-ready/> |
|  |  | Sexual Activity | <https://researchautism.org/self-advocates/sex-ed-for-self-advocates/sexual-activity/> |
|  |  | Online Relationships and Safety | <https://researchautism.org/self-advocates/sex-ed-for-self-advocates/online-relationships-and-safety/> |
| Planet Puberty | <https://www.planetpuberty.org.au/> | The Body | [The Body - Planet Puberty](https://www.planetpuberty.org.au/the-body/) |
|  |  | Breasts and Bras | <https://www.planetpuberty.org.au/wp-content/uploads/2021/10/pp-breasts-and-bras.pdf> |
|  |  | Feeling Good | [Feeling good - Planet Puberty](https://www.planetpuberty.org.au/feeling-good/) |
|  |  | Cleaning your penis | <https://www.planetpuberty.org.au/wp-content/uploads/2021/02/pp-cleaning-your-penis-1.pdf> |
|  |  | Introduction to Contraception | <https://www.planetpuberty.org.au/wp-content/uploads/2021/02/pp-introduction-to-contraception.pdf> |
|  |  | Keeping your body clean and health | <https://www.planetpuberty.org.au/wp-content/uploads/2021/02/pp-keeping-the-body-hygienic.pdf> |
|  |  | Period product options | <https://www.planetpuberty.org.au/wp-content/uploads/2021/10/pp-period-product-options.pdf> |
|  |  | Acne and Pimples | <https://www.planetpuberty.org.au/wp-content/uploads/2020/11/pp-acne-and-pimples.pdf> |
|  |  | Cleaning your vulva and vagina | <https://www.planetpuberty.org.au/wp-content/uploads/2020/11/pp-cleaning-your-vulva-and-vagina.pdf> |
|  |  | Talking About Periods | [pp-talking-about-periods.pdf (planetpuberty.org.au)](https://www.planetpuberty.org.au/wp-content/uploads/2020/11/pp-talking-about-periods.pdf) |
|  |  | Teaching About Differences Between Girls’ and Boys’ Bodies | [pp-voice-breaking.pdf (planetpuberty.org.au)](https://www.planetpuberty.org.au/wp-content/uploads/2021/02/pp-voice-breaking.pdf) |
|  |  | Using a pad | [PP-changing-a-pad.pdf (planetpuberty.org.au)](https://www.planetpuberty.org.au/wp-content/uploads/2020/11/PP-changing-a-pad.pdf) |
|  |  | Using a Tampon | [planetpuberty.org.au/wp-content/uploads/2020/11/pp-using-a-tampon.pdf](https://www.planetpuberty.org.au/wp-content/uploads/2020/11/pp-using-a-tampon.pdf) |
|  |  | What is a Normal Period? | [PP-normal-period.pdf (planetpuberty.org.au)](https://www.planetpuberty.org.au/wp-content/uploads/2022/02/PP-normal-period.pdf) |
| Positive Partnerships | [https://www.positivepartnerships.com.au/](https://www.secca.org.au/) | health and hygiene in the home | [Positive Partnerships \| Health & Hygiene in the Home](https://www.positivepartnerships.com.au/resources/practical-tools-information-sheets/health-hygiene-in-the-home) |
|  |  | wellbeing and resilience | [Positive Partnerships \| Wellbeing and Resilience](https://www.positivepartnerships.com.au/resources/practical-tools-information-sheets/wellbeing-and-resilience) |
|  |  | Talking about Sexuality | [Positive Partnerships \| Talking about Sexuality](https://www.positivepartnerships.com.au/resources/practical-tools-information-sheets/talking-about-sexuality) |
|  |  | Who Sees Our Private Parts (Female) | <https://www.positivepartnerships.com.au/uploads/PDF-files/Who-Sees-Our-Private-Parts-Female-Social-Story-Booklet.pdf> |
|  |  | Who Sees Our Private Parts (Male) | <https://www.positivepartnerships.com.au/uploads/PDF-files/Who-Sees-Our-Private-Parts-Male-Social-Story-Booklet.pdf> |
|  |  | Interoception | [Positive Partnerships \| Interoception](https://www.positivepartnerships.com.au/resources/practical-tools-information-sheets/interoception) |
|  |  | Interoception webinar | https://vimeo.com/854497743 |
|  |  | Practical Application of Interoception | <https://vimeo.com/854512702> |
|  |  | Interoception and self-regulation | https://studentwellbeinghub.edu.au/media/erkfigg4/swh_getreadytolearn_selfregulationresource-2023.pdf |
| Queensland Centre for Intellectual and Developmental Disability | [Queensland Centre for Intellectual and Developmental Disability - University of Queensland (uq.edu.au)](https://qcidd.centre.uq.edu.au/) | CT head scan - People with intellectual disabilities (pictures) | https://qcidd.centre.uq.edu.au/files/2020/CT%20head%20scan%20PWID%20with%20pics%281%29.pdf |
|  |  | CT head scan - People with intellectual disabilities (nopictures) | https://qcidd.centre.uq.edu.au/files/2017/CT%20head%20Scan%20PWID%20no%20pics.pdf |
|  |  | CT head scan with dye - People with intellectual disabilities (pictures) | https://qcidd.centre.uq.edu.au/files/1999/CT%20Head%20Scan%20dye_with_Pics_PWID.pdf |
|  |  | CT head scan with dye - People with intellectual disabilities (nopictures) | https://qcidd.centre.uq.edu.au/files/2008/CT%20Head%20Scan%20no%20pics%20PWID_Dye%281%29.pdf |
|  |  | CT head scan - For carers (pictures) | https://qcidd.centre.uq.edu.au/files/2023/CT%20Head%20Scan%20with%20pics_Carers_Dye%281%29.pdf |
|  |  | CT head scan - For carers (no pictures) | https://qcidd.centre.uq.edu.au/files/2014/CT%20Head%20Scan%20no%20pics_Carers_No%20dye.pdf |
|  |  | CT head scan with dye - For carers (pictures) | https://qcidd.centre.uq.edu.au/files/2023/CT%20Head%20Scan%20with%20pics_Carers_Dye%281%29.pdf |
|  |  | CT head scan with dye- For carers (no pictures) | https://qcidd.centre.uq.edu.au/files/2011/CT%20Head%20Scan%20no%20pics_Carers_Dye.pdf |
|  |  | What is a CT head scan? | https://qcidd.centre.uq.edu.au/what-ct-head-scan |
|  |  | Frequently asked questions about CT head scan | <https://qcidd.centre.uq.edu.au/files/2026/CT%20Head%20Scan_FAQ%281%29.pdf> |
|  |  | CT Head Scan | [CT Head Scan - Queensland Centre for Intellectual and Developmental Disability - University of Queensland (uq.edu.au)](https://qcidd.centre.uq.edu.au/ct-head-scan) |
|  |  | Blood Test - For people with intellectual disabilities (pictures) | https://qcidd.centre.uq.edu.au/files/127/Blood_Test_PWID_Pics.pdf |
|  |  | Blood Test - For people with intellectual disabilities (no pictures) | https://qcidd.centre.uq.edu.au/files/124/Blood_Test_PWID-no%20pics.pdf |
|  |  | Blood Test - For carers (pictures) | https://qcidd.centre.uq.edu.au/files/121/Blood_Test_Carers_Pics.pdf |
|  |  | Blood Test - For carers (no pictures) | https://qcidd.centre.uq.edu.au/files/118/Blood_Test_Carers_no-pics.pdf |
|  |  | What is a blood test | https://qcidd.centre.uq.edu.au/what-blood-test |
|  |  | Frequently Asked Questions about Blood Test | <https://qcidd.centre.uq.edu.au/frequently-asked-questions-about-blood-test> |
|  |  | Blood Test | [Blood Tests - Queensland Centre for Intellectual and Developmental Disability - University of Queensland (uq.edu.au)](https://qcidd.centre.uq.edu.au/blood-tests) |
|  |  | Abdomninal Xray | [Abdominal XRay - Queensland Centre for Intellectual and Developmental Disability - University of Queensland (uq.edu.au)](https://qcidd.centre.uq.edu.au/abdominal-xray) |
|  |  | What is an Abdominal X-Ray?: For people with intellectual disabilities (with pictures - not standing) | https://qcidd.centre.uq.edu.au/files/85/Abdo_XRay_Steps_Pictures_Nonerect_PWID.pdf |
|  |  | What is an Abdominal X-Ray?: For people with intellectual disabilities (without pictures - not standing) | https://qcidd.centre.uq.edu.au/files/76/Abdo_XRay_Steps_Only_Nonerect_PWID.pdf |
|  |  | What is an Abdominal X-Ray?: For people with intellectual disabilities (with pictures- standing) | https://qcidd.centre.uq.edu.au/files/79/Abdo_XRay_Steps_Pictures_Erect_PWID.pdf |
|  |  | What is an Abdominal X-Ray?: For people with intellectual disabilities (without pictures- standing) | https://qcidd.centre.uq.edu.au/files/370/What_Is_An_Abdo_Xray.pdf |
|  |  | What is an Abdominal X-Ray?: For caregivers (with pictures - not standing) | https://qcidd.centre.uq.edu.au/files/82/Abdo_XRay_Steps_Pictures_Nonerect_Carers.pdf |
|  |  | What is an Abdominal X-Ray?: For caregivers (without pictures - not standing) | https://qcidd.centre.uq.edu.au/files/61/Abdo_XRay%20no%20pics_Nonerect_Carers.pdf |
|  |  | What is an Abdominal X-Ray?: For caregivers (with pictures- standing) | https://qcidd.centre.uq.edu.au/files/73/Abdo_XRay_Pictures_Erect_Carers.pdf |
|  |  | What is an Abdominal X-Ray?: For caregivers (without pictures- standing) | https://qcidd.centre.uq.edu.au/files/58/Abdo_XRay%20no%20pics_Erect_Carers.pdf |
|  |  | Frequently Asked Questions about Abdominal X-Rays | https://qcidd.centre.uq.edu.au/frequently-asked-questions-about-abdominal-x-rays |
|  |  | What is an Abdominal X-Ray? | https://qcidd.centre.uq.edu.au/what-abdominal-x-ray |
|  |  | Diabetes for Support People | [https://www.qcidd.com.au/home/individuals-2/diabetes-to-the-point/diabetes-for-support-people//](https://www.qcidd.com.au/home/individuals-2/diabetes-to-the-point/diabetes-for-support-people/) |
|  |  | Diabetes Overview | <https://www.qcidd.com.au/home/individuals-2/diabetes-to-the-point/diabetes-for-support-people/diabetes-overview/> |
|  |  | Blood Sugar | <https://www.qcidd.com.au/home/individuals-2/diabetes-to-the-point/diabetes-for-support-people/blood-glucose/> |
|  |  | Health and Medical | <https://www.qcidd.com.au/home/individuals-2/diabetes-to-the-point/diabetes-for-support-people/health-and-medical/> |
|  |  | Food and Exercise | <https://www.qcidd.com.au/home/individuals-2/diabetes-to-the-point/diabetes-for-support-people/food-and-exercise/> |
|  |  | Sick Days and Holidays | <https://www.qcidd.com.au/home/individuals-2/diabetes-to-the-point/diabetes-for-support-people/sick-days-and-holidays/> |
|  |  | Living with Diabetes | [ER Diabetes - QCIDD](https://www.qcidd.com.au/home/individuals-2/diabetes-to-the-point/diabetes/) |
|  |  | About Diabetes | <https://www.qcidd.com.au/diabetes/about-diabetes/what-is-diabetes/> |
|  |  | Type 1 Diabetes | <https://www.qcidd.com.au/diabetes/about-diabetes/type-1-diabetes/> |
|  |  | Type 2 Diabetes | <https://www.qcidd.com.au/diabetes/about-diabetes/type-2-diabetes/> |
|  |  | Blood Sugars | <https://www.qcidd.com.au/diabetes/blood-sugar/> |
|  |  | Insulin | <https://www.qcidd.com.au/diabetes/insulin-3/> |
|  |  | Exercise | <https://www.qcidd.com.au/diabetes/exercise/> |
|  |  | Sick days | <https://www.qcidd.com.au/diabetes/sick-days/> |
|  |  | Visiting health workers | <https://www.qcidd.com.au/diabetes/visiting-health-professionals/> |
|  |  | Health Problems | <https://www.qcidd.com.au/diabetes/health-problems-from-diabetes/> |
|  |  | The Ask Health Diary | [The Ask Health Diary - Queensland Centre for Intellectual and Developmental Disability - University of Queensland (uq.edu.au)](https://qcidd.centre.uq.edu.au/resources/ask-health-diary) |
| Queensland Department of Health | <https://www.health.qld.gov.au/> | Physical Health | [Physical health \| People with disability \| Queensland Government (www.qld.gov.au)](https://www.qld.gov.au/disability/adults/mental-health/physical-health) |
|  |  | Julian's Key Health Passport | Julian's Key Health Passport |
|  |  | Ryan's Rule | [Ryan's Rule \| Health and wellbeing \| Queensland Government (www.qld.gov.au)](https://www.qld.gov.au/health/support/shared-decision-making/ryans-rule) |
|  |  | Mental Health | [Mental health \| People with disability \| Queensland Government (www.qld.gov.au)](https://www.qld.gov.au/disability/adults/mental-health/mental-health) |
| Queensland Government | <https://www.qld.gov.au/> | People with disability — COVID-19 | <https://www.qld.gov.au/disability/covid-19-coronavirus/covid-19-information-for-people-with-disability2> |
|  |  | What to do if you test positive for COVID-19 | <https://qdn.org.au/wp-content/uploads/2022/02/COVID-19_InfoSheet_2.pdf> |
|  |  | Getting the COVD care you need | <https://qdn.org.au/wp-content/uploads/2022/02/COVID-19_InfoSheet_1.pdf> |
|  |  | COVID-19 care pathway process | <https://qdn.org.au/wp-content/uploads/2022/02/COVID-19_InfoSheet_3.pdf> |
|  |  | Know Your Rights | <https://qdn.org.au/wp-content/uploads/2021/07/KnowYourRights_Guide_full_FINAL.pdf> |
|  |  | Person-Centred Emergency Preparedness (P-CEP) Workbook | <https://qdn.org.au/wp-content/uploads/2021/08/2020-08-19-Person-Centred-Emergency-Preparedness-P-CEP-WORKBOOK_FORM_FINAL.pdf> |
| Raising Children | [https://raisingchildren.net.au](https://raisingchildren.net.au/) | Physical activity for children and teenagers with disability | <https://raisingchildren.net.au/disability/mental-health-physical-health/children-with-disability-physical-health/physical-activity-children-with-disability> |
|  |  | Autism: Health & Wellbeing | [Autism: child health & wellbeing \| Raising Children Network](https://raisingchildren.net.au/autism/health-wellbeing) |
|  |  | Depression: teenagers with diability or chronic conditions | https://raisingchildren.net.au/disability/mental-health-physical-health/depression/depression-teens-with-disability |
|  |  | Depression: children with disability and chronic conditions 5-11 years | https://raisingchildren.net.au/disability/mental-health-physical-health/depression/depression-children-with-disability |
|  |  | Anxiety: children with disability or chronic conditions 5-11 years | https://raisingchildren.net.au/disability/mental-health-physical-health/anxiety/anxiety-children-with-disability |
|  |  | Anxiety: teenagers with disability or chronic conditions | <https://raisingchildren.net.au/disability/mental-health-physical-health/anxiety/anxiety-teens-with-disability> |
|  |  | Sexuality and sexual development: autistic teenagers | <https://raisingchildren.net.au/autism/development/sexual-development/sexuality-teens-with-asd> |
| SeeAbility | <https://www.seeability.org/> | Having An Eye Test | [Having an eye test (seeability.org)](https://www.seeability.org/sites/default/files/2022-05/Having%20an%20eye%20test%20-%20April%2022.pdf) |
| Sexual Health Victoria | <https://shvic.org.au/> | Sexual Health in Plain English - Factsheets | [Plain English Sexual Health Factsheets \| Information and Support \| Sexual Health Victoria - Sexual Health Victoria (shvic.org.au)](https://shvic.org.au/for-you/resources/sexual-health-plain-english-factsheets) |
|  |  | Easy English Factsheets - With Illustrations | [Easy English Sexual Health \| Factsheets \| Sexual Health Victoria - Sexual Health Victoria (shvic.org.au)](https://shvic.org.au/for-you/resources/easy-english) |
|  |  | New Words to Know | https://shvic.org.au/assets/resources/2023-SHV-New-Words-to-Know-2.03.2023.pdf |
|  |  | Advocacy Services | https://shvic.org.au/assets/img/content/Advocacy-Services-List.pdf |
|  |  | Disability Complaint Information | https://shvic.org.au/assets/img/content/DisabilityComplaintInformation.pdf |
|  |  | What is Sex | https://shvic.org.au/assets/resources/2023-SHV-What-is-Sex-02.03.2023.pdf |
|  |  | Safer Sex | <https://shvic.org.au/assets/resources/2023-SHV-Safer-Sex-2.03.2023.pdf> |
|  |  | Contraception | https://shvic.org.au/assets/resources/2023-SHV-Contraception-2.03.2023.pdf |
|  |  | Contraceptive Options | https://shvic.org.au/assets/resources/2023-SHV-Contraceptive-Options-What-can-I-choose-March-2023.pdf |
|  |  | Unplanned Pregnancy | https://shvic.org.au/assets/resources/2023-SHV-Unplanned-pregnancy-7.03.2023.pdf |
|  |  | Medical Terminations of Pregnancy | https://shvic.org.au/assets/resources/2023-MTOP-02.03.2023.pdf |
|  |  | Diversity of LGBTIQ | https://shvic.org.au/assets/resources/2023-SHV-Diversity-and-LGBTIQ-2.03.2023.pdf |
|  |  | Get a Test for STI and BBV | https://shvic.org.au/assets/resources/2023-SHV-Get-a-test-for-STI-and-BBV-2.03.2023.pdf |
|  |  | STI & BBV The Facts | https://shvic.org.au/assets/resources/2023-SHV-STI-and-BBV-The-Facts-02.03.2023.pdf |
|  |  | Human Papilloma Virus | https://shvic.org.au/assets/resources/2023-SHV-Human-Papillomavirus-13.12.2023.pdf |
|  |  | Sex and the Law | https://shvic.org.au/assets/resources/2023-SHV-Sex-and-the-Law-02.03.2023.pdf |
|  |  | Cervical Screening Tests: the plain facts | https://shvic.org.au/assets/resources/cervical-screening-tests-plain-facts.pdf |
|  |  | Our SHV Clinics | https://shvic.org.au/assets/resources/2023-SHV-Our-SHV-Clinics-7.3.2023.pdf |
| Sexuality Education Counselling and Consultancy Agency (SECCA) | [https://www.secca.org.au](https://www.secca.org.au/) | Resources | <https://www.secca.org.au/resources/> |
|  |  | Sexuality, Relationships and Your Rights | https://app.box.com/s/zfqqckm5any15v8ejdrpr0tiwrl7yvvl/file/1389527425951 |
|  |  | Introduction to Consent | https://www.secca.org.au/wp-content/uploads/2024/06/Introduction-to-Consent-Book-1-Web-DPS.pdf |
|  |  | Introduction to Sexual Consent | https://www.secca.org.au/wp-content/uploads/2024/06/Introduction-to-Sexual-Consent-Book-2-Web-SPS.pdf |
|  |  | Cervical Screening Test Brochure | https://www.secca.org.au/resources/your-guide-to-cervical-screening/ |
|  |  | Your Guide to Cervical Screening Book | https://www.secca.org.au/resources/your-guide-to-cervical-screening/ |
|  |  | Wanted and Unwanted Touching | <https://www.secca.org.au/wp-content/uploads/2022/08/SECCA-Feel-Safe-Brochure-Touch-2pp-WEB.pdf> |
|  |  | Gender Diversity | https://www.secca.org.au/wp-content/uploads/2022/05/SEC1000-Gender-Diversity-Brochure-v4.pdf |
|  |  | Online Dating | https://www.secca.org.au/wp-content/uploads/2022/08/SECCA-Feel-Safe-Brochure-Online-Dating-2pp-WEB.pdf |
|  |  | Things to Know About Porn | https://www.secca.org.au/wp-content/uploads/2024/04/SECCA-Feel-Safe-Brochure-Porn-6pp-WEB-V2.pdf |
|  |  | Teaching Public and Private | https://www.secca.org.au/resources/teaching-public-and-private/ |
|  |  | Early Warning Signs | <https://www.secca.org.au/resources/early-warning-signs/> |
|  |  | Protective Behaviours | <https://www.secca.org.au/resources/protective-behaviours/> |
|  |  | Sexting | <https://www.secca.org.au/resources/sexting-brochure/> |
|  |  | Safer Sex | <https://www.secca.org.au/resources/safe-sex-brochure/> |
|  |  | Internet Safety Cards | <https://www.secca.org.au/resources/internet-safety-cards/> |
| South Australian Council on Intellectual Disability (SACID) | <https://sacid.org.au/> | Strong Mind Series Bundle (5 Resources) | [Strong Mind Series Bundle (5 Resources) – South Australian Council on Intellectual Disability \| SACID](https://sacid.org.au/product/strong-mind-bundle/) |
|  |  | Sexuality and Relationships | [Sexuality and Relationships Education (SA) – South Australian Council on Intellectual Disability \| SACID](https://sacid.org.au/product/sexuality-and-relationships-education-handout/) |
| South Eastern Centre Against Sexual Assault & Family Violence (SECASA) | <https://www.secasa.org.au/> | Health Check after sexual assault | [health check after sexual assault (secasa.org.au)](https://www.secasa.org.au/wp-content/uploads/2020/10/health-check-after-sexual-assault.pdf) |
| South Eastern Sydney Local Health District (SESLHD) | https://a2d.healthcare/a2d | The A2D Together Folder for People with a Disability | [The A2D Together Folder improves the hospital experience for people with intellectual disability](http://a2d.healthcare/a2d-in-easy-english/) |
| South West Autism Network (SWAN) | <https://www.swanautism.org.au/> | (Easy English Factsheet) | [Health (13-18 years) - SWAN (swanautism.org.au)](https://www.swanautism.org.au/asset/health-12-18-years/) |
|  |  | Health (0-6 years) | [Health (0-6 years) - SWAN (swanautism.org.au)](https://www.swanautism.org.au/asset/health-0-6-years/#general-practitioners-(gp)) |
|  |  | Health (7-12 years) | [Health (7-12 years) - SWAN (swanautism.org.au)](https://www.swanautism.org.au/asset/health-7-12-years/#puberty) |
|  |  | Health (18 years +) | [Health (18 years +) - SWAN (swanautism.org.au)](https://www.swanautism.org.au/asset/health-18-years/) |
| Spectrom | <https://spectrom.wixsite.com/project> | Easy read medication leaflets | [Easy read medication leaflets \| Project (spectrom.wixsite.com)](https://spectrom.wixsite.com/project/easy-read-medication-leaflets) |
|  |  | Amisulpride | <https://0fbd91af-d847-4ca7-821e-e16b585f862f.filesusr.com/ugd/5a1a0e_e9aed68af32948f7b88171bd7493c2b8.pdf> |
|  |  | Aripiprazole | [5a1a0e_fe19734eea4a426d9e848fd2123f5feb.pdf (filesusr.com)](https://0fbd91af-d847-4ca7-821e-e16b585f862f.filesusr.com/ugd/5a1a0e_fe19734eea4a426d9e848fd2123f5feb.pdf) |
|  |  | Benzodiazepines | <https://0fbd91af-d847-4ca7-821e-e16b585f862f.filesusr.com/ugd/5a1a0e_fe19734eea4a426d9e848fd2123f5feb.pdf> |
|  |  | Carbamazepine | [5a1a0e_9d1561b14b714e648c7d55396c3043bc.pdf (filesusr.com)](https://0fbd91af-d847-4ca7-821e-e16b585f862f.filesusr.com/ugd/5a1a0e_9d1561b14b714e648c7d55396c3043bc.pdf) |
|  |  | Chlorpromazine | <https://0fbd91af-d847-4ca7-821e-e16b585f862f.filesusr.com/ugd/5a1a0e_49412977d58746c7bfb9909b7b0584f6.pdf> |
|  |  | Diazepam | [5a1a0e_828f91c7a56c49f388142848a3a54a41.pdf (filesusr.com)](https://0fbd91af-d847-4ca7-821e-e16b585f862f.filesusr.com/ugd/5a1a0e_828f91c7a56c49f388142848a3a54a41.pdf) |
|  |  | Citalopram | [5a1a0e_48445455df904328a531e6d0e956617f.pdf (filesusr.com)](https://0fbd91af-d847-4ca7-821e-e16b585f862f.filesusr.com/ugd/5a1a0e_48445455df904328a531e6d0e956617f.pdf) |
|  |  | Escitalopram | [5a1a0e_fcd10f50a46549839147deaa6371353b.pdf (filesusr.com)](https://0fbd91af-d847-4ca7-821e-e16b585f862f.filesusr.com/ugd/5a1a0e_fcd10f50a46549839147deaa6371353b.pdf) |
|  |  | Fluoxetine | [5a1a0e_d575becdb17d4a2b8973043c2fe48e45.pdf (filesusr.com)](https://0fbd91af-d847-4ca7-821e-e16b585f862f.filesusr.com/ugd/5a1a0e_d575becdb17d4a2b8973043c2fe48e45.pdf) |
|  |  | Flupentixol | [5a1a0e_9c79c37b52db42a9ac08ba229774c39b.pdf (filesusr.com)](https://0fbd91af-d847-4ca7-821e-e16b585f862f.filesusr.com/ugd/5a1a0e_9c79c37b52db42a9ac08ba229774c39b.pdf) |
|  |  | Fluvoxamine | [5a1a0e_5b7fc242a36f48fb8b1720e6ea104843.pdf (filesusr.com)](https://0fbd91af-d847-4ca7-821e-e16b585f862f.filesusr.com/ugd/5a1a0e_5b7fc242a36f48fb8b1720e6ea104843.pdf) |
|  |  | Haloperidol | <https://0fbd91af-d847-4ca7-821e-e16b585f862f.filesusr.com/ugd/5a1a0e_90a9f4bee8b14830bc62be9379238aa7.pdf> |
|  |  | Lamotrigine | <https://0fbd91af-d847-4ca7-821e-e16b585f862f.filesusr.com/ugd/5a1a0e_aaaf5d6deccb4b78886e8d4c0a926e13.pdf> |
|  |  | Levetiracetam | <https://0fbd91af-d847-4ca7-821e-e16b585f862f.filesusr.com/ugd/5a1a0e_f19b31bbf2d94e7fba781130db5e7355.pdf> |
|  |  | Lithium | <https://0fbd91af-d847-4ca7-821e-e16b585f862f.filesusr.com/ugd/5a1a0e_cc22ed9182af4dc1bf6f89fa59ea6391.pdf> |
|  |  | Lorazepam | [5a1a0e_fd0898763dc1452b9ac29e9b946af4a4.pdf (filesusr.com)](https://0fbd91af-d847-4ca7-821e-e16b585f862f.filesusr.com/ugd/5a1a0e_fd0898763dc1452b9ac29e9b946af4a4.pdf) |
|  |  | Methylphenidate | [5a1a0e_f55c4980c49e48b6a14b130c9d018180.pdf (filesusr.com)](https://0fbd91af-d847-4ca7-821e-e16b585f862f.filesusr.com/ugd/5a1a0e_f55c4980c49e48b6a14b130c9d018180.pdf) |
|  |  | Mirtazapine | [5a1a0e_620b638ddae44017a570aefd18813957.pdf (filesusr.com)](https://0fbd91af-d847-4ca7-821e-e16b585f862f.filesusr.com/ugd/5a1a0e_620b638ddae44017a570aefd18813957.pdf) |
|  |  | Olanzapine | [5a1a0e_d5f2ac98f67d46b7a7511a8328348af9.pdf (filesusr.com)](https://0fbd91af-d847-4ca7-821e-e16b585f862f.filesusr.com/ugd/5a1a0e_d5f2ac98f67d46b7a7511a8328348af9.pdf) |
|  |  | Paroxetine | [5a1a0e_913013b45cd54ccc82b87240e1c4bb2b.pdf (filesusr.com)](https://0fbd91af-d847-4ca7-821e-e16b585f862f.filesusr.com/ugd/5a1a0e_913013b45cd54ccc82b87240e1c4bb2b.pdf) |
|  |  | Quetiapine | [5a1a0e_d2ee342bb9b54dc88efe4a5fdd396d75.pdf (filesusr.com)](https://0fbd91af-d847-4ca7-821e-e16b585f862f.filesusr.com/ugd/5a1a0e_d2ee342bb9b54dc88efe4a5fdd396d75.pdf) |
|  |  | Risperidone | [5a1a0e_5500c868d7614f1c8e387b1e16a856b0.pdf (filesusr.com)](https://0fbd91af-d847-4ca7-821e-e16b585f862f.filesusr.com/ugd/5a1a0e_5500c868d7614f1c8e387b1e16a856b0.pdf) |
|  |  | Sertraline | [5a1a0e_dc38906e3df94c70839e6561fa878422.pdf (filesusr.com)](https://0fbd91af-d847-4ca7-821e-e16b585f862f.filesusr.com/ugd/5a1a0e_dc38906e3df94c70839e6561fa878422.pdf) |
|  |  | Sulpiride | [5a1a0e_3a4fe955b87a42e08f8740be10fd1ce2.pdf (filesusr.com)](https://0fbd91af-d847-4ca7-821e-e16b585f862f.filesusr.com/ugd/5a1a0e_3a4fe955b87a42e08f8740be10fd1ce2.pdf) |
|  |  | Topiramate | [5a1a0e_7cbb98f82ed04ae58c5b2b50c66046c4.pdf (filesusr.com)](https://0fbd91af-d847-4ca7-821e-e16b585f862f.filesusr.com/ugd/5a1a0e_7cbb98f82ed04ae58c5b2b50c66046c4.pdf) |
|  |  | Trifluoperazine | [5a1a0e_09ad0e7796c746a9bc9d05444e187308.pdf (filesusr.com)](https://0fbd91af-d847-4ca7-821e-e16b585f862f.filesusr.com/ugd/5a1a0e_09ad0e7796c746a9bc9d05444e187308.pdf) |
|  |  | Valproate | [5a1a0e_e25e26abf4634ee58b0b9d569c9c43e4.pdf (filesusr.com)](https://0fbd91af-d847-4ca7-821e-e16b585f862f.filesusr.com/ugd/5a1a0e_e25e26abf4634ee58b0b9d569c9c43e4.pdf) |
|  |  | Venlafaxine | [5a1a0e_510acf1c73024366ad1bab60071c1416.pdf (filesusr.com)](https://0fbd91af-d847-4ca7-821e-e16b585f862f.filesusr.com/ugd/5a1a0e_510acf1c73024366ad1bab60071c1416.pdf) |
|  |  | Zuclopentixol | [5a1a0e_5c54b6e5a1f34188aeb6f5501ae061ea.pdf (filesusr.com)](https://0fbd91af-d847-4ca7-821e-e16b585f862f.filesusr.com/ugd/5a1a0e_5c54b6e5a1f34188aeb6f5501ae061ea.pdf) |
|  |  | Zuclopentixol Injection | [5a1a0e_ef8112e8fae84b54a45fbb4434e3d298.pdf (filesusr.com)](https://0fbd91af-d847-4ca7-821e-e16b585f862f.filesusr.com/ugd/5a1a0e_ef8112e8fae84b54a45fbb4434e3d298.pdf) |
| Surrey Place | <https://ddprimarycare.surreyplace.ca/> | Physical Health | [Physical Health – DDPCP (surreyplace.ca)](https://ddprimarycare.surreyplace.ca/tools-2/physical-health/) |
|  |  | Seizures: Tips for Caregivers Surrey Place Developmental | <https://ddprimarycare.surreyplace.ca/wp-content/uploads/2019/06/3.7.1-Seizures-Tips-for-Caregivers.pdf> |
|  |  | Seizure Action Plan | <https://ddprimarycare.surreyplace.ca/wp-content/uploads/2019/06/3.7.2-Seizures-Action-Plan.pdf> |
|  |  | Seizure Record To Establish Baseline | [3.7.3-Seizures-Baseline-Record.pdf (surreyplace.ca)](https://ddprimarycare.surreyplace.ca/wp-content/uploads/2019/06/3.7.3-Seizures-Baseline-Record.pdf) |
|  |  | Seizures: General Information | <https://ddprimarycare.surreyplace.ca/wp-content/uploads/2019/06/3.7.4-Seizures-General-Information.pdf> |
|  |  | Monitoring Chart: Seizures Yearly Frequency Surrey Place Developmental | [3.7.5-Seizures-Monitoring-Chart-Yearly.pdf (surreyplace.ca)](https://ddprimarycare.surreyplace.ca/wp-content/uploads/2019/06/3.7.5-Seizures-Monitoring-Chart-Yearly.pdf) |
|  |  | Seizures: First Aid | <https://ddprimarycare.surreyplace.ca/wp-content/uploads/2019/06/3.7.7-Seizures-First-Aid.pdf> |
|  |  | Direct Observation System (DOS) | [3.11-Direct-Observation-System-1.pdf (surreyplace.ca)](https://ddprimarycare.surreyplace.ca/wp-content/uploads/2019/06/3.11-Direct-Observation-System-1.pdf) |
|  |  | Monitoring Chart: Bowel Movement (BM) | <https://ddprimarycare.surreyplace.ca/wp-content/uploads/2019/05/Bowel-Movement-monitoring-chart_final.pdf> |
|  |  | Monitoring Chart: Menstrual Cycle | <https://ddprimarycare.surreyplace.ca/wp-content/uploads/2019/05/Menstrual-Cycle-monitoring-chart_final.pdf> |
|  |  | Pain Assessment | [May 16_Pain Assessment (surreyplace.ca)](https://ddprimarycare.surreyplace.ca/wp-content/uploads/2019/06/3.8-Pain-Assessment.pdf) |
|  |  | Monitoring Chart: Sleep (24-hour sleep record) | [Sleep-monitoring-chart_final.pdf (surreyplace.ca)](https://ddprimarycare.surreyplace.ca/wp-content/uploads/2019/05/Sleep-monitoring-chart_final.pdf) |
|  |  | Monitoring Chart: Weight (Adults) | <https://ddprimarycare.surreyplace.ca/wp-content/uploads/2019/05/Weight-monitoring-chart_final.pdf> |
|  |  | Monitoring Chart: Food Diary (Weekly) | [3.6-Food-Weekly-Monitoring-Chart-updated.pdf (surreyplace.ca)](https://ddprimarycare.surreyplace.ca/wp-content/uploads/2019/05/3.6-Food-Weekly-Monitoring-Chart-updated.pdf) |
|  |  | Monitoring Chart: Diabetes Blood Sugar Diary | [3.5-Blood-Sugar-Monitoring-Chart-2.pdf (surreyplace.ca)](https://ddprimarycare.surreyplace.ca/wp-content/uploads/2019/05/3.5-Blood-Sugar-Monitoring-Chart-2.pdf) |
|  |  | Monitoring Chart: Food Diary (Daily) | <https://ddprimarycare.surreyplace.ca/wp-content/uploads/2019/05/3.6-Food-Daily-Monitoring-Chart-2.pdf> |
|  |  | Mental Health | [Mental Health – DDPCP (surreyplace.ca)](https://ddprimarycare.surreyplace.ca/tools-2/mental-health/) |
|  |  | Approaches to Care and General Considerations | [Approaches to Care & General Considerations – DDPCP (surreyplace.ca)](https://ddprimarycare.surreyplace.ca/tools-2/general-health/) |
|  |  | My Health Care Visit | http://ddprimarycare.surreyplace.ca/wp-content/uploads/2019/08/5.2-My-Healthcare-Visit.pdf |
| Telethon Kids Institute | [Telethon Kids Institute](https://www.telethonkids.org.au/) | ActiveRett | [ActiveRett \| Telethon Kids Institute](https://rett.telethonkids.org.au/resources/activerett/) |
|  |  | What is Rett syndrome? | [About Rett syndrome \| The Kids Research Institute Australia](https://rett.thekids.org.au/resources/activerett/about-rett-syndrome/) |
|  |  | Why get active? | <https://rett.thekids.org.au/resources/activerett/why-get-active/> |
|  |  | Planning opportunities for uptime | <https://rett.thekids.org.au/resources/activerett/how-to-get-active/planning-opportunities-for-uptime/> |
|  |  | Routines to maximise uptime | [Routines to maximise uptime \| Rett syndrome (thekids.org.au)](https://rett.thekids.org.au/resources/activerett/how-to-get-active/routines-to-maximise-uptime/) |
|  |  | Toolkit of uptime stratigies | [Toolkit of uptime strategies \| Rett syndrome (thekids.org.au)](https://rett.thekids.org.au/resources/activerett/how-to-get-active/toolkit-of-uptime-strategies/) |
|  |  | How much activity is recommended? | [How much activity is recommended? \| Rett syndrome (thekids.org.au)](https://rett.thekids.org.au/resources/activerett/how-active-to-get/) |
|  |  | Equipment to support standing activities | <https://rett.thekids.org.au/resources/activerett/equipment-to-support-physical-activity/> |
|  |  | Scoliosis in Rett Syndrome | [scoliosis_booklet.pdf (telethonkids.org.au)](https://rett.telethonkids.org.au/siteassets/media-docs---rett-syndrome/scoliosis_booklet.pdf) |
|  |  | Improving health outcomes in Rett Syndrome: Nutritional and Digestive Health | [growth_and_nutrition-booklet.pdf (telethonkids.org.au)](https://rett.telethonkids.org.au/siteassets/media-docs---rett-syndrome/growth_and_nutrition-booklet.pdf) |
| Turning Point | <https://www.turning-point.co.uk/> | Healthy Eating | <https://mytp.me/tp-fom-eat-web> |
|  |  | Physical Activity | <https://mytp.me/tp-fom-activity-web> |
|  |  | Sleep Difficulties | <https://mytp.me/tp-fom-sleep-web> |
|  |  | Alcohol | <https://mytp.me/tp-fom-alcohol-web> |
|  |  | Benzodiazepines | <https://mytp.me/tp-fom-benzos-web> |
|  |  | Amphetamines | <https://mytp.me/tp-fom-amphetamines-web> |
|  |  | Cannabis | <https://mytp.me/tp-fom-cannabis-web> |
|  |  | Cocaine | <https://mytp.me/tp-fom-cocaine-web> |
|  |  | Ecstacy | <https://mytp.me/tp-fom-ecstasy-web> |
|  |  | GHB | <https://mytp.me/tp-fom-ghbgbl-web> |
|  |  | Ketamine | <https://mytp.me/tp-fom-ketamine-web> |
|  |  | 5 Ways to Wellbeing | <https://mytp.me/tp-fom-5ways-web> |
|  |  | Low Mood | <https://mytp.me/tp-fom-mood-web> |
|  |  | Managing Worries | <https://mytp.me/tp-fom-managing-worries-web> |
|  |  | Stress at Work | <https://mytp.me/tp-fom-stress-web> |
|  |  | Gambling | <https://mytp.me/tp-fom-gambling-web> |
|  |  | Anxiety | <https://mytp.me/tp-fom-anxiety-web> |
| Victorian Advocacy League for Individuals with Disability (VALID) | <https://www.valid.org.au/> | Palliative Care Easy Read Resources | [Palliative Care Easy Read Resources - VALID](https://valid.org.au/ppalliative-carew-easy-read-resources-nbsp/) |
|  |  | Breast Cancer Screening | [Breast-Screen-Poster-1.pdf (valid.org.au)](https://valid.org.au/wp-content/uploads/2020/07/Breast-Screen-Poster-1.pdf) |
|  |  | Cervical Cancer Screening | [Cervical-Screen-Poster.pdf (valid.org.au)](https://valid.org.au/wp-content/uploads/2020/07/Cervical-Screen-Poster.pdf) |
|  |  | Bowel Screening Information | [Bowel-Screening-Poster-.pdf (valid.org.au)](https://valid.org.au/wp-content/uploads/2020/05/Bowel-Screening-Poster-.pdf) |
|  |  | Easy English What is Coronavirus | [Easy-English-What-is-Coronavirus-July-2020.pdf (valid.org.au)](https://valid.org.au/wp-content/uploads/2020/07/Easy-English-What-is-Coronavirus-July-2020.pdf) |
|  |  | Do I have Coronavirus | [Do-I-have-Coronavirus-chart-Final.pdf (valid.org.au)](https://valid.org.au/wp-content/uploads/2020/07/Do-I-have-Coronavirus-chart-Final.pdf) |
| WA Health | <https://www.health.wa.gov.au/> | Hospital Stay Guidelines: A guide for people with disability, families, friends and carers | <https://www.health.wa.gov.au/~/media/Corp/Documents/Health-for/Health-Networks/Disability/Hospital-Stay-Guidelines-Part-1.pdf> |
| WidgetHealth | <https://widgit-health.com/> | How to call an ambulance | [How to call an ambulance.pdf (widgit-health.com)](https://widgit-health.com/easy-read-sheets/pdfs/How%20to%20call%20an%20ambulance.pdf) |
|  |  | A&E communication passport | [A-E_Communincation_Passport_2013.pdf (widgit-health.com)](https://widgit-health.com/download-files/aande/2013/A-E_Communincation_Passport_2013.pdf) |
|  |  | Going to hospital (full symbols) | [Going to Hospital - full symbols.pdf (widgit-health.com)](https://widgit-health.com/easy-read-sheets/pdfs/Going%20to%20Hospital%20-%20full%20symbols.pdf) |
|  |  | Going to hospital (key symbols) | [Going to Hospital - key symbols.pdf (widgit-health.com)](https://widgit-health.com/easy-read-sheets/pdfs/Going%20to%20Hospital%20-%20key%20symbols.pdf) |
|  |  | Going to hospital checklist | [Going to Hospital - checklist.pdf (widgit-health.com)](https://widgit-health.com/easy-read-sheets/pdfs/Going%20to%20Hospital%20-%20checklist.pdf) |
|  |  | Leaving hospital (full symbols) | [Leaving Hospital - full symbols.pdf (widgit-health.com)](https://widgit-health.com/easy-read-sheets/pdfs/Leaving%20Hospital%20-%20full%20symbols.pdf) |
|  |  | Leaving hospital (key symbols) | [Leaving Hospital - key symbols.pdf (widgit-health.com)](https://widgit-health.com/easy-read-sheets/pdfs/Leaving%20Hospital%20-%20key%20symbols.pdf) |
|  |  | Leaving hospital checklist | <https://widgit-health.com/easy-read-sheets/pdfs/Leaving%20Hospital%20-%20checklist.pdf> |
|  |  | Blood pressure | [Blood pressure.pdf (widgit-health.com)](https://widgit-health.com/easy-read-sheets/pdfs/Blood%20pressure.pdf) |
|  |  | Checking your blood pressure | [Checking your blood pressure.pdf (widgit-health.com)](https://widgit-health.com/easy-read-sheets/pdfs/Checking%20your%20blood%20pressure.pdf) |
|  |  | My first eye test in hospital | [my_eye_test_at_hospital.pdf (widgit-health.com)](https://widgit-health.com/download-files/my-first-eye-test-at-the-hospital/new-files/my_eye_test_at_hospital.pdf) |
|  |  | My first eye test  at the hospital | [my_eye_test_at_hospital_checklist.pdf (widgit-health.com)](https://widgit-health.com/download-files/my-first-eye-test-at-the-hospital/new-files/my_eye_test_at_hospital_checklist.pdf) |
|  |  | My eye test at school | [my_eye_test_at_school.pdf (widgit-health.com)](https://widgit-health.com/download-files/my-first-eye-test-at-the-hospital/new-files/my_eye_test_at_school.pdf) |
|  |  | My eye test at school checklist | <https://widgit-health.com/download-files/my-first-eye-test-at-the-hospital/new-files/my_eye_test_at_school_checklist.pdf> |
|  |  | Exercise is good for you | [Exercise is good for you.pdf (widgit-health.com)](https://widgit-health.com/easy-read-sheets/pdfs/Exercise%20is%20good%20for%20you.pdf) |
|  |  | Healthy Living | [Widgit Health - Patient Easy Read Symbol Sheets (widgit-health.com)](https://widgit-health.com/easy-read-sheets/index.htm#healthyliving) |
|  |  | Nose bleeds | <https://widgit-health.com/easy-read-sheets/pdfs/Nose%20bleeds.pdf> |
|  |  | Take care in the sun | [Take care in the sun.pdf (widgit-health.com)](https://widgit-health.com/easy-read-sheets/pdfs/Take%20care%20in%20the%20sun.pdf) |
|  |  | My Medication Review | [Easy Ready Medicines Prompt (widgit-health.com)](https://widgit-health.com/download-files/medication-review/My%20Medication%20Review.pdf) |
|  |  | After seeing the dentist | [after-the-dentist (widgit-health.com)](https://widgit-health.com/download-files/dental-procedures/after-the-dentist.pdf) |
|  |  | Healthy Teeth | [Healthy teeth.pdf (widgit-health.com)](https://widgit-health.com/easy-read-sheets/pdfs/Healthy%20teeth.pdf) |
|  |  | Going to the doctors | [Going to the Doctors.pdf (widgit-health.com)](https://widgit-health.com/easy-read-sheets/pdfs/Going%20to%20the%20Doctors.pdf) |
|  |  | Hospital Procedures | [Widgit Health - Hospital Procedures (widgit-health.com)](https://widgit-health.com/downloads/hospital-procedures) |
|  |  | Having a Tracheostomy | <https://widgit-health.com/download-files/tracheostomy/Having-a-Tracheostomy-Widgit-Symbols.pdf> |
|  |  | Health check (key symbols) | [Health Check - key symbols.pdf (widgit-health.com)](https://widgit-health.com/easy-read-sheets/pdfs/Health%20Check%20-%20key%20symbols.pdf) |
|  |  | Health check (full symbols) | [Health Check - full symbols.pdf (widgit-health.com)](https://widgit-health.com/easy-read-sheets/pdfs/Health%20Check%20-%20full%20symbols.pdf) |
|  |  | My daycase operation (key symbols) | [child daycase operation booklet (widgit-health.com)](https://widgit-health.com/download-files/hospital-procedures/Child_Daycase_Operation_Booklet.pdf) |
|  |  | Skin Laser treatment | [Skin laser treatment.pdf (widgit-health.com)](https://widgit-health.com/easy-read-sheets/pdfs/Skin%20laser%20treatment.pdf) |
|  |  | Having an immunisation | <https://widgit-health.com/download-files/immunisation/immunisation-resource.pdf> |
|  |  | Arriving at the dentist | <https://widgit-health.com/download-files/dental-procedures/arriving-at-the-dentist.pdf> |
|  |  | In the dentist's room | [in-the-dentists-room (widgit-health.com)](https://widgit-health.com/download-files/dental-procedures/in-the-dentists-room.pdf) |
|  |  | Visting the dentist | [Visit to the Dentist.pdf (widgit-health.com)](https://widgit-health.com/easy-read-sheets/pdfs/Visit%20to%20the%20Dentist.pdf) |
|  |  | My sliver tooth | [my-silver-tooth (widgit-health.com)](https://widgit-health.com/download-files/dental-procedures/my-silver-tooth.pdf) |
|  |  | My plastic coating | [My plastic coating (widgit-health.com)](https://widgit-health.com/download-files/dental-procedures/my-plastic-coating.pdf) |
|  |  | My Dental Xray | <https://widgit-health.com/download-files/dental-xray/My-Extra-oral-Xray.pdf> |
|  |  | My small intra-oral Xray | [My-Small-Intra-oral-Xray.pdf (widgit-health.com)](https://widgit-health.com/download-files/dental-xray/My-Small-Intra-oral-Xray.pdf) |
|  |  | Having a blood test | [Having a blood test.pdf (widgit-health.com)](https://widgit-health.com/easy-read-sheets/pdfs/Having%20a%20blood%20test.pdf) |
|  |  | Having an EEG | [Having an EEG.pdf (widgit-health.com)](https://widgit-health.com/easy-read-sheets/pdfs/Having%20an%20EEG.pdf) |
|  |  | Barium swallow and meal | [Barium swallow and meal.pdf (widgit-health.com)](https://widgit-health.com/easy-read-sheets/pdfs/Barium%20swallow%20and%20meal.pdf) |
|  |  | Diabetes ABC cards | [Diabetes-Alphabet-Strategy-Symbols.pdf (widgit-health.com)](https://widgit-health.com/download-files/diabetes-abc/Diabetes-Alphabet-Strategy-Symbols.pdf) |
|  |  | Kidney stones | <https://widgit-health.com/easy-read-sheets/pdfs/Kidney%20stones.pdf> |
| Women with Disabilities Australia | <https://oursite.wwda.org.au/> | Healthcare rights | [Healthcare rights \| Life Choices \| Easy Read Webpage \| Our Site (wwda.org.au)](https://oursite-easyread.wwda.org.au/life-choices/healthcare-rights/) |
|  |  | Taking care of your health | [Taking care of your health \| Sex and Your Body \| Easy Read Webpage \| Our Site (wwda.org.au)](https://oursite-easyread.wwda.org.au/sex-and-your-body/taking-care-of-your-health/) |
|  |  | Women's health | [Women's Health \| Sex and Your Body \| Our Site (wwda.org.au)](https://oursite.wwda.org.au/sex-and-your-body/womens-health) |
|  |  | Pregnancy and Choices | [Pregnancy and Choices \| Sex and Your Body \| Our Site (wwda.org.au)](https://oursite.wwda.org.au/sex-and-your-body/pregnancy-and-choices) |
|  |  | Healthy Living | [Healthy Living \| Life Choices \| Our Site (wwda.org.au)](https://oursite.wwda.org.au/life-choices/healthy-living) |
| YORK University | <https://www.yorku.ca/> | Mental Health Literacy Guide for Autism | [Autism Mental Health Literacy Project (AM-HeLP) \| Developmental Disabilities and Mental Health Lab (yorku.ca)](https://www.yorku.ca/health/lab/ddmh/am-help/) |
|  |  |  |  |
